# Computer-aided design of a cyclic di-AMP synthesizing enzyme CdaA inhibitor

**DOI:** 10.1093/femsml/uqad021

**Published:** 2023-04-14

**Authors:** Piotr Neumann, Patrick Kloskowski, Ralf Ficner

**Affiliations:** Department of Molecular Structural Biology, Georg-August-University Goettingen, Institute of Microbiology and Genetics, GZMB, Justus-von-Liebig Weg 11, 37077 Goettingen, Germany; Department of Molecular Structural Biology, Georg-August-University Goettingen, Institute of Microbiology and Genetics, GZMB, Justus-von-Liebig Weg 11, 37077 Goettingen, Germany; Department of Molecular Structural Biology, Georg-August-University Goettingen, Institute of Microbiology and Genetics, GZMB, Justus-von-Liebig Weg 11, 37077 Goettingen, Germany

## Abstract

Cyclic di-AMP (c-di-AMP) is an essential secondary messenger regulating cell wall homeostasis and myriads of physiological processes in several Gram-positive and mycobacteria, including human pathogens. Hence, c-di-AMP synthesizing enzymes (DACs) have become a promising antibacterial drug target. To overcome a scarcity of small molecule inhibitors of c-di-AMP synthesizing enzyme CdaA, a computer-aided design of a new compound that should block the enzyme has been performed. This has led to the identification of a molecule comprising two thiazole rings and showing inhibitory potential based on ITC measurements. Thiazole scaffold is a good pharmacophore nucleus known due to its various pharmaceutical applications. It is contained in more than 18 FDA-approved drugs as well as in dozens of experimental drugs. Hence, the designed inhibitor can serve as a potent lead compound for further development of inhibitor against CdaA.

## Introduction

**Figure 1.**
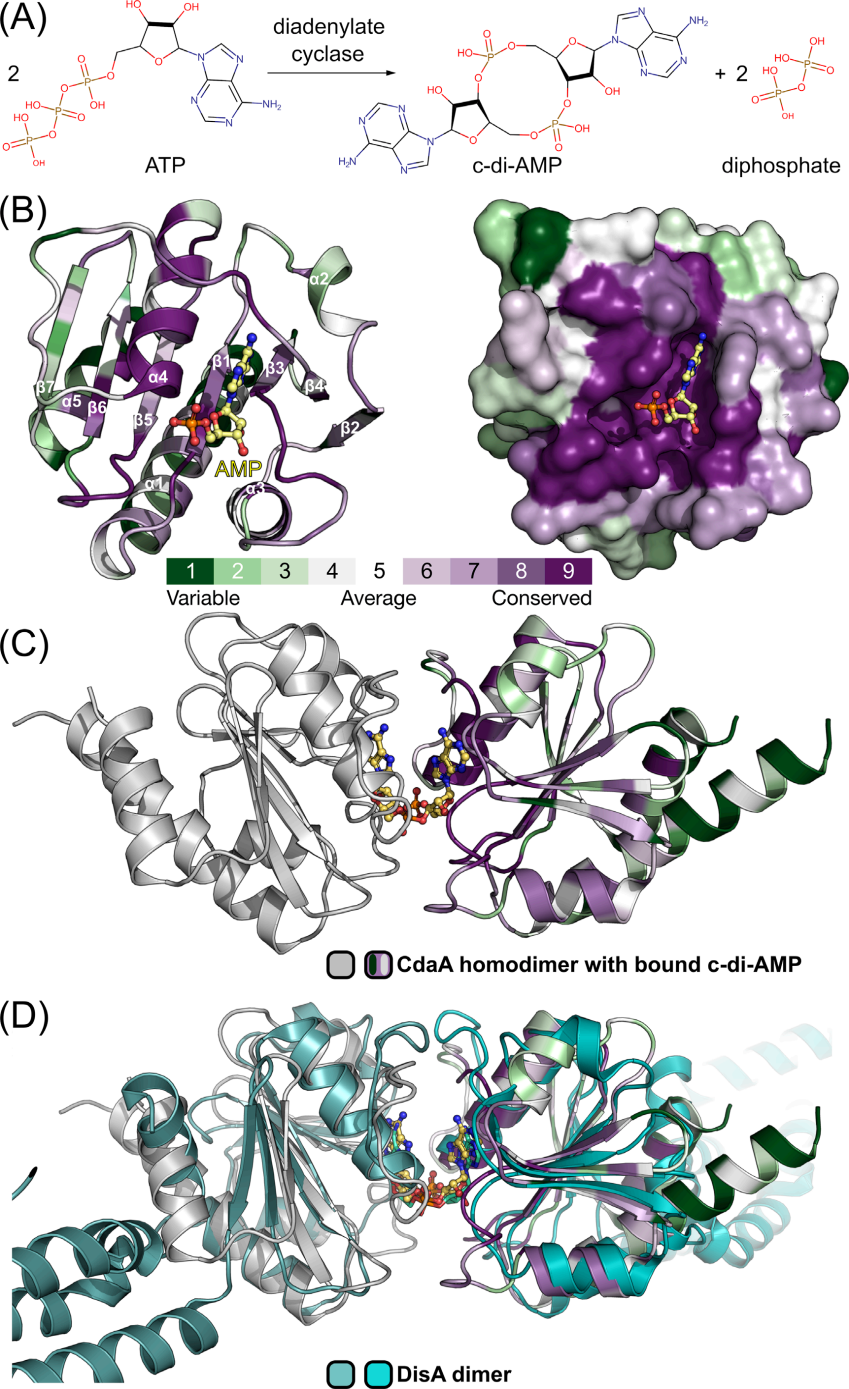
Crystal structure of Δ100CdaA. (A) Biochemical reaction of DAC; (B) overall structure of the truncated monomeric Δ100CdaA. The AMP molecule, depicted in ball and sticks mode marks the active site. Colour coding according to conservation score: green – low, white – medium, and purple – high. (C) Cartoon representation of the catalytically active Δ100CdaA dimer in its postcatalytic state with a c-di-AMP bound in the active site (PDB id: 6hvl). (D) Superposition of the Δ100CdaA dimer with DisA.

The misuse and overuse of antibiotics in concert with environmental transmission ensuant to inappropriate waste management have led to increased antimicrobial resistance (AMR) (Ventola, 2015, Byrne et al. 2019, Schrader et al. 2020) and associated bacterial persistence (Conlon et al. 2016, Harms et al. 2016). It is estimated that at least 700 000 people worldwide die each year due to drug-resistant infections with as much as 10 million by 2050 if the problem of AMR will not be addressed (AMR Industry Alliance 2020). Hence, the anticipated death toll caused by multi- and pan-drug resistant bacterial infections is a constantly growing public health threat, which without an effective antimicrobial treatment (Blaskovich 2020) will substantially impair most areas of modern medicine. The massive usage of antibiotics as a COVID-19 (co)treatment worldwide (Rawson et al. 2020, Zhou et al. 2020a, Vaughn et al. 2021), especially in case of hospitalized patients and those with already compromised immune systems (Zhou et al. 2020b), is predicted to add to ongoing emergence of AMR. This, perhaps overlooked aspect of concern related to the COVID-19, will most likely have further negative clinical consequences in the near future (Nieuwlaat et al. 2021). At present, the rise of antibiotic-resistant bacterial pathogens (Magiorakos et al. 2012) cannot be counteracted by the limited development of new antibiotics and therapeutics with new mode(s) of action (MoA(s)). Worldwide, at most 30–40 new antibacterial compounds are currently undergoing the clinical trial phases (Beyer and Paulin 2020). Hence, not only new agents featuring innovative chemistry and modes of action but also identification of novel targets are necessary to face the public health menace posed by AMR. The latter, namely identification of novel targets, has remained to be one of the major challenges of the antibiotic research, as a promising target should ideally be essential for the survival of a wide range of bacterial species and display neither structural nor functional homology to proteins of the mammalian host. Furthermore, target-based hampering of bacterial growth should not cause severe side effects (Silver 2011). In light of these circumstances the discovery of a unique essential secondary messenger, cyclic di-AMP (c-di-AMP; Fig. 1A) (Witte et al. 2008), in 2008 has opened new prospects in the field of antibiotic research. c-di-AMP is a bacterial signaling nucleotide, i.e. involved in regulation of the bacterial cellular processes like DNA integrity scanning, cell wall metabolism, osmolyte, and potassium ion homeostasis (Bai et al. 2013, Blötz et al. 2017, Gundlach et al. 2017, 2019). c-di-AMP synthesizing enzymes, the diadenylate cyclases (DACs), are essential for bacterial viability (Bai et al. 2013) as reported for *Streptococcus pneumoniae* (Song et al., 2005), *Listeria monocytogenes* (Woodward et al. 2010), and *Staphylococcus aureus* (Corrigan et al. 2011), hence they are potential targets of novel antimicrobial agents. Since its discovery in 2008, several studies have reported the presence of c-di-AMP in a wide range of different bacterial species, mainly in Gram-positive but in part also Gram-negative bacteria and archaea (Romling 2008, Corrigan and Gründling 2013). Many of these bacteria are known to be human pathogens e.g. *L. monocytogenes, Borrelia turicatea, S. aureus, Mycobacterium tuberculosis*, and *S. pneumoniae* (Woodward et al. 2010, Corrigan et al. 2011, Bai et al. 2012, Zarrella et al. 2020, Jackson-Litteken et al. 2021). The mentioned pathogens utilize only one class of DAC enzymes—namely CdaA, that catalyze the cyclization of two ATP molecules into c-di-AMP in a metal ion dependent manner (Fig. 1A) (Witte et al. 2008, Müller et al. 2015). Up to now, five different classes of DACs are known: CdaA, DisA, CdaS, CdaM, and CdaZ (Romling 2008, Corrigan and Gründling 2013, Blötz et al. 2017, Commichau et al. 2019), of which the first four have been structurally characterized. All of them share the highly conserved DAC domain (Fig. 1) accompanied by different types of regulatory domains (Commichau et al. 2019). Some bacteria like *Bacillus subtilis* carry more than one class of DACs, while most bacteria that are known to synthesize c-di-AMP possess only one, either DisA or CdaA. The latter is described as the most prevailing DAC domain containing protein among several bacterial species (Commichau et al. 2019). Interestingly, DACs are absent in mammalian cells, hence c-di-AMP cannot be detected in humans (Rosenberg et al. 2015). This renders CdaA a promising target for the antibiotic research.

CdaA is a membrane-bound protein consisting of the N-terminal transmembrane domain (residues 1–80) connected via a flexible linker (residues 11–100) with the catalytically active DAC domain (residues 101–255, numbering corresponds to *L. monocytogenes—Lm* CdaA). The structurally characterized DAC domain of *Lm* CdaA (∆100CdaA) shows an overall globular fold with a central seven-stranded mixed β-sheet flanked by five α-helices (Fig. 1B). The active site is formed by α-helix 4, the β-strands 1 and 5 as well as several loops connecting α1 and β1, α3 and β3, α4 and β4, and β5 and β6 (Fig. 1B). Generation of c-di-AMP requires a transient face to face dimerization of two ATP-loaded CdaA molecules (Fig. 1C) (Rosenberg et al. 2015, Heidemann et al. 2019), which leads to positioning of the two DAC domains in a way that strongly resembles the stably associated catalytically active dimer of DisA (Fig. 1C and D) (Witte et al. 2008, Heidemann et al. 2019). Despite this structural similarity, both catalytically active oligomers (DisA and dimerized CdaA) exhibit differences in lengths and amino-acid compositions of loops surrounding the active site (Fig. 1D). This might influence the accessibility to the ATP binding site due to steric hindrance. Since ATP-free CdaA exists in a noncatalytic state in solution and forms a transient homodimer in an ATP bound state to perform its catalytical action, it seems to be a more tractable target than DisA. CdaA is also the most prevailing c-di-AMP synthetizing enzyme among several bacterial species with a very well-conserved active site (Fig. 1B). Thus, compounds targeting the human pathogen *L. monocytogenes* CdaA would likely be effective against other CdaA dependent bacterial species, making it an ideal target for the development of new antibacterial therapeutics. Interestingly, three out of 12 families of bacteria that pose the greatest threat to human health according to WHO utilize CdaA as the sole enzyme synthesizing c-di-AMP.

To accelerate the development of antibacterial therapeutics targeting *L. monocytogenes* CdaA, we have recently determined several crystal structures to moderate and high resolutions in apo as well as AMP and postcatalytical c-di-AMP-bound states (Heidemann et al. 2019). In addition, crystal structures of *B. subtilis* (PDB id: 6HUW), *S. aureus* (PDB id: 6GYW), and *Streptococcus mutans* (PDB id: 7L8N) CdaAs are also available. These structures confirmed very high structural similarity to *Lm* CdaA as revealed by low root mean square deviation (r.m.s.d.), calculated between common Cα positions, of 0.55 Å and 0.51 Å, respectively. Structure based analysis in concert with biochemical data provided insights into the metal dependent catalytic mechanism of c-di-AMP formation and facilitated identification of the ATP binding site as the most promising druggable pocket on CdaA. This pocket is highly conserved across a variety of bacterial species (Fig. 1B), while the other surface regions reveal low or very low sequence conservation, and thus are not adequate for design of antibiotics active primarily against Gram-positive bacteria. Its ligandability has been confirmed by reported here crystal structures of CdaA with bound small molecular compounds mimicking/replacing a part of the substrate molecule. This approach provided structural information and chemical starting points for the structure-guided and computer-aided design of a new CdaA inhibitor that can be used for further rational design of antimicrobial therapeutics.

## Materials and methods

### Protein expression and purification

Protein expression and purification was based on the previously described protocol (Heidemann et al. 2019). Briefly, the plasmid pGEXpBP33, encoding a truncated *Lm*CdaA protein (∆100CdaA) with an N-terminal GST tag, was transformed into *Escherichia coli* BL21 (DE3). Resulting cell cultures were grown in 2xYT medium at 37°C. Expression was induced at OD_600_ of ∼0.6 by addition of 1 mM IPTG and the cultures were incubated overnight at 16°C. Harvested cells were disrupted with a microfluidizer (M-110S Microfluidizer, Microfluidics) in 20 mM Tris-HCl pH 7.5, 10 mM EDTA, 1 M NaCl, and then centrifuged at for 30 min at 4°C. Retained lysate, containing the GST-tagged target protein, was loaded onto a Glutathione Sepharose column (Cytiva) and eluted with 40 mm reduced GSH. The tag was proteolytically cleaved with 1:100 (w/w) PreScission protease overnight at 4°C. Remaining impurities and the tag were removed using a Superdex 75 (Cytiva) coupled to a Glutathione Sepharose column in 20 mM Tris-HCl pH 7.5, 300 mM NaCl.

### Crystallization

For crystallization the sitting-drop vapour-diffusion method was applied. The previously reported crystallization condition of the apo CdaA form (Heidemann et al. 2019) was optimized in order to yield crystals more suited for structure-aided drug design as judged based on better diffraction properties. The crystallization trials were performed at 20°C using a protein concentration of 5 mg/ml ∆100CdaA in 1 µl droplets and 1:1 protein-to-reservoir ratio. In order to facilitate crystal growth, micro seeding has been performed. Thin crystal plates were obtained overnight in 3.7 M NaCl, 0.1 M Na-HEPES pH 8.5, and 3% DMSO. The ideal DMSO concentration was determined by preceding stability tests.

### Computer-aided design of CdaA inhibitor using the OpenEye suite

Lead compound design has been performed using the OpenEye suite (OpenEye Scientific Software, Santa Fe, NM; https://www.eyesopen.com, academic free of charge license) and was primarily based on crystal structures of *Lm* CdaA complexed with AMP, ATP, and c-di-AMP. These structures provided specific CdaA structure-based spatial restraints (Tyr187-adenine ring π–π stacking, hydrogen bond restraints: adenine_N1-Leu188_N, adenine_N7-Leu188_O; Figure S1, Supporting Information) that were used during several cycles of exploring both chemical and property space around the bound adenine ring. The main aim of this approach was to find a molecule, i.e. chemically not similar to the substrate (ATP) but could effectively bind to the CdaA active site and restore the interaction pattern observed for the adenine moiety. Hence, short pieces of the incomplete AMP ribose moiety (atoms: C1’, C2’, O2’/C1’, C2’, O2’, C3’/C1’, C2’, O2’, C3’, O3’, and so on) have been kept as fixed anchoring points in BROOD (OpenEye Scientific Software; http://www.eyesopen.com), while searching for fragments that could replace the adenine ring under restraints of similar shape and preferably also electrostatics (EON, OpenEye Scientific Software). Over 30 of individual BROOD searches, differing in composition of chemical constraints like placement of donor/acceptor atoms and ring systems with specific properties, have been performed. These searches have led to identification of about 9000 nonunique clusters of compounds (several have been found in each search) of which a vast fraction was similar to adenine. The performed optimization of the search procedure (‘BROOD Query’) resulted in the two most promising BROOD searches of which graphical and tabulated summaries are presented in Figures S2 and S3 (Supporting Information) as well as in Tables S1 and S2 (Supporting Information), respectively. Tables S1 and S2 (Supporting Information) list several properties of the identified compounds like molecular weight, number of heavy atoms, ring ratio, XlogP (computed octanol–water partition coefficient), tPSA (topological polar surface area), Lipinski donors/acceptors/failures, number of rotors, and shape similarity. In addition, by incorporating the CdaA structural model originating from the AMP complex structure, the spatial restraints derived from the binding site have been imposed (π–π stacking interactions with Tyr187 side chain, no clashes between the tested compounds and protein environment, hydrogen bond restraints). Manual inspection of identified top scoring compounds (Figures S2 and S3, Supporting Information) resulted in keeping only those ligands, which are not chemically similar to adenine/AMP (set 1) and ideally comprise at least one six- or five-membered ring preferably containing a sulphur atom. In addition, the second set of substrate-like compounds (set 2) has been obtained using ROCS (Hawkins et al. 2007) (OpenEye Scientific Software) employing similar chemical restraints as for set 1. The shape-based superposition method, i.e. implemented in ROCS, has been used to find the best fitting compounds that could mimic truncated AMP and ATP molecules (Table S3, Supporting Information). Two multiconformer databases of compounds with ∼200 pregenerated conformations per molecule have been used. The first one was eMolecules_2017.2_maxconfs200.oeb, which is a standard OpenEye database comprising several millions of compounds. The second has been generated using OMEGA (Hawkins et al. 2010) (OpenEye Scientific Software) based on a set of over 10 000 drug molecules downloaded from the DrugBank (Wishart et al. 2018) database (https://go.drugbank.com/) and cured (conversion from SMILES format to sdf) using Open Babel software (http://openbabel.org/). Several hundreds of top scoring hits have been docked to CdaA using OEDOCKING (Kelley et al. 2015) (OpenEye Scientific Software) and subsequently rescored. This docking step was required to assess how well the identified molecules fit to the CdaA binding site (ROCS does not use the protein model as additional spatial restraints) irrespective whether they have been identified using BROOD or ROCS. A particularly useful feature of OEDOCKING was the classification of docking results based on multiple scoring criteria (total score, shape similarity, hydrogen bond pattern, and protein and ligand desolvation) expressed both as a number and as the percentage value, the latter providing a ranking relative to all other rescored molecules. The top rescored in OEDOCKING compounds (ranked at 100%) comprised a heterocyclic ring substructure (1,2-diazine) fused to a cyclohexane. The favoured by us thiazine derivatives have been ranked at 88% among all rescored compounds. The highest scoring 100 hits from each set (1 and 2) have been manually inspected in VIDA (OpenEye Scientific Software; http://www.eyesopen.com) and additionally rescored using the Gnina (McNutt et al. 2021) docking software utilizing an ensemble of convolutional neural networks (CNNs) as a scoring function. Comparison of OEDOCKING Hybrid Chemgauss4 score with the CNN affinity calculated by Gnina revealed that both rescoring approaches provide convergent results (Table S4, Supporting Information), hence are interchangeable. A collection of 40 compounds (Table S5, Supporting Information), that shared very low to moderate chemical similarity to adenine/AMP and simultaneously revealed high shape and electrostatic similarity, has been created. An additional structural requirement was the presence of at least one six- or five-membered ring preferably containing a sulphur atom. Most of these 40 compounds were not purchasable. For these compounds a similarity search in SciFinder (https://www.cas.org, Tanimoto coefficient of > = 0.75), under restriction ‘Commercially available’, has been conducted. For each searched compound up to 100 the most structurally similar and commercially available derivatives have been selected and stored in SMILES format. In part, that search has been performed in combination with the PubChem database (Kim et al. 2021). Conversion of canonical SMILES to the sdf format has been performed with Open Babel. The identified purchasable compounds (several thousands) have been subjected to molecular docking with Gnina (McNutt et al. 2021) software (Table S6, Supporting Information) against the structural model of CdaA AMP complex (chain B, Tyr187 in an orientation favouring π–π stacking). In total, four side chains have been kept flexible during docking process: Asp171, Tyr187, Thr202, and Glu223. The Gnina-based rescoring approach has been favoured over the OEDOCKING approach due to its flexibility concerning number of tested compounds, ease of scripting and the lack of necessity to generate multiconformer databases using OMEGA. Top scoring compounds have been analysed in Pymol. In total, five compounds have been purchased (Table S5, Supporting Information) and subjected to a crystallographic structural analysis.

### Crystal structure analysis

Stock solutions (1 M concentration) of the purchased compounds (Table S5, Supporting Information) have been prepared by dissolving those either in the reservoir solution supplemented with DMSO (compounds 1, 4, and 16) or in DMSO (7 and 14) and subsequently diluted to 100 mM in the reservoir. The reservoir solution has been prepared using a higher concertation of the buffering agent (0.5 M Na–HEPES pH 8.5) to prevent large pH changes that might be caused by the solubilized compounds. When necessary, pH has been adjusted by addition of small amounts of high concentrated HCl/NaOH. A volume of 1 µl of compound solution has been added to a 1 µl droplet containing CdaA crystals. Due to crystal stability issues, soaking time has been usually limited to 2–20 min, except for compound 16, which has been soaked overnight. The crystals have been cryo protected using a NaCl saturated reservoir solution prior to plunging them into liquid nitrogen. The same procedure has been applied for other ligands (adenine, adenosine, AMP, ATP) as well as apo CdaA (no soaking).

Diffraction images were collected at beamlines P13 and P14 operated by EMBL Hamburg at the PETRA III storage ring (DESY, Hamburg, Germany). All diffraction images were processed using an autoprocessing script (Neumann and Tittmann 2014) utilizing the XDS (Kabsch 2010) package. The structural models were refined by a self-written customized pipeline utilizing CCP4 (Winn et al. 2011) and PHENIX (Liebschner et al. 2019) suites. The atomic models have been manually adjusted in Coot (Emsley et al. 2010). Ligand molecules have been built into a difference mFo-DFc electron density map followed by further manual model adjustments and refinement using PHENIX suite. The polder mFo-DFc omit map (Liebschner et al. 2017) have been used to verify the presence of ligand molecules. Figures have been made using opensource version of pymol (www.pymol.org). Refinement and data collection statistics are shown in Table S7 (Supporting Information).

For some structures, crystallographic R factors are a bit elevated, most likely due to disordered N and C-termini, which were not built due to lack of interpretable electron density map. The missing fragments comprise residues 96–104 and 256–268, what amounts to almost 13% of all protein residues occupying the asymmetric unit. Atomic coordinates accompanied with diffraction data have been deposited in the PDB.

### Itc

ITC experiments were performed at 25°C and a stirring speed of 524 rpm on a MicroCal VP-ITC microcalorimeter (MicroCal Inc). Measurements were carried out with 50 μM CdaA in the sample cell and 1 mM of the analyzed ligand in the titration syringe (compounds 7 and 4, ATP, and c-di-AMP). Both, protein and ligands, were dissolved in the same buffer composed of 20 mM Tris-HCl pH 7.5, 300 mM NaCl. In case of compounds 7 and 4, the buffer has been supplemented with 2% DMSO, whereas 10 mM MgCl_2_ has been added in case of ATP and c-di-AMP. In the presence of Mg^2+^-ions, the metal-dependent *Lm* CdaA was shown to be not catalytically active (Heidemann et al. 2019). Furthermore, the common control experiments have been carried out: titrant to buffer, buffer to protein, and finally buffer to buffer. For compound 7, ATP, and c-di-AMP, the titrant to buffer experiments showed significant signals, which were considered in the subsequent analysis. Data was analyzed using the MicroCal PEAQ-ITC Analysis Software v1.41 (Malvern Panalytical) employing the single control method (subtraction of titrant to buffer experiment). For all performed experiments, the data sets were fit with a 1:1 binding model and yielded an assessment of the following thermodynamic parameters: dissociation constant (K_D_), a stoichiometry N, and the enthalpy of interaction ΔH.

## Results

### Crystal structures of *L. monocytogenes* CdaA

In our previous studies (Heidemann et al. 2019, Rosenberg et al. 2015) we have reported three polymorphic forms of *Lm* CdaA lacking the transmembrane domain and ∼30 amino acid long linker region (∆100CdaA). These crystal structures comprised a tetragonal ATP complex (PDB id: 4RV7, 2.8 Å resolution), hexagonal AMP and c-di-AMP complex in a postcatalytical state (PDB id: 6HVL, 2.8 Å resolution) as well as orthorhombic apo CdaA (PDB id: 6HVM, 2.0 Å resolution). In contrast to the relatively well-resolved apo structure, the crystals of CdaA complexes (ATP, AMP, and c-di-AMP) did not diffract well what impaired the level of structural details gained from them. Hence, we decided to obtain higher resolution crystal structures of orthorhombic apo CdaA (1.45 Å) and in addition AMP (1.75 Å) and ATP (1.97 Å) complexes (Table S7, Supporting Information) using a soaking approach. All monomeric ∆100CdaA feature the same basic architecture and conformation of the polypeptide chain with the root mean square deviation (r.m.s.d.) between common Cα positions ranging between 0.123 and 0.285 Å, when compared between ∆100CdaA apo and complex structures. The largest differences are observed between flexible N- and C-termini (residues 96–103 and 257–268) and a short seven amino acids long fragment (140–146). The latter flanks a beta strand harboring Tyr187 and changes its conformation from extended to alpha helical. Two ∆100CdaA monomers, present in the orthorhombic asymmetric unit, form a noncatalytic ‘back-to-back’ dimer (Figure S4, Supporting Information) with two active sites exposed to the solvent that makes them accessible for small molecular ligands. However, the active site of one monomer (chain B) is occupied by the side chain of Arg7 and two water molecules from the neighbouring CdaA molecule in the crystal lattice (Figure S4, Supporting Information). Hence, binding of ligands to chain B of orthorhombic CdaA crystals would require displacement of Arg7 and water molecules from the active site. Analysis of orthorhombic AMP, ATP and hexagonal c-di-AMP CdaA complexes reveals conformational flexibility of Tyr187^35^ side chain, i.e. crucial for catalytic activity. This residue adopts three distinct conformations (Figure S1, Supporting Information) of which one is able to lock the substrate's adenine ring in the binding pocket via π–π stacking interactions. Formation of these interactions requires, however, a subtle adjustment of tyrosine backbone, in particular the Cα–Cβ bond.

### The backrub motion of tyr187 backbone

**Figure 2.**
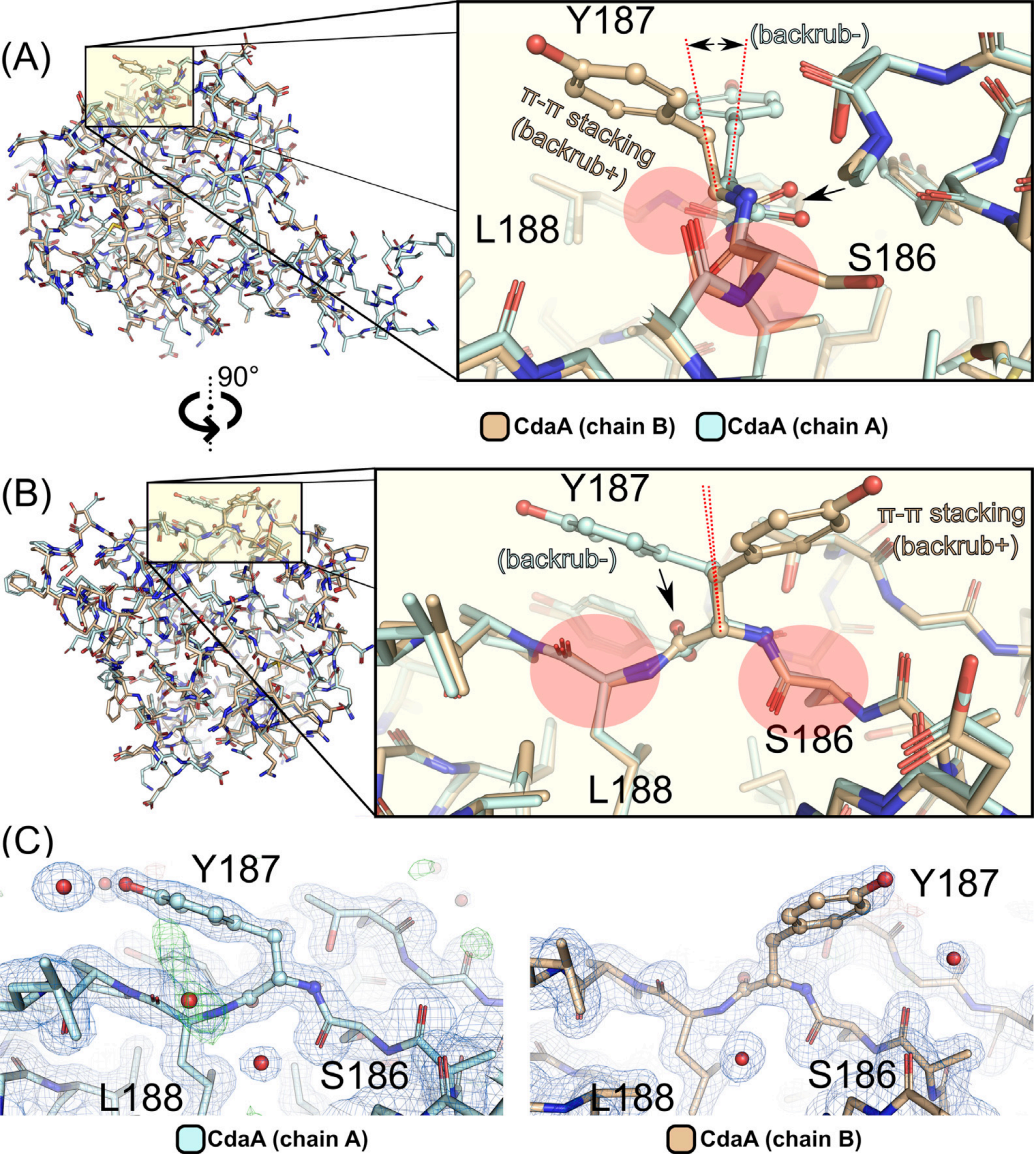
Backrub motion of Tyr187 positioned in CdaA active site. (A) Superposition of two monomers (A cyan and B wheat) occupying the asymmetric unit of apo CdaA structure (PDB id: 8C4Q) reveals conformational differences of the catalytically important active site residue Tyr187 (depicted as balls-and-sticks model). On the right: a close up view of Tyr187 and surrounding residues illustrating the structural details of the backrub motion (B) A different perspective (90° rotation) of the superposed CdaA monomers presented in (A) with accompanying close up view on the right. (C) Close-up views of Tyr187 in the two monomers of apo CdaA (orientation identical to (B), PDB id: 8C4Q). The calculated electron density maps (2mFo-DFc blue, 1.0 sigma level; mFo-DFc red/green, −3.0/3.0 sigma level) support the observed backrub motion of Tyr187 and reveal no alternative conformations of the Tyr187 side chain.

A thorough comparison of reported here high resolution apo *Lm* CdaA and all previously reported *Lm* CdaA crystal structures has revealed that in spite of the conformational flexibility of Tyr187 sidechain, formation of π–π stacking interactions with the adenine moiety bound in the active site necessitates a subtle local motion of protein backbone (Fig. 2). This motion results from concerted reorientation of two adjacent peptide bonds that swings the central Cα–Cβ bond and its sidechain (here Tyr187) in a direction perpendicular to the main chain without changing positions of flanking residues (Fig. 2). This subtle backbone plasticity coupled to changes of sidechain conformation is known in X-ray crystallography as ‘backrub’ motion (Davis et al. 2006). Backrubs-induced shifts are often of similar magnitude as experimental uncertainty. In case of reported here orthorhombic 1.45 Å resolution apo CdaA structure, with two molecules occupying the asymmetric unit (maximum likelihood coordinate error 0.21 Å), the observed differences in positions of Tyr187 Cα and Cβ atoms amount to 0.4 Å and 0.8 Å, respectively (Fig. 2). Comparison of previously determined apo CdaA (PDB id: 6HVM, maximum likelihood coordinate error 0.27 Å) and AMP/c-di-AMP bound CdaA monomers (PDB id: 6HVL, maximum likelihood coordinate error 0.4 Å) reveals the difference of position between Tyr187 Cα atoms of 0.8 Å. The observed subtle but structurally significant difference in orientation of Tyr187 Cα–Cβ bond has been taken into consideration during the design of structural restraints.

### Design of structural restraints

**Figure 3.**
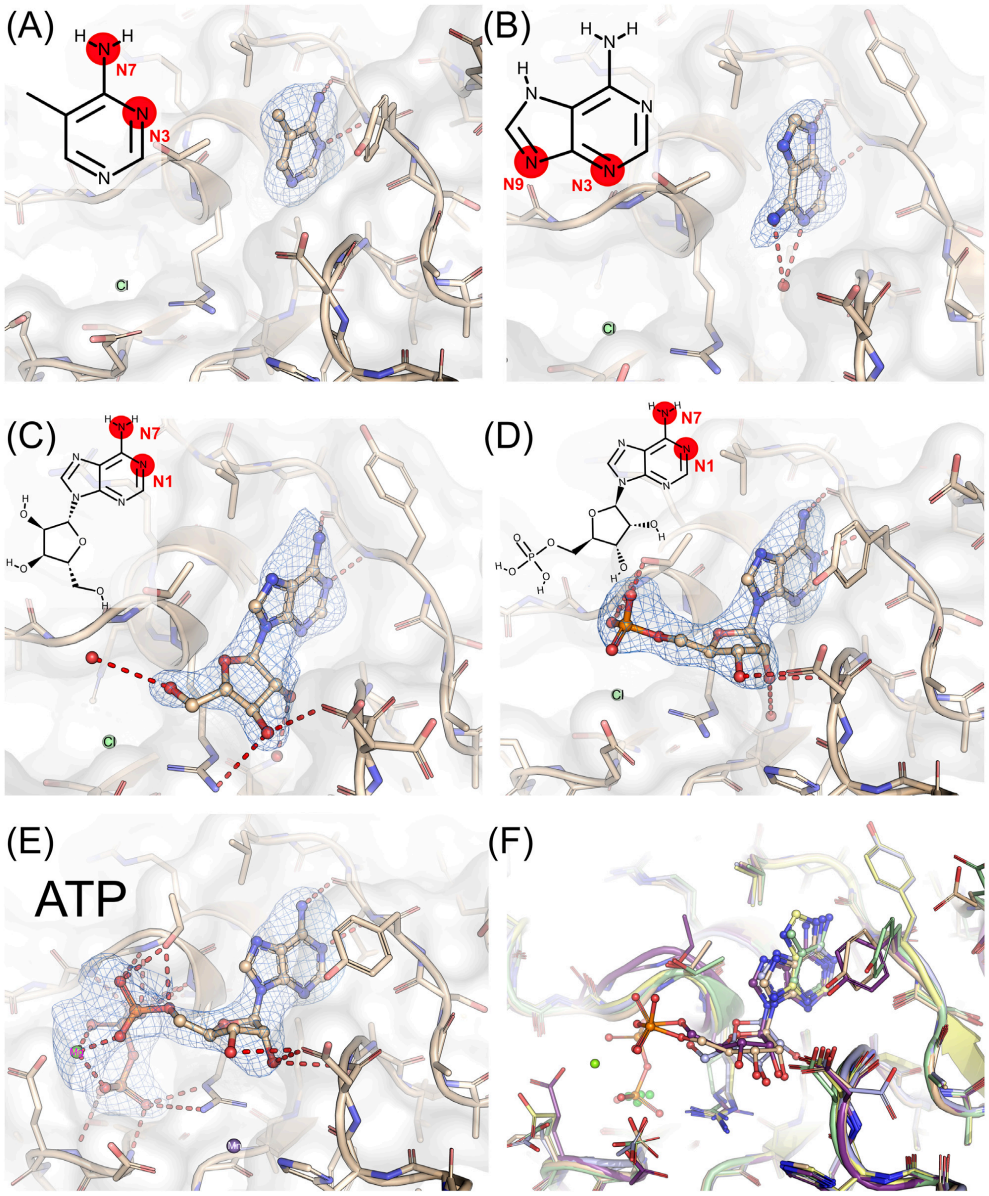
Design and structural verification of the ‘substrate-derived’ spatial restraints. Presented are crystal structures of CdaA complexes with ligands depicted in balls and sticks. For some ligands, chemical structure with corresponding atom numbering is presented. Polder omit mFo-DFc electron density map is contoured at >= 3 sigma level. (A) compound 4, 4-amino-5-methylpyrimidine; (B) adenine, (C) adenosine, (D) AMP, (E) ATP, and (F) superposition of all presented ligands.

The atomic model of the 1.45 Å resolution orthorhombic form of apo CdaA structure has confirmed the existence of two TYR 187 ‘backrub’ conformational states, as defined by orientation of its Cα–Cβ bond (Fig. 2). The productive one, termed here backrub+, facilitates the π–π stacking and is observed in molecule B of orthorhombic CdaA (chain B) but also in hexagonal AMP and c-di-AMP complex in a postcatalytical state (PDB id: 6HVL). The unproductive one (backrub-), observed in molecule A of orthorhombic CdaA (chain A) and in four monomers of tetragonal ATP complex (PDB id: 4rv7), is most likely favoured by the crystal lattice as binding of AMP or ATP molecules does not induce any productive backrub motions. Furthermore, comparison of available *Lm* CdaA complexes with AMP, ATP (Fig. 3), and the product c-di-AMP (Figure S1, Supporting Information) has revealed that the adenine ring forms two conserved hydrogen bonds (adenine_N1-Leu188_N and adenine_N7-Leu188_O) with the protein, irrespectively of the post or precatalytical state, Tyr187 backrub motion and π–π stacking interactions. This could be explained by the acid–base properties of nitrogen atoms constituting the adenine ring, of which N1 is the most basic one and exerts repulsion on the other nitrogen atoms (Kapinos et al. 2011). The two neighbouring nitrogen atoms (N1 and N7) of adenine moiety, forming hydrogen bonds with Leu188, have been used as strong donor and acceptor constraints during the lead compound design with the OpenEye suite (Fig. 3).

Having in hands both post and precatalytical structural snapshots of CdaA, we decided to target the precatalytical state of monomeric CdaA, i.e. able to lock the substrate in the active site by π–π stacking. Thus, similarly to the substrate, the designed molecule should ideally also be locked in the active site by stacking interactions (Heidemann et al. 2019) upon productive Tyr187 backrub motion (backrub+). The stacking interactions should additionally disfavour formation of the active homodimer that forces displacement of the Tyr187 side chain (rotamer change, no backrub movement, PDB id: 6HVL). Hence, design of CdaA inhibitor utilizing BROOD, OEDOCKING, Gnina, Pymol, and VIDA programs has been performed using the atomic model of monomeric CdaA originating from a well-resolved (1.75 Å) AMP complex crystal structure with a Tyr187 side chain placed optimally for π–π stacking (backrub+, chain B).

### Structural verification of the ‘substrate-derived’ spatial restraints

The validity of substrate-derived structural restraints has been assessed by solving crystal structures of CdaA complexes with small molecular compounds. The purpose of the first and the smallest soaked compound, 4-amino-5-methylpyrimidine (Table S5, Supporting Information: compound number 4; Fig. 3A), was to confirm the importance of two nitrogen atoms (N4 and N7) for substrate recognition and positioning in the CdaA active site. These two atoms form a structural pattern (…–N = C)_Aromatic_-NH_2_ abbreviated as -N = C-NH_2_. As the 4-amino group (N7) and N4 atom of the pyrimidine ring (Fig. 3A) are structurally equivalent to 6-amino group (N7) and N1 atom of the adenine ring (Fig. 3C and D), they should also be able to restore interaction pattern observed for the adenine moiety of ATP and AMP. Indeed, presented here CdaA 4-amino-5-methylpyrimidine complex structure (Fig. 3A) has confirmed that assumption and revealed that -N = C-NH_2_ structural pattern of the ligand molecule forms hydrogen bonds with the main chain atoms of Leu188 as observed for AMP and ATP (Fig. 3D and E). This implicates, that this group of atoms seems to be sufficient for both recognition and proper orientation of a molecule in the active site. Following that idea, we have determined crystal structures of CdaA complexes with adenine (Fig. 3B), adenosine (Fig. 3C) and compared those with newly determined high resolution structures of AMP (Fig. 3D) and ATP (Fig. 3E) complexes. The superposition of all structures (Fig. 3F) reveals the same binding orientation of the -N = C-NH_2_ group except for adenine, which utilizes isostructural, but chemically different aromatic N atoms (-N = C-N = ) as hydrogen bond partners for Leu188. Despite that difference, the binding mode is very similar. The lack of the π–π stacking interactions of adenine and adenosine with Tyr187 side chain results from the backrub-conformation as observed for molecule A (chain A). Why these two ligands could not be localized in the active site of the other CdaA molecule occupying the asymmetric unit (chain B—backrub+) is not clear and its elucidation is beyond the scope of this analysis. A plausible explanation is that weak binding ligands cannot replace the Arg7 side chain of the symmetry equivalent CdaA molecule occupying the active site of the molecule B with backrub+ conformation of Tyr187 (Figure S4, Supporting Information).

### Lead compound design

**Figure 4.**
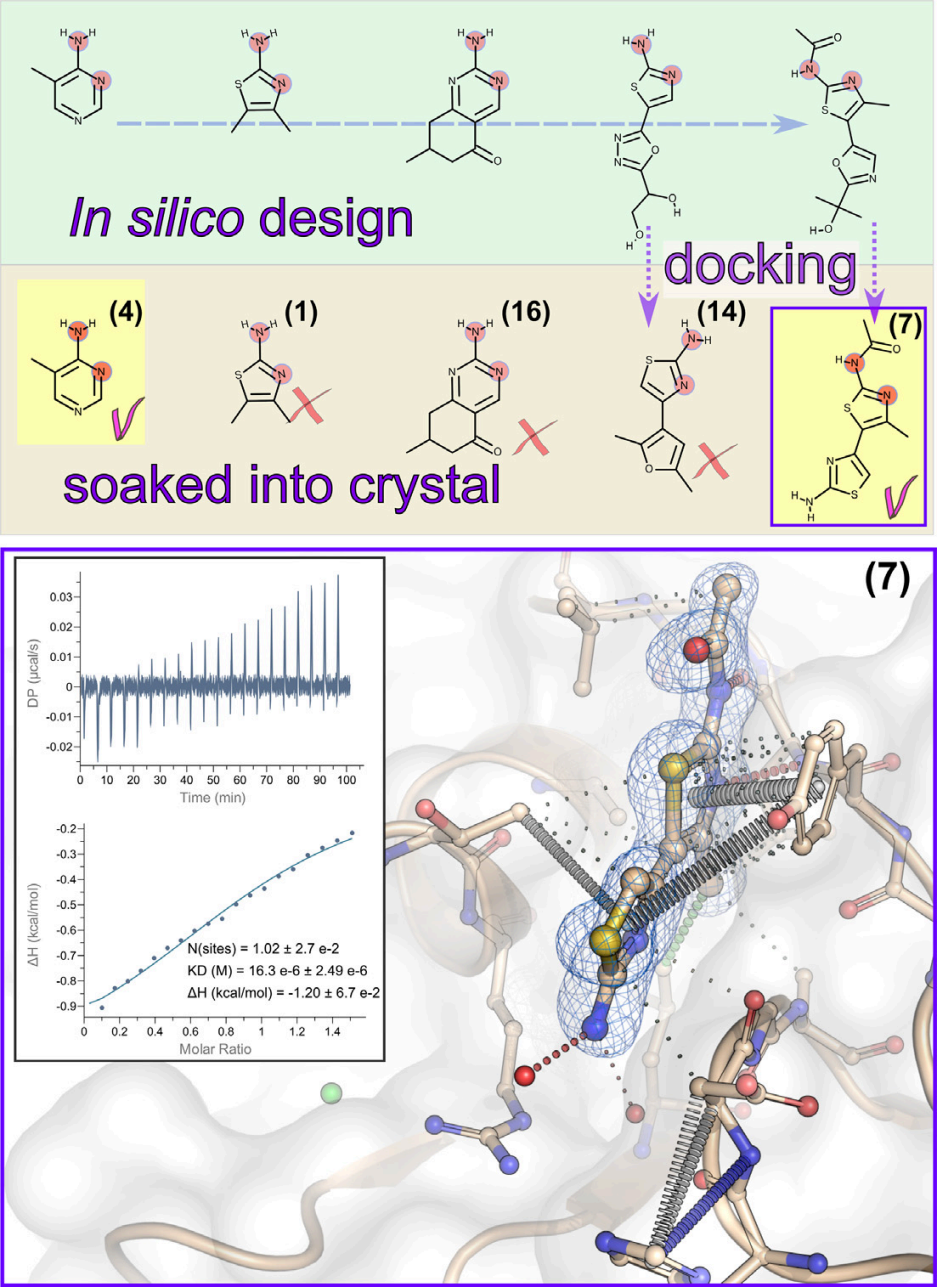
Lead compound design. Top. *In silico* design of the CdaA inhibitor using the OpenEye suite. Middle – compounds used for structural verification using soaking experiments and subsequent structure determination. Bottom – a close-up view of the compound number 7 (balls and sticks) bound in the CdaA active site with the polder omit mFo-DFc map contoured at 5 sigma level. Interactions between the ligand and protein atoms have been analysed using arpeggio web server. Inlet presents thermodynamic analysis of compound 7 binding to CdaA using isothermal titration calorimetry (ITC). The raw data thermogram (top) and the integrated heats (bottom) are presented. The data was fit with a 1:1 binding model and yielded a dissociation constant of KD = 16.3 ± 2.5 μM and a stoichiometry N of 1.02 ± 0.027, the latter being very close to its theoretically expected value of one.

Technical aspects of the computer-aided design of a CdaA inhibitor using the OpenEye suite (OpenEye Scientific Software; http://www.eyesopen.com) have been described in details in the ‘Materials and methods’ section. Briefly, the main goal of the described approach was the identification of a molecule that despite its substrate dissimilarity could still effectively bind to the active site of CdaA and restore the interaction pattern observed for the adenine moiety. Hence, strong hydrogen bond donor and acceptor positional restraints, resembling the -N = C-NH_2_ structural pattern, have been imposed in concert with an aromatic ring structure and cation π–π stacking when searching for compounds using the OpenEye suite (BROOD, ROCS) (Hawkins et al. 2007, Kelley et al. 2015). In order to enforce low similarity to the substrate molecule, all identified compounds that have been chemically similar to ATP or adenine have been discarded, including Type I kinase inhibitors known to be used for the treatment of human cancer and causing side effects, e.g.: crizotinib, dasatinib, erlotinib, gefitinib, lapatinib, pazopanib, and ruxolitinib. In addition, the focus has been set on compounds possessing a thiazole ring and in particular a 2-aminothiazole moiety preserving the -N = C-NH_2_ structural pattern used as restraints. The thiazole scaffold is a good pharmacophore nucleus (Petrou et al. 2021) and is contained in several biomolecules, hence it is not surprising that it has been identified in 100 drug molecules (the DrugBank (Wishart et al. 2018) search). Utilizing the above described restraints and constraints, a set of 40 compounds (Table S5, Supporting Information) has been identified as potential CdaA binders. All possessed the -N = C-NH_2_ structural pattern and due to their size differences, they were predicted to occupy different fractions of the CdaA binding site (Fig. 4). Most of them were not readily purchasable, hence their structurally close purchasable derivatives have been identified based on a similarity search in SciFinder (https://www.cas.org). These compounds have been subjected to molecular docking program Gnina (McNutt et al. 2021) in order to assess their predicted binding mode and affinity. The docking results have been manually inspected in Pymol and for the best docking decoys intermolecular interactions have been analyzed using arpeggio (Jubb et al. 2017). In total, five compounds, that have been both affordable and readily available, have been selected for structural analysis using the soaking approach: 1, 4, 7, 14, and 16 (Fig. 4; Table S5, Supporting Information). For three of those: 1, 16, and 14, no structural data have been obtained due to instability of crystals in soaking solutions as well as limited compound solubility in a high NaCl concentrated reservoir solution. The largest designed compound (number 7) comprising two substituted thiazole rings, has been predicted to fill the vast portion of the CdaA active site. This molecule has been unambiguously identified in the active site based on calculated high resolution (1.25 Å) omit mFo-DFc electron density map (Fig. 4). Its binding to CdaA seems to be strong as inferred based on the electron density map appearance, refined occupancy (1.0) and low atomic displacement factors (ADPs) being in the same range as these of protein atoms constituting the active site. This observation inspired us to assess its binding affinity by measuring the equilibrium dissociation constant (K_D_) using ITC (Fig. 4) and compare it with the K_D_ of the natural substrate ATP, product c-di-AMP, and compound 4 (Figure S5, Supporting Information). The last two ligands (compound 4 and c-di-AMP) reveal a very low binding affinity that cannot be reliably assessed based on performed ITC measurements (see description of Figure S5, Supporting Information). The ITC results confirmed that the compound number 7, binding to CdaA in the micromolar range and with ∼8 times lower K_D_ than that of ATP, can serve as a promising lead compound for further optimization.

## Discussion

One of the major challenges of the antibiotic research is the identification of new drug targets that are essential for survival, growth and proliferation of causative bacteria. Recent discovery of c-di-AMP has paved the way for starting the development of antibiotics targeting c-di-AMP synthesizing enzymes. These enzymes, possessing a well-conserved DAC domain, are absent in humans but present in a variety of pathogenic bacteria (Song et al. 2005, Woodward et al. 2010, Corrigan et al. 2011, Bai et al. 2012), e.g.: *L. monocytogenes, M. tuberculosis, B. turicatea, Enterococcus faecium, S. aureus*, and *S. pneumoniae*. The last three utilize a DAC containing CdaA as the sole enzyme responsible for c-di-AMP synthesis and belong to the 12 families of bacteria that pose the greatest threat to human health according to WHO. Hence, design of DAC domain inhibitors has become a subject of recent effort of several research groups that concentrate on finding inhibitors not only against CdaA but also against another DAC enzyme—DisA. Recently, a few DisA inhibitors like antiparasitic drug suramine (Opoku-Temeng and Sintim 2016a), bromophenol (Zheng et al. 2014, 2016), polyphenols (Opoku-Temeng and Sintim 2016b), and hydroxybenzylidene–indolinones (Opoku-Temeng et al. 2017) have been reported. Despite the undeniable structural similarity between DAC domains of DisA and CdaA, the majority of DisA inhibitors do not show inhibitory effects on CdaA, except of bromophenol (Chen et al. 2018), which was shown to inhibit both DAC domain containing enzymes. The second known CdaA inhibitor, IPA-3 (2,2′-dihydroxy-1,1′-dinapthyldisulfide) (Li et al. 2022), containing naphthyl groups, has revealed potent antimicrobial activity. Unfortunately, no structural data has been obtained for any of those inhibitors, hence structure-based optimization of those compounds is not yet feasible. Our own efforts to structurally characterize the aforementioned CdaA inhibitors were unsuccessful.

Therefore, in our study, we have focused on structural characterization of small molecular ligands in order to find a promising lead compound that could be further optimized. The main goal was to design an inhibitor comprising thiazole scaffold, a good pharmacophore nucleus, i.e. known due to its many biologically active derivatives with various pharmaceutical applications such as antibacterial, antifungal, antimalarial, anticancer, antiallergic, antihypertensive, and anti-inflammatory (Petrou et al. 2021). In fact, the application range is even wider and comprises antipsychotic, antioxidant, and analgesic agents. This makes the identified compound 7 a promising starting point for further structure-based optimization that will further improve its binding affinity, preferably to the nanomolar range. There are several strategies that can be undertaken, among others performing a crystallographic fragment screen enabling an efficient exploration of chemical space (Hall et al. 2014). Optimization of hydrophobic interactions, that are one of the main driving forces in drug–receptor interactions, is another possibility. It has been estimated that the benefit of burying one solvent-exposed methyl group into a hydrophobic pocket of a protein is a 3.2-fold increase in binding constant or about 0.7 kcal/mol (Davis and Teague 1999). However, any modification like replacement of a hydrogen atom with a methyl group is highly context dependent, and potency losses are as common as gains.

In conclusion, we have assessed drugability and sequence conservation of CdaA confirming the ATP binding pocket as the most conserved region of CdaA among several bacterial species. This makes CdaA a promising target for the development of antibacterial therapeutics that may help to combat AMR posing a huge threat to public health. Our computer-aided design of a novel inhibitor resulted in identification of a molecule binding to CdaA in the micromolar range and with ∼8 times lower K_D_ than that of the natural substrate ATP. This is the first structurally characterized DAC inhibitor, comprising the biologically active thiazole scaffold, that makes it a very promising lead compound for further optimization.

## Supplementary Material

uqad021_Supplemental_File

## Data Availability

Atomic coordinates and structure factors of the crystal structures presented in this article were deposited in the Protein Data Bank (PDB) with the following codes: 8C4Q (CdaA-apo), 8C4O (CdaA-ATP), 8C4N (CdaA-AMP), 8C4R (CdaA-adenine), 8C4M (CdaA-adenosine), 8C4J (CdaA-compound 4), and 8C4P (CdaA-compound 7).
