# Supplementary material for: Computer-aided design of a cyclic di-AMP synthesizing enzyme CdaA inhibitor
Source: Microlife. 2023 Apr 14;4:uqad021. doi: 10.1093/femsml/uqad021 (PMC10167629; doi:10.1093/femsml/uqad021)
Supplement: uqad021_Supplemental_File [file uqad021_supplemental_file.docx]

**Supplemental Information**

**Table S1.** Properties of compounds identified in an exemplary BROOD search presented as a graphical summary on Figure S2

| **VIDA Name** | **ID** | **Ring Ratio** | **XLogP** | **Heavy Atoms** | **Molecular Weight [g/mol]** | **tPSA** | **Lipinski Donors** | **Lipinski**  **Acceptors** | **Rotors** | **Lipinski Failures** | **shape** | **Source Mol Labels** |
| --- | --- | --- | --- | --- | --- | --- | --- | --- | --- | --- | --- | --- |
| Query Analogs# 1 of 157 | 4 | 0.69 | -0.8 | 13 | 178.19 | 76.44 | 2 | 5 | 2 | 0 | 0.98 | CHEMBL49046 |
| Cluster 1# 1 of 1 | 161 | 0.75 | -0.2 | 16 | 218.26 | 76.44 | 2 | 5 | 3 | 0 | 0.82 | CHEMBL1836745 |
| Cluster 2# 1 of 15 | 162 | 0.6 | -0.5 | 15 | 207.23 | 75.86 | 2 | 6 | 4 | 0 | 0.89 | CHEMBL1968016 |
| Cluster 3# 1 of 26 | 177 | 0.6 | -0.6 | 15 | 209.23 | 75.05 | 2 | 6 | 3 | 0 | 0.9 | CHEMBL331061 |
| Cluster 4# 1 of 4 | 203 | 0.75 | -0.4 | 12 | 164.16 | 74.69 | 2 | 5 | 2 | 0 | 0.87 | CHEMBL1724918 |
| Cluster 5# 1 of 34 | 207 | 0.75 | -1.4 | 12 | 168.22 | 53.35 | 3 | 4 | 2 | 0 | 0.86 | CHEMBL551482 |
| Cluster 6# 1 of 11 | 241 | 0.56 | 0.2 | 16 | 221.26 | 75.86 | 2 | 6 | 3 | 0 | 0.89 | CHEMBL1561076 |
| Cluster 7# 1 of 5 | 252 | 0.69 | -1.2 | 13 | 181.17 | 84.53 | 3 | 6 | 2 | 0 | 0.81 | CHEMBL3127141 |
| Cluster 8# 1 of 1 | 257 | 0.69 | -1.3 | 13 | 183.18 | 76.02 | 3 | 5 | 2 | 0 | 0.94 | CHEMBL22704 |
| Cluster 9# 1 of 43 | 258 | 0.6 | 0.1 | 15 | 224.28 | 76.96 | 2 | 5 | 3 | 0 | 0.9 | CHEMBL160248 |
| Cluster 10# 1 of 3 | 301 | 0.64 | -1.8 | 14 | 196.21 | 82.67 | 3 | 6 | 3 | 0 | 0.95 | CHEMBL157130 |
| Cluster 11# 1 of 2 | 304 | 0.6 | -0.5 | 15 | 208.22 | 83.54 | 2 | 6 | 3 | 0 | 0.89 | CHEMBL45919 |
| Cluster 12# 1 of 12 | 306 | 0.56 | -0.6 | 16 | 224.24 | 88.55 | 3 | 7 | 3 | 0 | 0.82 | CHEMBL507423 |
| Cluster 13# 1 of 4 | 318 | 0.64 | -0.3 | 14 | 194.19 | 83.28 | 2 | 6 | 2 | 0 | 0.89 | CHEMBL1520 |
| Cluster 14# 1 of 5 | 322 | 0.6 | 0.1 | 15 | 207.23 | 77.93 | 2 | 6 | 3 | 0 | 0.72 | CHEMBL52036 |
| Cluster 15# 1 of 5 | 327 | 0.6 | 0.1 | 15 | 217.22 | 52.15 | 2 | 4 | 2 | 0 | 0.79 | CHEMBL1082451 |
| Cluster 16# 1 of 6 | 332 | 0.56 | -0.3 | 16 | 223.23 | 89.91 | 2 | 7 | 3 | 0 | 0.84 | CHEMBL3086484 |
| Cluster 17# 1 of 10 | 338 | 0.64 | -0.4 | 14 | 196.27 | 53.35 | 3 | 4 | 3 | 0 | 0.8 | CHEMBL250204 |
| Cluster 18# 1 of 7 | 348 | 0.64 | -0.4 | 14 | 218.64 | 67.51 | 2 | 5 | 2 | 0 | 0.86 | CHEMBL1645038 |
| Cluster 19# 1 of 9 | 355 | 0.64 | -1.7 | 14 | 195.18 | 99.43 | 3 | 7 | 2 | 0 | 0.88 | CHEMBL2136817 |
| Cluster 20# 1 of 2 | 364 | 0.6 | -0.2 | 15 | 209.2 | 80.4 | 2 | 6 | 3 | 0 | 0.87 | CHEMBL2134314 |
| Cluster 21# 1 of 6 | 366 | 0.64 | -2.3 | 14 | 198.24 | 73.58 | 4 | 5 | 3 | 0 | 0.79 | CHEMBL109522 |
| Cluster 22# 1 of 1 | 372 | 0.67 | -1.1 | 15 | 213.28 | 78.34 | 3 | 5 | 4 | 0 | 0.69 | CHEMBL1774284 |
| Cluster 23# 1 of 3 | 373 | 0.6 | -0.6 | 15 | 210.28 | 67.31 | 2 | 5 | 2 | 0 | 0.84 | CHEMBL2058835 |
| Cluster 24# 1 of 1 | 376 | 0.6 | -0.6 | 15 | 210.28 | 67.31 | 2 | 5 | 2 | 0 | 0.84 | CHEMBL2058834 |
| Cluster 25# 1 of 4 | 377 | 0.64 | -1.2 | 14 | 197.21 | 84.14 | 4 | 6 | 3 | 0 | 0.8 | CHEMBL146768 |
| Cluster 26# 1 of 1 | 381 | 0.75 | -1.3 | 12 | 166.2 | 58.59 | 3 | 4 | 2 | 0 | 0.88 | CHEMBL2297802 |
| Cluster 27# 1 of 3 | 382 | 0.56 | -0.3 | 16 | 220.23 | 80.04 | 2 | 6 | 4 | 0 | 0.73 | CHEMBL67805 |
| Cluster 28# 1 of 4 | 385 | 0.6 | -0.6 | 15 | 212.27 | 63.89 | 2 | 5 | 4 | 0 | 0.74 | CHEMBL454364 |
| Cluster 29# 1 of 3 | 389 | 0.56 | 0.5 | 16 | 222.24 | 83.28 | 2 | 6 | 4 | 0 | 0.78 | CHEMBL1520 |
| Cluster 30# 1 of 1 | 392 | 0.56 | 0.6 | 16 | 221.26 | 86.72 | 3 | 6 | 5 | 0 | 0.68 | CHEMBL52036 |
| Cluster 31# 1 of 1 | 393 | 0.56 | -1.4 | 16 | 223.19 | 126.37 | 3 | 8 | 3 | 0 | 0.84 | CHEMBL2000466 |
| Cluster 32# 1 of 2 | 394 | 0.69 | -1.1 | 13 | 184.26 | 57.07 | 3 | 3 | 2 | 0 | 0.76 | CHEMBL1940585 |
| Cluster 33# 1 of 2 | 396 | 0.42 | -1.4 | 12 | 171.15 | 101.11 | 3 | 6 | 3 | 0 | 0.76 | CHEMBL404531 |
| Cluster 34# 1 of 2 | 398 | 0.73 | -1.1 | 11 | 154.19 | 53.35 | 3 | 4 | 2 | 0 | 0.74 | CHEMBL204090 |
| Cluster 35# 1 of 1 | 400 | 0.45 | -0.2 | 11 | 174.22 | 66.24 | 2 | 4 | 4 | 0 | 0.72 | CHEMBL1572894 |
| Cluster 36# 1 of 8 | 401 | 0.45 | -2.3 | 11 | 162.21 | 65.13 | 4 | 4 | 3 | 0 | 0.68 | CHEMBL2018338 |
| Cluster 37# 1 of 2 | 409 | 0.38 | -1.9 | 13 | 186.21 | 78.51 | 3 | 5 | 4 | 0 | 0.73 | CHEMBL1971350 |
| Cluster 38# 1 of 1 | 411 | 0.54 | -1.2 | 13 | 185.24 | 67.76 | 3 | 4 | 3 | 0 | 0.75 | CHEMBL423317 |
| Cluster 39# 1 of 7 | 412 | 0.42 | -1.6 | 12 | 173.26 | 65.13 | 4 | 4 | 4 | 0 | 0.66 | CHEMBL119951 |
| Cluster 40# 1 of 2 | 419 | 0.58 | -1.6 | 12 | 176.23 | 65.13 | 4 | 4 | 2 | 0 | 0.79 | CHEMBL84901 |
| Cluster 41# 1 of 3 | 421 | 0.42 | -1.6 | 12 | 176.23 | 77.3 | 4 | 4 | 3 | 0 | 0.69 | CHEMBL85171 |
| Cluster 42# 1 of 1 | 424 | 0.54 | -2.3 | 13 | 186.23 | 71 | 3 | 5 | 3 | 0 | 0.69 | CHEMBL2326188 |
| Cluster 43# 1 of 1 | 425 | 0.67 | -1.4 | 12 | 173.23 | 60.31 | 3 | 4 | 2 | 0 | 0.84 | CHEMBL482549 |
| Cluster 44# 1 of 1 | 426 | 0.62 | -1.6 | 13 | 188.24 | 65.13 | 4 | 4 | 2 | 0 | 0.77 | CHEMBL1917361 |
| Cluster 45# 1 of 1 | 427 | 0.69 | -0.9 | 13 | 183.23 | 78.41 | 3 | 5 | 3 | 0 | 0.6 | CHEMBL567559 |
| Cluster 46# 1 of 2 | 428 | 0.71 | -1.3 | 14 | 200.2 | 93.29 | 2 | 7 | 3 | 0 | 0.81 | CHEMBL1993354 |
| Cluster 47# 1 of 1 | 430 | 0.55 | -0.3 | 11 | 160.23 | 68.1 | 3 | 3 | 2 | 0 | 0.62 | CHEMBL334479 |
| Cluster 48# 1 of 3 | 431 | 0.5 | -1.6 | 12 | 176.23 | 65.13 | 4 | 4 | 2 | 0 | 0.63 | CHEMBL2011633 |
| Cluster 49# 1 of 2 | 434 | 0.46 | -2.9 | 13 | 191.25 | 91.57 | 4 | 5 | 3 | 0 | 0.68 | CHEMBL205036 |
| Cluster 50# 1 of 1 | 436 | 0.69 | -2.1 | 13 | 188.24 | 54.13 | 3 | 4 | 2 | 0 | 0.61 | CHEMBL3087512 |
| Cluster 51# 1 of 1 | 437 | 0.67 | -0.9 | 12 | 172.24 | 44.9 | 3 | 3 | 2 | 0 | 0.62 | CHEMBL32156 |
| Cluster 52# 1 of 1 | 438 | 0.67 | -0.6 | 12 | 172.24 | 44.9 | 3 | 3 | 2 | 0 | 0.63 | CHEMBL469429 |
| Cluster 53# 1 of 1 | 439 | 0.62 | 0.1 | 13 | 186.27 | 44.9 | 3 | 3 | 2 | 0 | 0.65 | CHEMBL3084681 |
| Cluster 54# 1 of 1 | 440 | 0.54 | -3 | 13 | 192.23 | 85.36 | 5 | 5 | 2 | 0 | 0.79 | CHEMBL84901 |

**Table S2.** Properties of compounds yielded by a BROOD search (graphical summary on Figure S3) leading to identification of the precursor of compound 7 (**Cluster 5#**).

| **VIDA Name** | **ID** | **Ring Ratio** | **XLogP** | **Heavy Atoms** | **Molecular Weight [g/mol]** | **tPSA** | **Lipinski Donors** | **Lipinski**  **Acceptors** | **Rotors** | **Lipinski Failures** | **shape** | **Source Mol Labels** |
| --- | --- | --- | --- | --- | --- | --- | --- | --- | --- | --- | --- | --- |
| Query Analogs#  1 of 4 | 4 | 0.6 | -1.12 | 15 | 209.23 | 97.92 | 4 | 6 | 3 | 0 | 0.94 | CHEMBL3290651 |
| Query Analogs#  2 of 4 | 5 | 0.56 | -1 | 16 | 224.22 | 103.77 | 3 | 7 | 3 | 0 | 0.91 | CHEMBL370962 |
| Query Analogs#  3 of 4 | 6 | 0.56 | -1.27 | 16 | 223.23 | 96.09 | 3 | 7 | 4 | 0 | 0.89 | CHEMBL84945 |
| Query Analogs#  4 of 4 | 7 | 0.6 | -2.13 | 15 | 210.19 | 104.03 | 3 | 7 | 3 | 0 | 0.84 | CHEMBL3250884 |
| Cluster 1# 1 of 1 | 8 | 0.82 | -0.57 | 17 | 235.24 | 80.4 | 2 | 6 | 2 | 0 | 0.74 | CHEMBL1791037 |
| Cluster 2# 1 of 6 | 9 | 0.64 | -1.52 | 14 | 193.2 | 79.45 | 2 | 5 | 2 | 0 | 0.76 | CHEMBL2419506 |
| Cluster 2# 2 of 6 | 10 | 0.64 | 0.32 | 14 | 212.27 | 60.69 | 3 | 3 | 2 | 0 | 0.83 | CHEMBL187591 |
| Cluster 2# 3 of 6 | 11 | 0.64 | -2.44 | 14 | 194.19 | 92.34 | 2 | 6 | 2 | 0 | 0.75 | CHEMBL1774909 |
| Cluster 2# 4 of 6 | 12 | 0.64 | -0.84 | 14 | 191.23 | 72.03 | 2 | 4 | 2 | 0 | 0.82 | CHEMBL1408419 |
| Cluster 2# 5 of 6 | 13 | 0.69 | -1.77 | 13 | 181.22 | 76.47 | 3 | 5 | 2 | 0 | 0.8 | CHEMBL1869488 |
| Cluster 2# 6 of 6 | 14 | 0.6 | -0.6 | 15 | 205.26 | 72.03 | 2 | 4 | 2 | 0 | 0.8 | CHEMBL1408419 |
| Cluster 3# 1 of 1 | 15 | 0.67 | -0.44 | 15 | 205.21 | 73.58 | 3 | 4 | 2 | 0 | 0.71 | CHEMBL554636 |
| Cluster 4# 1 of 3 | 16 | 0.62 | -0.22 | 16 | 219.24 | 73.58 | 3 | 4 | 3 | 0 | 0.83 | CHEMBL1878466 |
| Cluster 4# 2 of 3 | 17 | 0.62 | 0.78 | 16 | 219.24 | 62.58 | 2 | 4 | 3 | 0 | 0.84 | CHEMBL545255 |
| Cluster 4# 3 of 3 | 18 | 0.67 | 0.36 | 15 | 204.23 | 68.37 | 2 | 4 | 3 | 0 | 0.8 | CHEMBL1398487 |
| **Cluster 5# 1 of 5** | **19** | **0.77** | **-0.41** | **13** | **197.21** | **85.17** | **2** | **5** | **2** | **0** | **0.67** | **CHEMBL2204787** |
| **Cluster 5# 2 of 5** | **20** | **0.77** | **-0.52** | **13** | **198.2** | **79.38** | **2** | **5** | **2** | **0** | **0.67** | **CHEMBL2204776** |
| **Cluster 5# 3 of 5** | **21** | **0.77** | **-0.41** | **13** | **197.21** | **85.17** | **2** | **5** | **2** | **0** | **0.67** | **CHEMBL2203302** |
| **Cluster 5# 4 of 5** | **22** | **0.71** | **-0.17** | **14** | **211.24** | **85.17** | **2** | **5** | **2** | **0** | **0.63** | **CHEMBL2204787** |
| **Cluster 5# 5 of 5** | **23** | **0.77** | **0.38** | **13** | **195.24** | **74.93** | **3** | **4** | **2** | **0** | **0.66** | **CHEMBL1984236** |
| Cluster 6# 1 of 14 | 24 | 0.64 | -0.86 | 14 | 193.18 | 82.75 | 2 | 6 | 3 | 0 | 0.89 | CHEMBL1617690 |
| Cluster 6# 2 of 14 | 25 | 0.64 | -0.62 | 14 | 192.19 | 69.86 | 2 | 5 | 3 | 0 | 0.9 | CHEMBL1325899 |
| Cluster 6# 3 of 14 | 26 | 0.6 | -0.38 | 15 | 206.22 | 69.86 | 2 | 5 | 3 | 0 | 0.89 | CHEMBL1740773 |
| Cluster 6# 4 of 14 | 27 | 0.64 | -0.69 | 14 | 192.17 | 90.9 | 3 | 6 | 3 | 0 | 0.76 | CHEMBL1339020 |
| Cluster 6# 5 of 14 | 28 | 0.6 | -1.96 | 15 | 209.16 | 113.76 | 2 | 8 | 3 | 0 | 0.86 | CHEMBL1363359 |
| Cluster 6# 6 of 14 | 29 | 0.56 | -1.88 | 16 | 224.18 | 139.26 | 3 | 9 | 3 | 0 | 0.92 | CHEMBL1978187 |
| Cluster 6# 7 of 14 | 30 | 0.6 | -1.67 | 15 | 208.17 | 100.87 | 2 | 7 | 3 | 0 | 0.89 | CHEMBL1992624 |
| Cluster 6# 8 of 14 | 31 | 0.56 | 0.12 | 16 | 224.19 | 89.35 | 2 | 5 | 3 | 0 | 0.75 | CHEMBL571944 |
| Cluster 6# 9 of 14 | 32 | 0.64 | 0.82 | 14 | 192.21 | 58.14 | 2 | 4 | 3 | 0 | 0.78 | CHEMBL188286 |
| Cluster 6# 10 of 14 | 33 | 0.56 | -1.11 | 16 | 222.2 | 100.87 | 2 | 7 | 3 | 0 | 0.85 | CHEMBL1992624 |
| Cluster 6# 11 of 14 | 34 | 0.56 | -2.06 | 16 | 223.19 | 126.37 | 3 | 8 | 3 | 0 | 0.87 | CHEMBL1410365 |
| Cluster 6# 12 of 14 | 35 | 0.6 | 1.23 | 15 | 206.24 | 58.14 | 2 | 4 | 3 | 0 | 0.88 | CHEMBL160276 |
| Cluster 6# 13 of 14 | 36 | 0.64 | -1.1 | 14 | 193.16 | 104.65 | 2 | 7 | 3 | 0 | 0.84 | CHEMBL2070910 |
| Cluster 6# 14 of 14 | 37 | 0.56 | -1.33 | 16 | 222.2 | 100.87 | 2 | 7 | 3 | 0 | 0.85 | CHEMBL1413281 |
| Cluster 7# 1 of 4 | 38 | 0.64 | -2.61 | 14 | 198.18 | 91.48 | 2 | 7 | 2 | 0 | 0.81 | CHEMBL2218668 |
| Cluster 7# 2 of 4 | 39 | 0.64 | -2.49 | 14 | 197.19 | 97.27 | 2 | 7 | 2 | 0 | 0.81 | CHEMBL2218669 |
| Cluster 7# 3 of 4 | 40 | 0.6 | -2.4 | 15 | 212.21 | 91.48 | 2 | 7 | 3 | 0 | 0.8 | CHEMBL233684 |
| Cluster 7# 4 of 4 | 41 | 0.6 | -1.7 | 15 | 210.23 | 84.38 | 2 | 6 | 2 | 0 | 0.79 | CHEMBL2058828 |
| Cluster 8# 1 of 20 | 42 | 0.53 | -1.32 | 17 | 238.24 | 104.29 | 3 | 7 | 5 | 0 | 0.84 | CHEMBL2079793 |
| Cluster 8# 2 of 20 | 43 | 0.53 | -1.2 | 17 | 237.26 | 110.08 | 3 | 7 | 5 | 0 | 0.83 | CHEMBL1288656 |
| Cluster 8# 3 of 20 | 44 | 0.53 | -0.9 | 17 | 236.27 | 97.19 | 3 | 6 | 5 | 0 | 0.82 | CHEMBL475189 |
| Cluster 8# 4 of 20 | 45 | 0.56 | -1.64 | 16 | 223.26 | 117.49 | 3 | 7 | 4 | 0 | 0.85 | CHEMBL2170455 |
| Cluster 8# 5 of 20 | 46 | 0.56 | -1.74 | 16 | 221.22 | 106.92 | 2 | 7 | 4 | 0 | 0.83 | CHEMBL322655 |
| Cluster 8# 6 of 20 | 47 | 0.56 | -0.26 | 16 | 222.24 | 84.06 | 2 | 6 | 4 | 0 | 0.85 | CHEMBL13159 |
| Cluster 8# 7 of 20 | 48 | 0.56 | -0.15 | 16 | 221.26 | 89.85 | 2 | 6 | 4 | 0 | 0.84 | CHEMBL1329862 |
| Cluster 8# 8 of 20 | 49 | 0.53 | -0.89 | 17 | 238.25 | 122.97 | 3 | 8 | 5 | 0 | 0.83 | CHEMBL358067 |
| Cluster 8# 9 of 20 | 50 | 0.56 | 0.04 | 16 | 221.26 | 71.17 | 2 | 5 | 4 | 0 | 0.83 | CHEMBL2164468 |
| Cluster 8# 10 of 20 | 51 | 0.56 | 0.34 | 16 | 220.27 | 76.96 | 2 | 5 | 4 | 0 | 0.78 | CHEMBL145067 |
| Cluster 8# 11 of 20 | 52 | 0.56 | -1.65 | 16 | 224.22 | 104.29 | 3 | 7 | 4 | 0 | 0.76 | CHEMBL284298 |
| Cluster 8# 12 of 20 | 53 | 0.56 | 0.16 | 16 | 222.25 | 102.74 | 2 | 7 | 4 | 0 | 0.85 | CHEMBL358067 |
| Cluster 8# 13 of 20 | 54 | 0.56 | -1.53 | 16 | 223.23 | 110.08 | 3 | 7 | 4 | 0 | 0.76 | CHEMBL1288656 |
| Cluster 8# 14 of 20 | 55 | 0.56 | -1.35 | 16 | 223.23 | 91.4 | 3 | 6 | 4 | 0 | 0.76 | CHEMBL79820 |
| Cluster 8# 15 of 20 | 56 | 0.56 | -1.23 | 16 | 222.24 | 97.19 | 3 | 6 | 4 | 0 | 0.76 | CHEMBL1975534 |
| Cluster 8# 16 of 20 | 57 | 0.56 | 0.11 | 16 | 221.26 | 89.85 | 2 | 6 | 4 | 0 | 0.83 | CHEMBL1242474 |
| Cluster 8# 17 of 20 | 58 | 0.56 | -0.13 | 16 | 240.28 | 84.06 | 2 | 6 | 4 | 0 | 0.82 | CHEMBL22101 |
| Cluster 8# 18 of 20 | 59 | 0.53 | 0.23 | 17 | 236.27 | 84.06 | 2 | 6 | 5 | 0 | 0.77 | CHEMBL13159 |
| Cluster 8# 19 of 20 | 60 | 0.53 | 0.53 | 17 | 235.28 | 71.17 | 2 | 5 | 5 | 0 | 0.78 | CHEMBL79820 |
| Cluster 8# 20 of 20 | 61 | 0.53 | -1.13 | 17 | 236.29 | 104.6 | 3 | 6 | 5 | 0 | 0.76 | CHEMBL1975534 |
| Cluster 9# 1 of 17 | 62 | 0.64 | -1.45 | 14 | 193.16 | 87.72 | 2 | 6 | 2 | 0 | 0.76 | CHEMBL1985963 |
| Cluster 9# 2 of 17 | 63 | 0.64 | -1.67 | 14 | 194.15 | 101.13 | 2 | 7 | 2 | 0 | 0.75 | CHEMBL3250922 |
| Cluster 9# 3 of 17 | 64 | 0.64 | -1.55 | 14 | 193.16 | 106.92 | 2 | 7 | 2 | 0 | 0.75 | CHEMBL1801630 |
| Cluster 9# 4 of 17 | 65 | 0.69 | -0.6 | 13 | 180.18 | 71.9 | 3 | 5 | 2 | 0 | 0.73 | CHEMBL116125 |
| Cluster 9# 5 of 17 | 66 | 0.64 | -0.89 | 14 | 193.16 | 99.1 | 3 | 6 | 2 | 0 | 0.76 | CHEMBL2311659 |
| Cluster 9# 6 of 17 | 67 | 0.69 | -0.76 | 13 | 178.19 | 76.44 | 2 | 5 | 2 | 0 | 0.73 | CHEMBL297006 |
| Cluster 9# 7 of 17 | 68 | 0.69 | -0.49 | 13 | 179.2 | 77.69 | 3 | 5 | 2 | 0 | 0.73 | CHEMBL112383 |
| Cluster 9# 8 of 17 | 69 | 0.6 | -1.94 | 15 | 210.19 | 122.97 | 3 | 8 | 3 | 0 | 0.74 | CHEMBL358067 |
| Cluster 9# 9 of 17 | 70 | 0.64 | -0.62 | 14 | 209.23 | 89.1 | 2 | 5 | 2 | 0 | 0.73 | CHEMBL2047876 |
| Cluster 9# 10 of 17 | 71 | 0.69 | -0.24 | 13 | 179.17 | 66.49 | 2 | 4 | 2 | 0 | 0.73 | CHEMBL138468 |
| Cluster 9# 11 of 17 | 72 | 0.69 | -0.13 | 13 | 178.19 | 72.28 | 2 | 4 | 2 | 0 | 0.73 | CHEMBL2408615 |
| Cluster 9# 12 of 17 | 73 | 0.64 | -1.49 | 14 | 193.16 | 106.4 | 2 | 7 | 2 | 0 | 0.75 | CHEMBL454084 |
| Cluster 9# 13 of 17 | 74 | 0.69 | -0.49 | 13 | 179.18 | 89.33 | 2 | 6 | 2 | 0 | 0.73 | CHEMBL34625 |
| Cluster 9# 14 of 17 | 75 | 0.64 | -1.35 | 14 | 195.18 | 109.56 | 3 | 7 | 2 | 0 | 0.74 | CHEMBL513442 |
| Cluster 9# 15 of 17 | 76 | 0.64 | -0.97 | 14 | 211.2 | 96.2 | 2 | 6 | 2 | 0 | 0.74 | CHEMBL1341626 |
| Cluster 9# 16 of 17 | 77 | 0.64 | -1.61 | 14 | 194.15 | 100.61 | 2 | 7 | 2 | 0 | 0.75 | CHEMBL573553 |
| Cluster 9# 17 of 17 | 78 | 0.64 | -1.35 | 14 | 195.18 | 109.56 | 3 | 7 | 2 | 0 | 0.74 | CHEMBL513442 |
| Cluster 10# 1 of 4 | 79 | 0.67 | 0.62 | 15 | 205.21 | 62.58 | 2 | 4 | 3 | 0 | 0.72 | CHEMBL433105 |
| Cluster 10# 2 of 4 | 80 | 0.67 | 0.74 | 15 | 204.23 | 68.37 | 2 | 4 | 3 | 0 | 0.72 | CHEMBL577958 |
| Cluster 10# 3 of 4 | 81 | 0.62 | 0.69 | 16 | 219.24 | 73.58 | 3 | 4 | 3 | 0 | 0.68 | CHEMBL1322218 |
| Cluster 10# 4 of 4 | 82 | 0.62 | 0.41 | 16 | 219.24 | 81.26 | 2 | 5 | 3 | 0 | 0.7 | CHEMBL344386 |
| Cluster 11# 1 of 1 | 83 | 0.67 | -0.2 | 15 | 207.23 | 58.56 | 2 | 4 | 3 | 0 | 0.87 | CHEMBL1311149 |
| Cluster 12# 1 of 14 | 84 | 0.6 | -0.09 | 15 | 208.22 | 102.74 | 2 | 7 | 4 | 0 | 0.81 | CHEMBL131066 |
| Cluster 12# 2 of 14 | 85 | 0.6 | -0.14 | 15 | 207.23 | 89.85 | 2 | 6 | 4 | 0 | 0.82 | CHEMBL1970904 |
| Cluster 12# 3 of 14 | 86 | 0.56 | -0.77 | 16 | 220.23 | 84.06 | 2 | 6 | 4 | 0 | 0.77 | CHEMBL24102 |
| Cluster 12# 4 of 14 | 87 | 0.56 | -0.26 | 16 | 222.24 | 84.06 | 2 | 6 | 4 | 0 | 0.8 | CHEMBL280531 |
| Cluster 12# 5 of 14 | 88 | 0.56 | -0.15 | 16 | 221.26 | 89.85 | 2 | 6 | 4 | 0 | 0.79 | CHEMBL351296 |
| Cluster 12# 6 of 14 | 89 | 0.56 | 0.15 | 16 | 220.27 | 76.96 | 2 | 5 | 4 | 0 | 0.8 | CHEMBL474237 |
| Cluster 12# 7 of 14 | 90 | 0.53 | -1.24 | 17 | 235.24 | 92.93 | 2 | 7 | 5 | 0 | 0.82 | CHEMBL1972078 |
| Cluster 12# 8 of 14 | 91 | 0.6 | -0.76 | 15 | 208.22 | 86.19 | 2 | 6 | 4 | 0 | 0.72 | CHEMBL287172 |
| Cluster 12# 9 of 14 | 92 | 0.6 | -0.87 | 15 | 209.2 | 80.4 | 2 | 6 | 4 | 0 | 0.72 | CHEMBL305300 |
| Cluster 12# 10 of 14 | 93 | 0.56 | -0.27 | 16 | 222.24 | 86.19 | 2 | 6 | 4 | 0 | 0.77 | CHEMBL133217 |
| Cluster 12# 11 of 14 | 94 | 0.53 | 0.35 | 17 | 235.29 | 75.86 | 2 | 6 | 5 | 0 | 0.83 | CHEMBL1329862 |
| Cluster 12# 12 of 14 | 95 | 0.56 | -0.62 | 16 | 224.22 | 93.29 | 2 | 7 | 4 | 0 | 0.72 | CHEMBL38324 |
| Cluster 12# 13 of 14 | 96 | 0.53 | -1.38 | 17 | 236.23 | 125.12 | 4 | 7 | 4 | 0 | 0.81 | CHEMBL124428 |
| Cluster 12# 14 of 14 | 97 | 0.56 | 0.11 | 16 | 221.26 | 89.85 | 2 | 6 | 4 | 0 | 0.8 | CHEMBL1242381 |
| Cluster 13# 1 of 9 | 98 | 0.6 | -0.63 | 15 | 206.2 | 75.35 | 2 | 5 | 2 | 0 | 0.69 | CHEMBL3187933 |
| Cluster 13# 2 of 9 | 99 | 0.64 | -0.76 | 14 | 192.17 | 104.89 | 3 | 6 | 2 | 0 | 0.75 | CHEMBL534356 |
| Cluster 13# 3 of 9 | 100 | 0.64 | -0.59 | 14 | 209.23 | 89.1 | 2 | 5 | 2 | 0 | 0.72 | CHEMBL549673 |
| Cluster 13# 4 of 9 | 101 | 0.6 | -1.24 | 15 | 208.19 | 88.97 | 3 | 6 | 2 | 0 | 0.76 | CHEMBL21030 |
| Cluster 13# 5 of 9 | 102 | 0.64 | -0.91 | 14 | 194.15 | 111.99 | 3 | 7 | 2 | 0 | 0.75 | CHEMBL213788 |
| Cluster 13# 6 of 9 | 103 | 0.64 | -0.44 | 14 | 210.21 | 83.31 | 2 | 5 | 2 | 0 | 0.7 | CHEMBL73774 |
| Cluster 13# 7 of 9 | 104 | 0.6 | -0.86 | 15 | 205.21 | 81.14 | 2 | 5 | 2 | 0 | 0.69 | CHEMBL3234419 |
| Cluster 13# 8 of 9 | 105 | 0.64 | -0.51 | 14 | 192.17 | 104.89 | 3 | 6 | 2 | 0 | 0.76 | CHEMBL2315148 |
| Cluster 13# 9 of 9 | 106 | 0.6 | -1.16 | 15 | 207.19 | 130.91 | 4 | 7 | 2 | 0 | 0.76 | CHEMBL534356 |
| Cluster 14# 1 of 5 | 107 | 0.6 | -0.13 | 15 | 207.23 | 81.17 | 4 | 5 | 4 | 0 | 0.84 | CHEMBL559742 |
| Cluster 14# 2 of 5 | 108 | 0.56 | 0.17 | 16 | 221.26 | 81.17 | 4 | 5 | 4 | 0 | 0.8 | CHEMBL559942 |
| Cluster 14# 3 of 5 | 109 | 0.6 | -1.22 | 15 | 207.19 | 103.79 | 3 | 7 | 4 | 0 | 0.85 | CHEMBL436096 |
| Cluster 14# 4 of 5 | 110 | 0.6 | -0.9 | 15 | 226.26 | 94.92 | 3 | 6 | 4 | 0 | 0.64 | CHEMBL469245 |
| Cluster 14# 5 of 5 | 111 | 0.6 | -0.14 | 15 | 206.2 | 75.21 | 2 | 5 | 4 | 0 | 0.74 | CHEMBL572089 |
| Cluster 15# 1 of 1 | 112 | 0.79 | -0.84 | 14 | 216.26 | 84.5 | 2 | 6 | 2 | 0 | 0.71 | CHEMBL1353173 |
| Cluster 16# 1 of 9 | 113 | 0.67 | 0.82 | 15 | 228.67 | 60.69 | 3 | 3 | 1 | 0 | 0.74 | CHEMBL290225 |
| Cluster 16# 2 of 9 | 114 | 0.67 | 0.34 | 15 | 212.22 | 60.69 | 3 | 3 | 1 | 0 | 0.74 | CHEMBL432676 |
| Cluster 16# 3 of 9 | 115 | 0.71 | 0.2 | 14 | 194.23 | 60.69 | 3 | 3 | 1 | 0 | 0.73 | CHEMBL26736 |
| Cluster 16# 4 of 9 | 116 | 0.71 | -0.98 | 14 | 196.22 | 77.3 | 4 | 4 | 1 | 0 | 0.73 | CHEMBL272676 |
| Cluster 16# 5 of 9 | 117 | 0.67 | 0.12 | 15 | 210.23 | 58.92 | 2 | 4 | 2 | 0 | 0.7 | CHEMBL1554844 |
| Cluster 16# 6 of 9 | 118 | 0.62 | 0.28 | 16 | 224.25 | 69.92 | 3 | 4 | 2 | 0 | 0.8 | CHEMBL2380480 |
| Cluster 16# 7 of 9 | 119 | 0.67 | -0.36 | 15 | 230.67 | 77.3 | 4 | 4 | 1 | 0 | 0.72 | CHEMBL408295 |
| Cluster 16# 8 of 9 | 120 | 0.67 | 0.46 | 15 | 208.25 | 60.69 | 3 | 3 | 1 | 0 | 0.71 | CHEMBL464458 |
| Cluster 16# 9 of 9 | 121 | 0.62 | 0.28 | 16 | 224.25 | 69.92 | 3 | 4 | 2 | 0 | 0.74 | CHEMBL2442728 |
| Cluster 17# 1 of 2 | 122 | 0.67 | -0.77 | 15 | 209.24 | 92.26 | 3 | 5 | 2 | 0 | 0.85 | CHEMBL22567 |
| Cluster 17# 2 of 2 | 123 | 0.67 | -0.66 | 15 | 208.26 | 98.05 | 3 | 5 | 2 | 0 | 0.85 | CHEMBL6696 |
| Cluster 18# 1 of 2 | 124 | 0.64 | -0.3 | 14 | 194.19 | 85.44 | 2 | 5 | 2 | 0 | 0.84 | CHEMBL3128260 |
| Cluster 18# 2 of 2 | 125 | 0.6 | 0.09 | 15 | 208.21 | 85.44 | 2 | 5 | 2 | 0 | 0.83 | CHEMBL3128260 |
| Cluster 19# 1 of 1 | 126 | 0.62 | -0.58 | 16 | 224.28 | 60.69 | 3 | 4 | 2 | 0 | 0.8 | CHEMBL2441670 |
| Cluster 20# 1 of 1 | 127 | 0.53 | 1.18 | 17 | 236.27 | 83.54 | 2 | 6 | 4 | 0 | 0.75 | CHEMBL370962 |
| Cluster 21# 1 of 2 | 128 | 0.56 | -0.25 | 16 | 221.26 | 79.68 | 2 | 6 | 4 | 0 | 0.79 | CHEMBL318652 |
| Cluster 21# 2 of 2 | 129 | 0.53 | 0.24 | 17 | 235.29 | 79.68 | 2 | 6 | 5 | 0 | 0.75 | CHEMBL327285 |
| Cluster 22# 1 of 1 | 130 | 0.56 | -2.45 | 16 | 225.2 | 130.05 | 4 | 8 | 3 | 0 | 0.86 | CHEMBL1980081 |
| Cluster 23# 1 of 1 | 131 | 0.56 | 0.76 | 16 | 219.22 | 78.18 | 1 | 5 | 3 | 0 | 0.68 | CHEMBL1456512 |
| Cluster 24# 1 of 2 | 132 | 0.53 | -1.01 | 17 | 237.26 | 99.08 | 2 | 7 | 5 | 0 | 0.75 | CHEMBL3250899 |
| Cluster 24# 2 of 2 | 133 | 0.53 | -1.24 | 17 | 236.27 | 97.19 | 3 | 6 | 5 | 0 | 0.75 | CHEMBL474237 |
| Cluster 25# 1 of 1 | 134 | 0.67 | -2.59 | 15 | 215.23 | 106.7 | 5 | 7 | 3 | 0 | 0.73 | CHEMBL480173 |
| Cluster 26# 1 of 1 | 135 | 0.53 | 0.45 | 17 | 254.72 | 62.97 | 2 | 5 | 5 | 0 | 0.85 | CHEMBL401038 |
| Cluster 27# 1 of 1 | 136 | 0.75 | -0.18 | 12 | 165.19 | 53.35 | 2 | 3 | 2 | 0 | 0.79 | CHEMBL226002 |
| Cluster 28# 1 of 6 | 137 | 0.43 | -0.15 | 14 | 216.62 | 77.76 | 3 | 4 | 3 | 0 | 0.88 | CHEMBL28533 |
| Cluster 28# 2 of 6 | 138 | 0.43 | -0.79 | 14 | 198.22 | 80.92 | 4 | 4 | 4 | 0 | 0.75 | CHEMBL469847 |
| Cluster 28# 3 of 6 | 139 | 0.43 | -1.18 | 14 | 197.19 | 89.79 | 4 | 5 | 4 | 0 | 0.76 | CHEMBL386111 |
| Cluster 28# 4 of 6 | 140 | 0.43 | -0.23 | 14 | 198.22 | 69.92 | 3 | 4 | 4 | 0 | 0.73 | CHEMBL376901 |
| Cluster 28# 5 of 6 | 141 | 0.4 | -2.61 | 15 | 213.19 | 107.32 | 2 | 7 | 3 | 0 | 0.82 | CHEMBL1465364 |
| Cluster 28# 6 of 6 | 142 | 0.43 | -0.38 | 14 | 215.27 | 92.26 | 3 | 5 | 4 | 0 | 0.83 | CHEMBL1733134 |
| Cluster 29# 1 of 31 | 143 | 0.38 | -0.8 | 13 | 183.18 | 78.18 | 1 | 5 | 4 | 0 | 0.8 | CHEMBL1933846 |
| Cluster 29# 2 of 31 | 144 | 0.42 | -0.79 | 12 | 170.14 | 86.39 | 1 | 5 | 4 | 0 | 0.78 | CHEMBL1374330 |
| Cluster 29# 3 of 31 | 145 | 0.42 | -0.75 | 12 | 186.23 | 76.21 | 2 | 4 | 4 | 0 | 0.77 | CHEMBL583613 |
| Cluster 29# 4 of 31 | 146 | 0.42 | -0.96 | 12 | 169.16 | 89.04 | 2 | 5 | 4 | 0 | 0.77 | CHEMBL1933846 |
| Cluster 29# 5 of 31 | 147 | 0.38 | -1.24 | 13 | 183.21 | 81.14 | 2 | 5 | 4 | 0 | 0.76 | CHEMBL1770760 |
| Cluster 29# 6 of 31 | 148 | 0.42 | -1.51 | 12 | 171.15 | 102.24 | 2 | 6 | 4 | 0 | 0.77 | CHEMBL226958 |
| Cluster 29# 7 of 31 | 149 | 0.36 | 0.08 | 14 | 212.27 | 76.21 | 2 | 4 | 5 | 0 | 0.76 | CHEMBL2260634 |
| Cluster 29# 8 of 31 | 150 | 0.42 | -0.77 | 12 | 187.22 | 89.1 | 2 | 5 | 4 | 0 | 0.77 | CHEMBL1650287 |
| Cluster 29# 9 of 31 | 151 | 0.38 | -0.23 | 13 | 181.17 | 78.18 | 1 | 5 | 3 | 0 | 0.7 | CHEMBL1933840 |
| Cluster 29# 10 of 31 | 152 | 0.38 | -0.66 | 13 | 181.19 | 81.14 | 2 | 5 | 3 | 0 | 0.69 | CHEMBL113953 |
| Cluster 29# 11 of 31 | 153 | 0.42 | -0.81 | 12 | 167.17 | 92 | 3 | 5 | 3 | 0 | 0.69 | CHEMBL113953 |
| Cluster 29# 12 of 31 | 154 | 0.42 | -0.39 | 12 | 167.14 | 89.04 | 2 | 5 | 3 | 0 | 0.69 | CHEMBL1933840 |
| Cluster 29# 13 of 31 | 155 | 0.42 | -0.18 | 12 | 184.22 | 76.21 | 2 | 4 | 3 | 0 | 0.69 | CHEMBL582990 |
| Cluster 29# 14 of 31 | 156 | 0.38 | -2.05 | 13 | 185.18 | 120.05 | 3 | 7 | 4 | 0 | 0.82 | CHEMBL1988822 |
| Cluster 29# 15 of 31 | 157 | 0.42 | -1.92 | 12 | 173.17 | 91.4 | 3 | 6 | 4 | 0 | 0.73 | CHEMBL1289540 |
| Cluster 29# 16 of 31 | 158 | 0.38 | -1.81 | 13 | 185.18 | 98.24 | 4 | 6 | 5 | 0 | 0.71 | CHEMBL1906612 |
| Cluster 29# 17 of 31 | 159 | 0.38 | -0.21 | 13 | 200.26 | 65.98 | 2 | 4 | 4 | 0 | 0.79 | CHEMBL1888272 |
| Cluster 29# 18 of 31 | 160 | 0.42 | -0.45 | 12 | 186.23 | 65.98 | 2 | 4 | 4 | 0 | 0.78 | CHEMBL1888272 |
| Cluster 29# 19 of 31 | 161 | 0.42 | -2.28 | 12 | 171.16 | 106.92 | 2 | 7 | 4 | 0 | 0.76 | CHEMBL1376407 |
| Cluster 29# 20 of 31 | 162 | 0.42 | -1.03 | 12 | 187.22 | 88.24 | 3 | 5 | 4 | 0 | 0.71 | CHEMBL200920 |
| Cluster 29# 21 of 31 | 163 | 0.38 | -1.09 | 13 | 185.18 | 102.24 | 2 | 6 | 4 | 0 | 0.8 | CHEMBL226958 |
| Cluster 29# 22 of 31 | 164 | 0.38 | -1.55 | 13 | 186.17 | 84.58 | 2 | 6 | 4 | 0 | 0.78 | CHEMBL283733 |
| Cluster 29# 23 of 31 | 165 | 0.42 | -1.24 | 12 | 188.21 | 104.89 | 3 | 6 | 4 | 0 | 0.73 | CHEMBL1490950 |
| Cluster 29# 24 of 31 | 166 | 0.38 | -0.66 | 13 | 182.18 | 86.32 | 3 | 5 | 5 | 0 | 0.69 | CHEMBL120918 |
| Cluster 29# 25 of 31 | 167 | 0.38 | -0.97 | 13 | 183.16 | 99.21 | 3 | 6 | 5 | 0 | 0.7 | CHEMBL207590 |
| Cluster 29# 26 of 31 | 168 | 0.38 | -0.66 | 13 | 183.18 | 89.04 | 2 | 5 | 4 | 0 | 0.81 | CHEMBL1540603 |
| Cluster 29# 27 of 31 | 169 | 0.38 | -1.52 | 13 | 183.17 | 114.75 | 4 | 7 | 5 | 0 | 0.73 | CHEMBL207966 |
| Cluster 29# 28 of 31 | 170 | 0.38 | -1.01 | 13 | 184.15 | 112.1 | 3 | 7 | 5 | 0 | 0.71 | CHEMBL206473 |
| Cluster 29# 29 of 31 | 171 | 0.38 | -1.47 | 13 | 182.18 | 101.86 | 4 | 6 | 5 | 0 | 0.73 | CHEMBL207813 |
| Cluster 29# 30 of 31 | 172 | 0.38 | -0.31 | 13 | 185.18 | 101.23 | 3 | 6 | 4 | 0 | 0.74 | CHEMBL1540752 |
| Cluster 29# 31 of 31 | 173 | 0.38 | -0.31 | 13 | 185.18 | 101.23 | 3 | 6 | 4 | 0 | 0.7 | CHEMBL1540752 |
| Cluster 30# 1 of 1 | 174 | 0.53 | -0.65 | 15 | 229.26 | 88.24 | 2 | 6 | 5 | 0 | 0.7 | CHEMBL1571313 |
| Cluster 31# 1 of 55 | 175 | 0.46 | -1.58 | 13 | 185.18 | 101.49 | 3 | 6 | 4 | 0 | 0.83 | CHEMBL2337923 |
| Cluster 31# 2 of 55 | 176 | 0.46 | -0.76 | 13 | 183.16 | 79.65 | 2 | 5 | 4 | 0 | 0.76 | CHEMBL1797391 |
| Cluster 31# 3 of 55 | 177 | 0.46 | -1.11 | 13 | 182.18 | 82.45 | 3 | 5 | 4 | 0 | 0.77 | CHEMBL2286652 |
| Cluster 31# 4 of 55 | 178 | 0.46 | -0.99 | 13 | 181.19 | 88.24 | 3 | 5 | 4 | 0 | 0.77 | CHEMBL44868 |
| Cluster 31# 5 of 55 | 179 | 0.43 | -0.49 | 14 | 216.62 | 82.45 | 3 | 5 | 4 | 0 | 0.76 | CHEMBL357098 |
| Cluster 31# 6 of 55 | 180 | 0.4 | -1 | 15 | 211.21 | 89.79 | 4 | 5 | 4 | 0 | 0.75 | CHEMBL190452 |
| Cluster 31# 7 of 55 | 181 | 0.43 | -0.58 | 14 | 198.24 | 88.33 | 4 | 4 | 4 | 0 | 0.8 | CHEMBL1302256 |
| Cluster 31# 8 of 55 | 182 | 0.5 | -0.17 | 12 | 168.19 | 65.38 | 3 | 4 | 4 | 0 | 0.8 | CHEMBL1088766 |
| Cluster 31# 9 of 55 | 183 | 0.5 | -0.06 | 12 | 167.21 | 71.17 | 3 | 4 | 4 | 0 | 0.8 | CHEMBL189125 |
| Cluster 31# 10 of 55 | 184 | 0.43 | -0.48 | 14 | 198.22 | 80.92 | 4 | 4 | 4 | 0 | 0.75 | CHEMBL1778727 |
| Cluster 31# 11 of 55 | 185 | 0.4 | 0.54 | 15 | 212.22 | 60.69 | 3 | 3 | 4 | 0 | 0.76 | CHEMBL2323720 |
| Cluster 31# 12 of 55 | 186 | 0.46 | -1.1 | 13 | 182.18 | 82.45 | 3 | 5 | 4 | 0 | 0.76 | CHEMBL2385132 |
| Cluster 31# 13 of 55 | 187 | 0.46 | -1.83 | 13 | 182.18 | 101.13 | 3 | 6 | 4 | 0 | 0.77 | CHEMBL18873 |
| Cluster 31# 14 of 55 | 188 | 0.4 | -0.39 | 15 | 210.23 | 77.76 | 3 | 4 | 4 | 0 | 0.77 | CHEMBL2392186 |
| Cluster 31# 15 of 55 | 189 | 0.4 | -0.55 | 15 | 214.19 | 77.76 | 3 | 4 | 4 | 0 | 0.77 | CHEMBL2323720 |
| Cluster 31# 16 of 55 | 190 | 0.5 | -0.67 | 12 | 169.18 | 81.26 | 2 | 5 | 4 | 0 | 0.79 | CHEMBL2337924 |
| Cluster 31# 17 of 55 | 191 | 0.46 | -1.85 | 13 | 182.18 | 101.13 | 3 | 6 | 4 | 0 | 0.76 | CHEMBL135674 |
| Cluster 31# 18 of 55 | 192 | 0.46 | -1.14 | 13 | 200.22 | 83.31 | 2 | 5 | 4 | 0 | 0.79 | CHEMBL1361871 |
| Cluster 31# 19 of 55 | 193 | 0.46 | -1.03 | 13 | 199.23 | 89.1 | 2 | 5 | 4 | 0 | 0.79 | CHEMBL491056 |
| Cluster 31# 20 of 55 | 194 | 0.43 | -1.34 | 14 | 195.22 | 89.1 | 2 | 5 | 4 | 0 | 0.82 | CHEMBL1300438 |
| Cluster 31# 21 of 55 | 195 | 0.46 | -0.96 | 13 | 181.22 | 96.76 | 4 | 5 | 4 | 0 | 0.77 | CHEMBL425180 |
| Cluster 31# 22 of 55 | 196 | 0.5 | -1.04 | 12 | 169.18 | 78.27 | 3 | 5 | 4 | 0 | 0.78 | CHEMBL1326975 |
| Cluster 31# 23 of 55 | 197 | 0.46 | -2.09 | 13 | 183.17 | 114.02 | 3 | 7 | 4 | 0 | 0.85 | CHEMBL1707118 |
| Cluster 31# 24 of 55 | 198 | 0.46 | -2.2 | 13 | 184.15 | 108.23 | 3 | 7 | 4 | 0 | 0.85 | CHEMBL2238372 |
| Cluster 31# 25 of 55 | 199 | 0.46 | -1.58 | 13 | 181.19 | 89.1 | 2 | 5 | 4 | 0 | 0.78 | CHEMBL1300438 |
| Cluster 31# 26 of 55 | 200 | 0.46 | -1.4 | 13 | 182.18 | 83.05 | 2 | 5 | 4 | 0 | 0.84 | CHEMBL140077 |
| Cluster 31# 27 of 55 | 201 | 0.46 | -0.21 | 13 | 184.22 | 72.25 | 2 | 5 | 4 | 0 | 0.77 | CHEMBL34717 |
| Cluster 31# 28 of 55 | 202 | 0.46 | -0.38 | 13 | 200.26 | 84.92 | 2 | 5 | 4 | 0 | 0.81 | CHEMBL3104282 |
| Cluster 31# 29 of 55 | 203 | 0.5 | -0.84 | 12 | 170.17 | 91.16 | 3 | 6 | 4 | 0 | 0.79 | CHEMBL1585263 |
| Cluster 31# 30 of 55 | 204 | 0.46 | -1.32 | 13 | 183.16 | 79.65 | 2 | 5 | 4 | 0 | 0.7 | CHEMBL2048818 |
| Cluster 31# 31 of 55 | 205 | 0.46 | -1.93 | 13 | 183.16 | 96.2 | 2 | 6 | 4 | 0 | 0.79 | CHEMBL1345200 |
| Cluster 31# 32 of 55 | 206 | 0.46 | -0.72 | 13 | 180.2 | 76.21 | 2 | 4 | 4 | 0 | 0.74 | CHEMBL109484 |
| Cluster 31# 33 of 55 | 207 | 0.46 | -0.83 | 13 | 181.19 | 70.42 | 2 | 4 | 4 | 0 | 0.74 | CHEMBL9208 |
| Cluster 31# 34 of 55 | 208 | 0.46 | -1.8 | 13 | 182.2 | 109.65 | 4 | 6 | 4 | 0 | 0.77 | CHEMBL370264 |
| Cluster 31# 35 of 55 | 209 | 0.46 | -1.77 | 13 | 184.15 | 108.23 | 3 | 7 | 4 | 0 | 0.78 | CHEMBL1411824 |
| Cluster 31# 36 of 55 | 210 | 0.4 | -1.85 | 15 | 211.22 | 109.33 | 3 | 6 | 4 | 0 | 0.86 | CHEMBL1300438 |
| Cluster 31# 37 of 55 | 211 | 0.46 | -0.9 | 13 | 183.21 | 81.26 | 2 | 5 | 4 | 0 | 0.81 | CHEMBL581298 |
| Cluster 31# 38 of 55 | 212 | 0.5 | -0.21 | 12 | 167.21 | 72.03 | 2 | 4 | 4 | 0 | 0.71 | CHEMBL1783711 |
| Cluster 31# 39 of 55 | 213 | 0.43 | -1.58 | 14 | 200.2 | 103.26 | 2 | 7 | 4 | 0 | 0.81 | CHEMBL38261 |
| Cluster 31# 40 of 55 | 214 | 0.43 | -0.8 | 14 | 193.2 | 86.07 | 3 | 5 | 5 | 0 | 0.72 | CHEMBL131190 |
| Cluster 31# 41 of 55 | 215 | 0.46 | -1.68 | 13 | 184.2 | 104.29 | 4 | 6 | 4 | 0 | 0.7 | CHEMBL1971024 |
| Cluster 31# 42 of 55 | 216 | 0.46 | -0.21 | 13 | 184.22 | 72.25 | 2 | 5 | 4 | 0 | 0.78 | CHEMBL34717 |
| Cluster 31# 43 of 55 | 217 | 0.5 | -0.58 | 12 | 170.17 | 109.84 | 3 | 7 | 4 | 0 | 0.79 | CHEMBL1210571 |
| Cluster 31# 44 of 55 | 218 | 0.46 | -1.55 | 13 | 185.18 | 98.5 | 4 | 6 | 4 | 0 | 0.8 | CHEMBL1326975 |
| Cluster 31# 45 of 55 | 219 | 0.46 | -1.43 | 13 | 184.2 | 104.29 | 4 | 6 | 4 | 0 | 0.8 | CHEMBL35281 |
| Cluster 31# 46 of 55 | 220 | 0.46 | -1.07 | 13 | 200.22 | 83.31 | 2 | 5 | 4 | 0 | 0.65 | CHEMBL1863689 |
| Cluster 31# 47 of 55 | 221 | 0.46 | -0.96 | 13 | 199.23 | 89.1 | 2 | 5 | 4 | 0 | 0.65 | CHEMBL491056 |
| Cluster 31# 48 of 55 | 222 | 0.43 | -2.09 | 14 | 197.19 | 109.33 | 3 | 6 | 4 | 0 | 0.83 | CHEMBL1300438 |
| Cluster 31# 49 of 55 | 223 | 0.46 | -0.83 | 13 | 180.16 | 83.05 | 2 | 5 | 3 | 0 | 0.8 | CHEMBL270196 |
| Cluster 31# 50 of 55 | 224 | 0.43 | -0.96 | 14 | 199.21 | 90.37 | 2 | 6 | 4 | 0 | 0.77 | CHEMBL37808 |
| Cluster 31# 51 of 55 | 225 | 0.46 | -1.14 | 13 | 183.23 | 88.64 | 3 | 5 | 4 | 0 | 0.77 | CHEMBL581318 |
| Cluster 31# 52 of 55 | 226 | 0.46 | -1.58 | 13 | 185.18 | 101.49 | 3 | 6 | 4 | 0 | 0.72 | CHEMBL2337924 |
| Cluster 31# 53 of 55 | 227 | 0.46 | -0.83 | 13 | 180.16 | 83.05 | 2 | 5 | 3 | 0 | 0.69 | CHEMBL236647 |
| Cluster 31# 54 of 55 | 228 | 0.46 | -1.43 | 13 | 183.21 | 92.26 | 3 | 5 | 4 | 0 | 0.61 | CHEMBL387509 |
| Cluster 31# 55 of 55 | 229 | 0.46 | -1.25 | 13 | 180.16 | 83.31 | 2 | 5 | 3 | 0 | 0.66 | CHEMBL439803 |
| Cluster 32# 1 of 12 | 230 | 0.5 | 0.45 | 12 | 168.19 | 49.69 | 2 | 3 | 3 | 0 | 0.84 | CHEMBL2270992 |
| Cluster 32# 2 of 12 | 231 | 0.5 | 0.77 | 12 | 188.61 | 49.69 | 2 | 3 | 3 | 0 | 0.83 | CHEMBL3183824 |
| Cluster 32# 3 of 12 | 232 | 0.46 | 0.59 | 13 | 186.18 | 49.69 | 2 | 3 | 3 | 0 | 0.83 | CHEMBL35729 |
| Cluster 32# 4 of 12 | 233 | 0.46 | 0.86 | 13 | 181.23 | 55.48 | 2 | 3 | 3 | 0 | 0.83 | CHEMBL325689 |
| Cluster 32# 5 of 12 | 234 | 0.46 | 0.23 | 13 | 184.19 | 58.92 | 2 | 4 | 4 | 0 | 0.81 | CHEMBL3244455 |
| Cluster 32# 6 of 12 | 235 | 0.46 | 0.81 | 13 | 182.22 | 49.69 | 2 | 3 | 4 | 0 | 0.81 | CHEMBL1091561 |
| Cluster 32# 7 of 12 | 236 | 0.46 | 1.04 | 13 | 180.2 | 49.69 | 2 | 3 | 4 | 0 | 0.8 | CHEMBL14307 |
| Cluster 32# 8 of 12 | 237 | 0.5 | -0.51 | 12 | 170.16 | 69.92 | 3 | 4 | 3 | 0 | 0.84 | CHEMBL467758 |
| Cluster 32# 9 of 12 | 238 | 0.5 | -0.62 | 12 | 169.18 | 75.71 | 3 | 4 | 3 | 0 | 0.84 | CHEMBL1347898 |
| Cluster 32# 10 of 12 | 239 | 0.43 | -0.94 | 14 | 202.2 | 88.33 | 4 | 4 | 3 | 0 | 0.75 | CHEMBL2105069 |
| Cluster 32# 11 of 12 | 240 | 0.46 | -0.08 | 13 | 179.17 | 73.48 | 2 | 4 | 3 | 0 | 0.79 | CHEMBL1321809 |
| Cluster 32# 12 of 12 | 241 | 0.46 | 0.04 | 13 | 178.19 | 79.27 | 2 | 4 | 3 | 0 | 0.79 | CHEMBL7853 |
| Cluster 33# 1 of 14 | 242 | 0.43 | -0.13 | 14 | 196.2 | 77.76 | 3 | 4 | 3 | 0 | 0.81 | CHEMBL1504659 |
| Cluster 33# 2 of 14 | 243 | 0.46 | -0.54 | 13 | 181.19 | 83.55 | 3 | 4 | 3 | 0 | 0.81 | CHEMBL1340054 |
| Cluster 33# 3 of 14 | 244 | 0.46 | -0.43 | 13 | 182.17 | 77.76 | 3 | 4 | 3 | 0 | 0.81 | CHEMBL105609 |
| Cluster 33# 4 of 14 | 245 | 0.46 | -0.54 | 13 | 181.19 | 83.55 | 3 | 4 | 3 | 0 | 0.81 | CHEMBL3275148 |
| Cluster 33# 5 of 14 | 246 | 0.4 | 0.17 | 15 | 210.23 | 77.76 | 3 | 4 | 3 | 0 | 0.79 | CHEMBL448004 |
| Cluster 33# 6 of 14 | 247 | 0.43 | -0.13 | 14 | 196.2 | 77.76 | 3 | 4 | 3 | 0 | 0.78 | CHEMBL448004 |
| Cluster 33# 7 of 14 | 248 | 0.43 | -3.04 | 14 | 199.19 | 126.96 | 4 | 7 | 3 | 0 | 0.87 | CHEMBL503948 |
| Cluster 33# 8 of 14 | 249 | 0.43 | -0.19 | 14 | 196.25 | 69.72 | 3 | 4 | 4 | 0 | 0.75 | CHEMBL443560 |
| Cluster 33# 9 of 14 | 250 | 0.43 | -0.08 | 14 | 197.23 | 63.93 | 3 | 4 | 4 | 0 | 0.75 | CHEMBL159898 |
| Cluster 33# 10 of 14 | 251 | 0.43 | 0.21 | 14 | 195.22 | 72.55 | 2 | 4 | 4 | 0 | 0.8 | CHEMBL1340054 |
| Cluster 33# 11 of 14 | 252 | 0.46 | -0.24 | 13 | 181.19 | 84.54 | 4 | 4 | 3 | 0 | 0.81 | CHEMBL503157 |
| Cluster 33# 12 of 14 | 253 | 0.43 | 1.06 | 14 | 194.23 | 49.69 | 2 | 3 | 4 | 0 | 0.82 | CHEMBL445285 |
| Cluster 33# 13 of 14 | 254 | 0.4 | 0.61 | 15 | 210.23 | 66.76 | 2 | 4 | 4 | 0 | 0.8 | CHEMBL1504659 |
| Cluster 33# 14 of 14 | 255 | 0.46 | -1.16 | 13 | 181.19 | 89.1 | 2 | 5 | 3 | 0 | 0.84 | CHEMBL495577 |
| Cluster 34# 1 of 26 | 256 | 0.43 | 0.02 | 14 | 197.23 | 63.93 | 3 | 4 | 4 | 0 | 0.75 | CHEMBL350165 |
| Cluster 34# 2 of 26 | 257 | 0.43 | -0.1 | 14 | 202.18 | 69.92 | 3 | 4 | 4 | 0 | 0.83 | CHEMBL10494 |
| Cluster 34# 3 of 26 | 258 | 0.43 | 0.06 | 14 | 198.22 | 69.92 | 3 | 4 | 4 | 0 | 0.82 | CHEMBL46820 |
| Cluster 34# 4 of 26 | 259 | 0.43 | 0.27 | 14 | 217.65 | 75.71 | 3 | 4 | 4 | 0 | 0.82 | CHEMBL205950 |
| Cluster 34# 5 of 26 | 260 | 0.43 | 0.27 | 14 | 217.65 | 75.71 | 3 | 4 | 4 | 0 | 0.82 | CHEMBL1668128 |
| Cluster 34# 6 of 26 | 261 | 0.43 | -0.96 | 14 | 197.19 | 89.79 | 4 | 5 | 4 | 0 | 0.8 | CHEMBL1353352 |
| Cluster 34# 7 of 26 | 262 | 0.43 | -0.09 | 14 | 196.25 | 69.72 | 3 | 4 | 4 | 0 | 0.75 | CHEMBL1774331 |
| Cluster 34# 8 of 26 | 263 | 0.43 | -0.68 | 14 | 196.2 | 77.76 | 3 | 4 | 4 | 0 | 0.78 | CHEMBL3093470 |
| Cluster 34# 9 of 26 | 264 | 0.43 | -0.79 | 14 | 195.22 | 83.55 | 3 | 4 | 4 | 0 | 0.78 | CHEMBL135482 |
| Cluster 34# 10 of 26 | 265 | 0.43 | -0.79 | 14 | 195.22 | 83.55 | 3 | 4 | 4 | 0 | 0.78 | CHEMBL132543 |
| Cluster 34# 11 of 26 | 266 | 0.43 | 0.91 | 14 | 196.24 | 60.69 | 3 | 3 | 4 | 0 | 0.78 | CHEMBL261144 |
| Cluster 34# 12 of 26 | 267 | 0.43 | 0.65 | 14 | 212.27 | 60.69 | 3 | 3 | 4 | 0 | 0.75 | CHEMBL512867 |
| Cluster 34# 13 of 26 | 268 | 0.43 | 0.8 | 14 | 195.26 | 66.48 | 3 | 3 | 4 | 0 | 0.78 | CHEMBL251175 |
| Cluster 34# 14 of 26 | 269 | 0.46 | -0.76 | 13 | 183.16 | 79.65 | 2 | 5 | 4 | 0 | 0.79 | CHEMBL2139746 |
| Cluster 34# 15 of 26 | 270 | 0.46 | -0.65 | 13 | 182.18 | 85.44 | 2 | 5 | 4 | 0 | 0.79 | CHEMBL1302412 |
| Cluster 34# 16 of 26 | 271 | 0.43 | -0.27 | 14 | 196.2 | 77.76 | 3 | 4 | 4 | 0 | 0.71 | CHEMBL485875 |
| Cluster 34# 17 of 26 | 272 | 0.43 | -1.23 | 14 | 196.2 | 73.66 | 2 | 5 | 4 | 0 | 0.77 | CHEMBL205576 |
| Cluster 34# 18 of 26 | 273 | 0.43 | -0.1 | 14 | 197.23 | 78.27 | 3 | 5 | 4 | 0 | 0.78 | CHEMBL401295 |
| Cluster 34# 19 of 26 | 274 | 0.43 | -0.53 | 14 | 197.19 | 79.65 | 2 | 5 | 4 | 0 | 0.77 | CHEMBL1319725 |
| Cluster 34# 20 of 26 | 275 | 0.43 | -0.41 | 14 | 196.2 | 85.44 | 2 | 5 | 4 | 0 | 0.77 | CHEMBL1302412 |
| Cluster 34# 21 of 26 | 276 | 0.46 | -0.06 | 13 | 183.2 | 62.58 | 2 | 4 | 4 | 0 | 0.74 | CHEMBL3288361 |
| Cluster 34# 22 of 26 | 277 | 0.46 | 0.02 | 13 | 181.23 | 72.03 | 2 | 4 | 4 | 0 | 0.74 | CHEMBL1783711 |
| Cluster 34# 23 of 26 | 278 | 0.43 | -0.57 | 14 | 196.2 | 82.45 | 3 | 5 | 4 | 0 | 0.67 | CHEMBL2338388 |
| Cluster 34# 24 of 26 | 279 | 0.46 | -0.99 | 13 | 183.21 | 69.48 | 2 | 5 | 4 | 0 | 0.7 | CHEMBL1326975 |
| Cluster 34# 25 of 26 | 280 | 0.43 | -0.29 | 14 | 198.22 | 80.92 | 4 | 4 | 4 | 0 | 0.75 | CHEMBL573104 |
| Cluster 34# 26 of 26 | 281 | 0.4 | -0.17 | 15 | 211.21 | 79.65 | 2 | 5 | 4 | 0 | 0.73 | CHEMBL1437597 |
| Cluster 35# 1 of 13 | 282 | 0.46 | -1.39 | 13 | 184.19 | 86.47 | 3 | 5 | 3 | 0 | 0.76 | CHEMBL229969 |
| Cluster 35# 2 of 13 | 283 | 0.5 | -1.77 | 12 | 170.17 | 86.47 | 3 | 5 | 3 | 0 | 0.74 | CHEMBL229969 |
| Cluster 35# 3 of 13 | 284 | 0.5 | -1.5 | 12 | 168.15 | 82.45 | 3 | 5 | 3 | 0 | 0.7 | CHEMBL2420831 |
| Cluster 35# 4 of 13 | 285 | 0.5 | -1.39 | 12 | 167.17 | 88.24 | 3 | 5 | 3 | 0 | 0.7 | CHEMBL126933 |
| Cluster 35# 5 of 13 | 286 | 0.46 | -1.27 | 13 | 182.18 | 82.45 | 3 | 5 | 3 | 0 | 0.68 | CHEMBL204062 |
| Cluster 35# 6 of 13 | 287 | 0.5 | -0.76 | 12 | 166.18 | 66.24 | 2 | 4 | 2 | 0 | 0.76 | CHEMBL376559 |
| Cluster 35# 7 of 13 | 288 | 0.5 | -1.11 | 12 | 168.22 | 86.78 | 3 | 4 | 3 | 0 | 0.75 | CHEMBL360196 |
| Cluster 35# 8 of 13 | 289 | 0.5 | -1.23 | 12 | 169.2 | 80.99 | 3 | 4 | 3 | 0 | 0.75 | CHEMBL1080923 |
| Cluster 35# 9 of 13 | 290 | 0.5 | -1.77 | 12 | 170.17 | 86.47 | 3 | 5 | 3 | 0 | 0.74 | CHEMBL229969 |
| Cluster 35# 10 of 13 | 291 | 0.5 | -2.06 | 12 | 169.14 | 95.34 | 3 | 6 | 3 | 0 | 0.7 | CHEMBL270708 |
| Cluster 35# 11 of 13 | 292 | 0.5 | -1.95 | 12 | 168.15 | 101.13 | 3 | 6 | 3 | 0 | 0.69 | CHEMBL2131994 |
| Cluster 35# 12 of 13 | 293 | 0.5 | -1.7 | 12 | 170.17 | 86.47 | 3 | 5 | 3 | 0 | 0.73 | CHEMBL229969 |
| Cluster 35# 13 of 13 | 294 | 0.5 | -1.11 | 12 | 168.22 | 86.78 | 3 | 4 | 3 | 0 | 0.74 | CHEMBL360196 |
| Cluster 36# 1 of 22 | 295 | 0.42 | -0.27 | 12 | 189.24 | 71.17 | 2 | 5 | 4 | 0 | 0.76 | CHEMBL1379790 |
| Cluster 36# 2 of 22 | 296 | 0.42 | -0.69 | 12 | 168.19 | 65.98 | 2 | 4 | 4 | 0 | 0.78 | CHEMBL2134206 |
| Cluster 36# 3 of 22 | 297 | 0.42 | -0.65 | 12 | 172.18 | 78.52 | 3 | 5 | 5 | 0 | 0.74 | CHEMBL1507842 |
| Cluster 36# 4 of 22 | 298 | 0.42 | -0.69 | 12 | 168.19 | 65.98 | 2 | 4 | 4 | 0 | 0.76 | CHEMBL2134206 |
| Cluster 36# 5 of 22 | 299 | 0.42 | -0.45 | 12 | 186.23 | 65.98 | 2 | 4 | 4 | 0 | 0.74 | CHEMBL1888272 |
| Cluster 36# 6 of 22 | 300 | 0.36 | -0.8 | 14 | 217.25 | 88.24 | 2 | 6 | 5 | 0 | 0.75 | CHEMBL1499844 |
| Cluster 36# 7 of 22 | 301 | 0.42 | -0.85 | 12 | 188.21 | 101.13 | 3 | 6 | 4 | 0 | 0.79 | CHEMBL2204874 |
| Cluster 36# 8 of 22 | 302 | 0.42 | 0.33 | 12 | 188.25 | 84.06 | 3 | 5 | 4 | 0 | 0.8 | CHEMBL1544140 |
| Cluster 36# 9 of 22 | 303 | 0.42 | -0.15 | 12 | 186.23 | 87.82 | 3 | 5 | 4 | 0 | 0.78 | CHEMBL1307603 |
| Cluster 36# 10 of 22 | 304 | 0.42 | -0.34 | 12 | 188.27 | 75.75 | 3 | 4 | 4 | 0 | 0.69 | CHEMBL238557 |
| Cluster 36# 11 of 22 | 305 | 0.42 | -1.18 | 12 | 172.14 | 98.58 | 2 | 6 | 4 | 0 | 0.75 | CHEMBL1970770 |
| Cluster 36# 12 of 22 | 306 | 0.42 | -0.69 | 12 | 188.2 | 85.44 | 2 | 5 | 4 | 0 | 0.62 | CHEMBL1721136 |
| Cluster 36# 13 of 22 | 307 | 0.42 | -0.64 | 12 | 188.27 | 75.75 | 3 | 4 | 4 | 0 | 0.65 | CHEMBL128763 |
| Cluster 36# 14 of 22 | 308 | 0.42 | -0.68 | 12 | 168.15 | 89.35 | 2 | 5 | 3 | 0 | 0.68 | CHEMBL1982842 |
| Cluster 36# 15 of 22 | 309 | 0.42 | -0.57 | 12 | 173.17 | 91.41 | 3 | 6 | 5 | 0 | 0.71 | CHEMBL512738 |
| Cluster 36# 16 of 22 | 310 | 0.38 | 0.27 | 13 | 199.23 | 89.17 | 3 | 5 | 4 | 0 | 0.74 | CHEMBL377547 |
| Cluster 36# 17 of 22 | 311 | 0.42 | -1.24 | 12 | 188.21 | 104.89 | 3 | 6 | 4 | 0 | 0.7 | CHEMBL1556467 |
| Cluster 36# 18 of 22 | 312 | 0.36 | -0.74 | 14 | 196.16 | 109.48 | 2 | 6 | 4 | 0 | 0.7 | CHEMBL1584205 |
| Cluster 36# 19 of 22 | 313 | 0.42 | -0.49 | 12 | 188.27 | 63.58 | 3 | 4 | 4 | 0 | 0.67 | CHEMBL360471 |
| Cluster 36# 20 of 22 | 314 | 0.42 | -0.12 | 12 | 166.18 | 65.98 | 2 | 4 | 3 | 0 | 0.72 | CHEMBL1727476 |
| Cluster 36# 21 of 22 | 315 | 0.42 | -0.12 | 12 | 166.18 | 65.98 | 2 | 4 | 3 | 0 | 0.69 | CHEMBL1579060 |
| Cluster 36# 22 of 22 | 316 | 0.42 | 0.63 | 12 | 186.6 | 65.98 | 2 | 4 | 3 | 0 | 0.71 | CHEMBL1330256 |
| Cluster 37# 1 of 3 | 317 | 0.42 | -1.51 | 12 | 171.2 | 84.3 | 3 | 5 | 3 | 0 | 0.81 | CHEMBL2326725 |
| Cluster 37# 2 of 3 | 318 | 0.42 | -0.76 | 12 | 191.62 | 84.3 | 3 | 5 | 3 | 0 | 0.8 | CHEMBL2326726 |
| Cluster 37# 3 of 3 | 319 | 0.38 | -1.13 | 13 | 185.22 | 84.3 | 3 | 5 | 3 | 0 | 0.79 | CHEMBL2326725 |
| Cluster 38# 1 of 7 | 320 | 0.46 | -0.83 | 13 | 190.24 | 69.92 | 3 | 4 | 4 | 0 | 0.8 | CHEMBL93061 |
| Cluster 38# 2 of 7 | 321 | 0.46 | -1.15 | 13 | 190.26 | 65.13 | 4 | 4 | 4 | 0 | 0.71 | CHEMBL152232 |
| Cluster 38# 3 of 7 | 322 | 0.46 | -0.74 | 13 | 208.28 | 69.92 | 3 | 4 | 4 | 0 | 0.77 | CHEMBL2009042 |
| Cluster 38# 4 of 7 | 323 | 0.46 | -0.74 | 13 | 208.28 | 69.92 | 3 | 4 | 4 | 0 | 0.69 | CHEMBL2009042 |
| Cluster 38# 5 of 7 | 324 | 0.46 | -0.9 | 13 | 190.26 | 77.3 | 4 | 4 | 4 | 0 | 0.76 | CHEMBL319008 |
| Cluster 38# 6 of 7 | 325 | 0.46 | -1.03 | 13 | 190.26 | 54.13 | 3 | 4 | 5 | 0 | 0.67 | CHEMBL333699 |
| Cluster 38# 7 of 7 | 326 | 0.46 | -0.9 | 13 | 190.26 | 77.3 | 4 | 4 | 4 | 0 | 0.74 | CHEMBL207419 |
| Cluster 39# 1 of 5 | 327 | 0.43 | -0.78 | 14 | 195.22 | 79.45 | 2 | 5 | 4 | 0 | 0.73 | CHEMBL1091253 |
| Cluster 39# 2 of 5 | 328 | 0.43 | -1.27 | 14 | 196.21 | 92.34 | 2 | 6 | 4 | 0 | 0.75 | CHEMBL1089655 |
| Cluster 39# 3 of 5 | 329 | 0.46 | -1.29 | 13 | 182.18 | 73.66 | 2 | 5 | 3 | 0 | 0.67 | CHEMBL204062 |
| Cluster 39# 4 of 5 | 330 | 0.46 | -1.18 | 13 | 181.19 | 79.45 | 2 | 5 | 3 | 0 | 0.67 | CHEMBL1092963 |
| Cluster 39# 5 of 5 | 331 | 0.46 | -1.74 | 13 | 182.18 | 92.34 | 2 | 6 | 3 | 0 | 0.73 | CHEMBL33851 |
| Cluster 40# 1 of 16 | 332 | 0.4 | -0.21 | 15 | 211.21 | 78.79 | 3 | 5 | 5 | 0 | 0.77 | CHEMBL1544325 |
| Cluster 40# 2 of 16 | 333 | 0.4 | 0.8 | 15 | 212.24 | 58.92 | 2 | 4 | 5 | 0 | 0.81 | CHEMBL256637 |
| Cluster 40# 3 of 16 | 334 | 0.43 | -0.75 | 14 | 216.28 | 88.33 | 4 | 4 | 4 | 0 | 0.77 | CHEMBL161571 |
| Cluster 40# 4 of 16 | 335 | 0.38 | -0.64 | 16 | 225.24 | 89.79 | 4 | 5 | 5 | 0 | 0.72 | CHEMBL190452 |
| Cluster 40# 5 of 16 | 336 | 0.4 | 1.39 | 15 | 226.29 | 49.69 | 2 | 3 | 5 | 0 | 0.76 | CHEMBL512867 |
| Cluster 40# 6 of 16 | 337 | 0.4 | 0.06 | 15 | 210.23 | 66.76 | 2 | 4 | 5 | 0 | 0.74 | CHEMBL226919 |
| Cluster 40# 7 of 16 | 338 | 0.4 | -2.07 | 15 | 212.2 | 115.81 | 5 | 6 | 4 | 0 | 0.79 | CHEMBL189913 |
| Cluster 40# 8 of 16 | 339 | 0.4 | -1.68 | 15 | 211.21 | 103.78 | 4 | 5 | 4 | 0 | 0.81 | CHEMBL3298397 |
| Cluster 40# 9 of 16 | 340 | 0.43 | 0.14 | 14 | 192.19 | 92 | 2 | 5 | 4 | 0 | 0.75 | CHEMBL524839 |
| Cluster 40# 10 of 16 | 341 | 0.4 | -1.41 | 15 | 215.21 | 110.72 | 3 | 7 | 5 | 0 | 0.82 | CHEMBL3233521 |
| Cluster 40# 11 of 16 | 342 | 0.43 | -1.84 | 14 | 215.23 | 109.33 | 3 | 6 | 4 | 0 | 0.77 | CHEMBL1880741 |
| Cluster 40# 12 of 16 | 343 | 0.43 | -1.73 | 14 | 216.21 | 103.54 | 3 | 6 | 4 | 0 | 0.77 | CHEMBL1313775 |
| Cluster 40# 13 of 16 | 344 | 0.43 | -1.73 | 14 | 214.24 | 115.12 | 3 | 6 | 4 | 0 | 0.77 | CHEMBL1870792 |
| Cluster 40# 14 of 16 | 345 | 0.4 | -0.44 | 15 | 208.21 | 93.41 | 4 | 5 | 5 | 0 | 0.76 | CHEMBL230031 |
| Cluster 40# 15 of 16 | 346 | 0.43 | -1.16 | 14 | 196.2 | 98.57 | 3 | 5 | 4 | 0 | 0.71 | CHEMBL422725 |
| Cluster 40# 16 of 16 | 347 | 0.4 | -1.56 | 15 | 210.23 | 109.57 | 4 | 5 | 4 | 0 | 0.77 | CHEMBL1889942 |
| Cluster 41# 1 of 6 | 348 | 0.38 | -0.26 | 13 | 200.26 | 76.21 | 2 | 4 | 5 | 0 | 0.61 | CHEMBL1425549 |
| Cluster 41# 2 of 6 | 349 | 0.38 | -0.94 | 13 | 201.25 | 88.24 | 3 | 5 | 5 | 0 | 0.62 | CHEMBL295515 |
| Cluster 41# 3 of 6 | 350 | 0.42 | -0.49 | 12 | 168.19 | 65.98 | 2 | 4 | 5 | 0 | 0.77 | CHEMBL1933838 |
| Cluster 41# 4 of 6 | 351 | 0.42 | -1.69 | 12 | 172.2 | 85.75 | 4 | 5 | 5 | 0 | 0.75 | CHEMBL1367784 |
| Cluster 41# 5 of 6 | 352 | 0.38 | -1.03 | 13 | 181.19 | 78.34 | 2 | 5 | 4 | 0 | 0.78 | CHEMBL3191578 |
| Cluster 41# 6 of 6 | 353 | 0.38 | -1.54 | 13 | 185.16 | 85.33 | 1 | 6 | 4 | 0 | 0.8 | CHEMBL267005 |
| Cluster 42# 1 of 2 | 354 | 0.5 | -1.64 | 12 | 176.23 | 65.13 | 4 | 4 | 3 | 0 | 0.72 | CHEMBL270037 |
| Cluster 42# 2 of 2 | 355 | 0.5 | -1.04 | 12 | 176.21 | 69.92 | 3 | 4 | 3 | 0 | 0.67 | CHEMBL1160001 |
| Cluster 43# 1 of 26 | 356 | 0.46 | 0.35 | 13 | 181.19 | 73.54 | 3 | 4 | 4 | 0 | 0.76 | CHEMBL234818 |
| Cluster 43# 2 of 26 | 357 | 0.46 | -1.44 | 13 | 182.2 | 104.1 | 5 | 5 | 3 | 0 | 0.78 | CHEMBL3092337 |
| Cluster 43# 3 of 26 | 358 | 0.46 | -1.19 | 13 | 183.23 | 94.12 | 4 | 4 | 3 | 0 | 0.78 | CHEMBL132012 |
| Cluster 43# 4 of 26 | 359 | 0.46 | -1.33 | 13 | 183.18 | 98.31 | 5 | 5 | 3 | 0 | 0.78 | CHEMBL42751 |
| Cluster 43# 5 of 26 | 360 | 0.46 | -1.08 | 13 | 184.21 | 88.33 | 4 | 4 | 3 | 0 | 0.78 | CHEMBL3250637 |
| Cluster 43# 6 of 26 | 361 | 0.4 | 0.4 | 15 | 210.23 | 69.92 | 3 | 4 | 4 | 0 | 0.76 | CHEMBL598458 |
| Cluster 43# 7 of 26 | 362 | 0.46 | -1.44 | 13 | 182.2 | 104.1 | 5 | 5 | 3 | 0 | 0.78 | CHEMBL2041423 |
| Cluster 43# 8 of 26 | 363 | 0.46 | -2.27 | 13 | 183.16 | 108.47 | 4 | 6 | 3 | 0 | 0.74 | CHEMBL1824882 |
| Cluster 43# 9 of 26 | 364 | 0.46 | -1.19 | 13 | 183.23 | 94.12 | 4 | 4 | 3 | 0 | 0.77 | CHEMBL131383 |
| Cluster 43# 10 of 26 | 365 | 0.46 | -1.5 | 13 | 183.19 | 115.51 | 5 | 6 | 3 | 0 | 0.73 | CHEMBL122954 |
| Cluster 43# 11 of 26 | 366 | 0.5 | -1.03 | 12 | 169.18 | 62.58 | 2 | 4 | 4 | 0 | 0.73 | CHEMBL134733 |
| Cluster 43# 12 of 26 | 367 | 0.46 | -0.94 | 13 | 185.18 | 95.94 | 4 | 5 | 3 | 0 | 0.75 | CHEMBL164378 |
| Cluster 43# 13 of 26 | 368 | 0.46 | -0.58 | 13 | 182.22 | 69.72 | 3 | 4 | 3 | 0 | 0.74 | CHEMBL1774331 |
| Cluster 43# 14 of 26 | 369 | 0.43 | -1.13 | 14 | 200.19 | 95.32 | 3 | 6 | 4 | 0 | 0.79 | CHEMBL1950821 |
| Cluster 43# 15 of 26 | 370 | 0.46 | -0.48 | 13 | 183.2 | 63.93 | 3 | 4 | 3 | 0 | 0.75 | CHEMBL161159 |
| Cluster 43# 16 of 26 | 371 | 0.5 | -0.86 | 12 | 167.21 | 84.3 | 4 | 4 | 3 | 0 | 0.7 | CHEMBL41355 |
| Cluster 43# 17 of 26 | 372 | 0.46 | 0.5 | 13 | 180.2 | 60.69 | 3 | 3 | 3 | 0 | 0.68 | CHEMBL1460667 |
| Cluster 43# 18 of 26 | 373 | 0.46 | -0.33 | 13 | 184.19 | 69.92 | 3 | 4 | 4 | 0 | 0.71 | CHEMBL2024611 |
| Cluster 43# 19 of 26 | 374 | 0.43 | -1.15 | 14 | 197.21 | 98.31 | 5 | 5 | 4 | 0 | 0.76 | CHEMBL3287832 |
| Cluster 43# 20 of 26 | 375 | 0.46 | -0.33 | 13 | 184.19 | 69.92 | 3 | 4 | 4 | 0 | 0.72 | CHEMBL256964 |
| Cluster 43# 21 of 26 | 376 | 0.46 | 0.38 | 13 | 180.2 | 60.69 | 3 | 3 | 2 | 0 | 0.75 | CHEMBL167054 |
| Cluster 43# 22 of 26 | 377 | 0.43 | 0.76 | 14 | 196.24 | 60.69 | 3 | 3 | 4 | 0 | 0.73 | CHEMBL3275076 |
| Cluster 43# 23 of 26 | 378 | 0.46 | -1.27 | 13 | 183.21 | 107.52 | 4 | 5 | 3 | 0 | 0.86 | CHEMBL493351 |
| Cluster 43# 24 of 26 | 379 | 0.43 | 0.95 | 14 | 200.21 | 49.69 | 2 | 3 | 4 | 0 | 0.79 | CHEMBL35729 |
| Cluster 43# 25 of 26 | 380 | 0.43 | 0.2 | 14 | 198.22 | 69.92 | 3 | 4 | 4 | 0 | 0.68 | CHEMBL8121 |
| Cluster 43# 26 of 26 | 381 | 0.43 | 0.9 | 14 | 194.23 | 49.69 | 2 | 3 | 4 | 0 | 0.67 | CHEMBL1460667 |
| Cluster 44# 1 of 6 | 382 | 0.46 | 0.15 | 13 | 180.22 | 68.1 | 3 | 3 | 3 | 0 | 0.84 | CHEMBL85122 |
| Cluster 44# 2 of 6 | 383 | 0.5 | -0.46 | 12 | 166.18 | 76.21 | 2 | 4 | 3 | 0 | 0.77 | CHEMBL1922744 |
| Cluster 44# 3 of 6 | 384 | 0.5 | -0.57 | 12 | 167.16 | 70.42 | 2 | 4 | 3 | 0 | 0.77 | CHEMBL81798 |
| Cluster 44# 4 of 6 | 385 | 0.46 | -0.15 | 13 | 181.19 | 70.42 | 2 | 4 | 3 | 0 | 0.79 | CHEMBL81798 |
| Cluster 44# 5 of 6 | 386 | 0.43 | -1.39 | 14 | 195.18 | 98.21 | 2 | 6 | 3 | 0 | 0.76 | CHEMBL1983139 |
| Cluster 44# 6 of 6 | 387 | 0.46 | -1.09 | 13 | 181.19 | 89.1 | 2 | 5 | 3 | 0 | 0.76 | CHEMBL314158 |
| Cluster 45# 1 of 1 | 388 | 0.4 | -0.68 | 15 | 211.21 | 92.78 | 3 | 5 | 4 | 0 | 0.77 | CHEMBL90460 |
| Cluster 46# 1 of 2 | 389 | 0.46 | -2.46 | 13 | 190.22 | 71.2 | 3 | 5 | 4 | 0 | 0.7 | CHEMBL1822736 |
| Cluster 46# 2 of 2 | 390 | 0.46 | -2.46 | 13 | 190.22 | 71.2 | 3 | 5 | 4 | 0 | 0.7 | CHEMBL1822736 |
| Cluster 47# 1 of 4 | 391 | 0.4 | -0.85 | 15 | 208.21 | 92.42 | 3 | 5 | 5 | 0 | 0.81 | CHEMBL1761620 |
| Cluster 47# 2 of 4 | 392 | 0.4 | -0.78 | 15 | 211.21 | 78.79 | 3 | 5 | 5 | 0 | 0.71 | CHEMBL386111 |
| Cluster 47# 3 of 4 | 393 | 0.43 | -0.72 | 14 | 196.2 | 84.58 | 3 | 5 | 4 | 0 | 0.69 | CHEMBL3184844 |
| Cluster 47# 4 of 4 | 394 | 0.4 | -0.89 | 15 | 209.22 | 70.28 | 2 | 5 | 5 | 0 | 0.8 | CHEMBL1319273 |
| Cluster 48# 1 of 3 | 395 | 0.43 | -1.69 | 14 | 200.19 | 95.7 | 3 | 6 | 4 | 0 | 0.84 | CHEMBL3288113 |
| Cluster 48# 2 of 3 | 396 | 0.43 | -0.13 | 14 | 195.22 | 72.55 | 2 | 4 | 4 | 0 | 0.81 | CHEMBL209769 |
| Cluster 48# 3 of 3 | 397 | 0.43 | -1.69 | 14 | 200.19 | 95.7 | 3 | 6 | 4 | 0 | 0.78 | CHEMBL3288113 |
| Cluster 49# 1 of 3 | 398 | 0.42 | -1.64 | 12 | 174.19 | 77.76 | 3 | 4 | 3 | 0 | 0.75 | CHEMBL1760122 |
| Cluster 49# 2 of 3 | 399 | 0.38 | -0.73 | 13 | 186.21 | 66.76 | 2 | 4 | 4 | 0 | 0.77 | CHEMBL470443 |
| Cluster 49# 3 of 3 | 400 | 0.42 | -2.68 | 12 | 173.19 | 85.82 | 4 | 5 | 3 | 0 | 0.75 | CHEMBL1822655 |
| Cluster 50# 1 of 4 | 401 | 0.36 | -0.85 | 14 | 197.23 | 81.14 | 2 | 5 | 4 | 0 | 0.71 | CHEMBL1770760 |
| Cluster 50# 2 of 4 | 402 | 0.38 | -0.09 | 13 | 200.26 | 76.21 | 2 | 4 | 4 | 0 | 0.79 | CHEMBL2094720 |
| Cluster 50# 3 of 4 | 403 | 0.38 | -0.09 | 13 | 200.26 | 76.21 | 2 | 4 | 4 | 0 | 0.76 | CHEMBL2094720 |
| Cluster 50# 4 of 4 | 404 | 0.38 | 0.2 | 13 | 202.3 | 75.75 | 3 | 4 | 4 | 0 | 0.7 | CHEMBL238558 |
| Cluster 51# 1 of 9 | 405 | 0.36 | -0.92 | 14 | 215.27 | 79.45 | 2 | 5 | 4 | 0 | 0.77 | CHEMBL2417597 |
| Cluster 51# 2 of 9 | 406 | 0.38 | -0.79 | 13 | 203.22 | 98.21 | 2 | 6 | 4 | 0 | 0.77 | CHEMBL1307689 |
| Cluster 51# 3 of 9 | 407 | 0.38 | -0.1 | 13 | 202.3 | 75.75 | 3 | 4 | 4 | 0 | 0.69 | CHEMBL241747 |
| Cluster 51# 4 of 9 | 408 | 0.36 | -1.04 | 14 | 214.24 | 93.28 | 2 | 5 | 4 | 0 | 0.7 | CHEMBL2000751 |
| Cluster 51# 5 of 9 | 409 | 0.38 | -0.45 | 13 | 202.23 | 85.44 | 2 | 5 | 4 | 0 | 0.62 | CHEMBL1721136 |
| Cluster 51# 6 of 9 | 410 | 0.36 | -0.06 | 14 | 214.24 | 71.78 | 1 | 5 | 5 | 0 | 0.6 | CHEMBL3210709 |
| Cluster 51# 7 of 9 | 411 | 0.38 | -0.27 | 13 | 185.18 | 85.69 | 2 | 5 | 4 | 0 | 0.61 | CHEMBL1521511 |
| Cluster 51# 8 of 9 | 412 | 0.36 | 0.15 | 14 | 216.32 | 63.58 | 3 | 4 | 5 | 0 | 0.65 | CHEMBL128763 |
| Cluster 51# 9 of 9 | 413 | 0.36 | 0.15 | 14 | 216.32 | 63.58 | 3 | 4 | 5 | 0 | 0.67 | CHEMBL128763 |
| Cluster 52# 1 of 2 | 414 | 0.38 | -1.14 | 13 | 188.22 | 77.76 | 3 | 4 | 4 | 0 | 0.71 | CHEMBL1760121 |
| Cluster 52# 2 of 2 | 415 | 0.42 | -1.09 | 12 | 172.18 | 66.76 | 2 | 4 | 3 | 0 | 0.67 | CHEMBL3184614 |
| Cluster 53# 1 of 3 | 416 | 0.38 | 0.6 | 16 | 227.26 | 73.94 | 2 | 5 | 6 | 0 | 0.78 | CHEMBL3247078 |
| Cluster 53# 2 of 3 | 417 | 0.38 | 0.6 | 16 | 227.26 | 73.94 | 2 | 5 | 6 | 0 | 0.78 | CHEMBL3247078 |
| Cluster 53# 3 of 3 | 418 | 0.4 | -0.14 | 15 | 213.23 | 84.94 | 3 | 5 | 5 | 0 | 0.82 | CHEMBL3247078 |
| Cluster 54# 1 of 1 | 419 | 0.33 | -0.71 | 15 | 212.2 | 92.28 | 2 | 6 | 5 | 0 | 0.87 | CHEMBL1501027 |
| Cluster 55# 1 of 1 | 420 | 0.43 | -0.22 | 14 | 197.23 | 69.48 | 2 | 5 | 5 | 0 | 0.73 | CHEMBL1416377 |
| Cluster 56# 1 of 5 | 421 | 0.36 | -0.1 | 14 | 196.25 | 65.98 | 2 | 4 | 5 | 0 | 0.79 | CHEMBL1210369 |
| Cluster 56# 2 of 5 | 422 | 0.36 | -0.1 | 14 | 196.25 | 65.98 | 2 | 4 | 5 | 0 | 0.78 | CHEMBL1210369 |
| Cluster 56# 3 of 5 | 423 | 0.38 | -0.1 | 13 | 180.2 | 65.98 | 2 | 4 | 5 | 0 | 0.75 | CHEMBL517376 |
| Cluster 56# 4 of 5 | 424 | 0.38 | -0.33 | 13 | 182.22 | 65.98 | 2 | 4 | 5 | 0 | 0.75 | CHEMBL1210369 |
| Cluster 56# 5 of 5 | 425 | 0.38 | -0.1 | 13 | 180.2 | 65.98 | 2 | 4 | 5 | 0 | 0.76 | CHEMBL517376 |
| Cluster 57# 1 of 10 | 426 | 0.42 | -0.68 | 12 | 172.22 | 49.69 | 2 | 3 | 4 | 0 | 0.69 | CHEMBL194257 |
| Cluster 57# 2 of 10 | 427 | 0.42 | 0.19 | 12 | 174.24 | 60.69 | 3 | 3 | 4 | 0 | 0.66 | CHEMBL1760094 |
| Cluster 57# 3 of 10 | 428 | 0.42 | -1.25 | 12 | 174.19 | 66.76 | 2 | 4 | 4 | 0 | 0.66 | CHEMBL1902338 |
| Cluster 57# 4 of 10 | 429 | 0.42 | -2.27 | 12 | 175.18 | 78.79 | 3 | 5 | 4 | 0 | 0.71 | CHEMBL424399 |
| Cluster 57# 5 of 10 | 430 | 0.42 | -2.27 | 12 | 175.18 | 78.79 | 3 | 5 | 4 | 0 | 0.7 | CHEMBL2135122 |
| Cluster 57# 6 of 10 | 431 | 0.42 | -0.68 | 12 | 172.22 | 49.69 | 2 | 3 | 4 | 0 | 0.74 | CHEMBL194257 |
| Cluster 57# 7 of 10 | 432 | 0.42 | -0.76 | 12 | 192.28 | 60.69 | 3 | 3 | 4 | 0 | 0.63 | CHEMBL2218923 |
| Cluster 57# 8 of 10 | 433 | 0.42 | 0.21 | 12 | 171.24 | 52.82 | 2 | 3 | 4 | 0 | 0.76 | CHEMBL327133 |
| Cluster 57# 9 of 10 | 434 | 0.38 | -0.29 | 13 | 186.25 | 49.69 | 2 | 3 | 4 | 0 | 0.72 | CHEMBL194257 |
| Cluster 57# 10 of 10 | 435 | 0.42 | -0.68 | 12 | 172.22 | 60.69 | 3 | 3 | 3 | 0 | 0.64 | CHEMBL2138280 |
| Cluster 58# 1 of 1 | 436 | 0.75 | 0.68 | 16 | 216.24 | 65.98 | 2 | 4 | 3 | 0 | 0.79 | CHEMBL3091516 |
| Cluster 59# 1 of 1 | 437 | 0.79 | 0.11 | 14 | 208.24 | 84.92 | 2 | 5 | 2 | 0 | 0.64 | CHEMBL2164857 |
| Cluster 60# 1 of 1 | 438 | 0.71 | 0.42 | 14 | 192.23 | 57.07 | 3 | 3 | 2 | 0 | 0.78 | CHEMBL491881 |
| Cluster 61# 1 of 1 | 439 | 0.62 | -0.1 | 16 | 218.21 | 83.31 | 2 | 5 | 2 | 0 | 0.68 | CHEMBL1306701 |
| Cluster 62# 1 of 1 | 440 | 0.56 | -1.92 | 16 | 222.2 | 100.87 | 2 | 7 | 4 | 0 | 0.77 | CHEMBL2093424 |
| Cluster 63# 1 of 2 | 441 | 0.56 | -1.51 | 16 | 221.21 | 87.98 | 2 | 6 | 3 | 0 | 0.81 | CHEMBL1437558 |
| Cluster 63# 2 of 2 | 442 | 0.6 | -1.72 | 15 | 210.19 | 122.97 | 3 | 8 | 3 | 0 | 0.78 | CHEMBL130251 |
| Cluster 64# 1 of 1 | 443 | 0.69 | 0.32 | 13 | 179.22 | 53.35 | 2 | 3 | 3 | 0 | 0.75 | CHEMBL226002 |
| Cluster 65# 1 of 1 | 444 | 0.56 | -0.87 | 16 | 223.23 | 98.33 | 2 | 6 | 5 | 0 | 0.76 | CHEMBL87269 |
| Cluster 66# 1 of 2 | 445 | 0.42 | -0.79 | 12 | 171.19 | 66.49 | 2 | 4 | 5 | 0 | 0.7 | CHEMBL398746 |
| Cluster 66# 2 of 2 | 446 | 0.42 | -0.37 | 12 | 189.24 | 71.17 | 2 | 5 | 4 | 0 | 0.79 | CHEMBL1379790 |
| Cluster 67# 1 of 1 | 447 | 0.43 | 0.44 | 14 | 193.24 | 53.35 | 2 | 3 | 5 | 0 | 0.66 | CHEMBL1206076 |
| Cluster 68# 1 of 4 | 448 | 0.42 | -1.09 | 12 | 171.18 | 82.75 | 2 | 6 | 4 | 0 | 0.75 | CHEMBL1721717 |
| Cluster 68# 2 of 4 | 449 | 0.36 | -1.24 | 14 | 198.16 | 107.28 | 2 | 7 | 4 | 0 | 0.61 | CHEMBL2268643 |
| Cluster 68# 3 of 4 | 450 | 0.42 | 0.12 | 12 | 188.25 | 75.27 | 2 | 5 | 4 | 0 | 0.68 | CHEMBL1544140 |
| Cluster 68# 4 of 4 | 451 | 0.38 | -0.81 | 13 | 202.23 | 92.34 | 2 | 6 | 4 | 0 | 0.67 | CHEMBL2204874 |
| Cluster 69# 1 of 1 | 452 | 0.42 | -0.42 | 12 | 192.28 | 49.69 | 2 | 3 | 5 | 0 | 0.72 | CHEMBL1967343 |
| Cluster 70# 1 of 2 | 453 | 0.38 | -0.68 | 16 | 225.24 | 98.33 | 2 | 6 | 5 | 0 | 0.84 | CHEMBL1393877 |
| Cluster 70# 2 of 2 | 454 | 0.4 | -0.92 | 15 | 211.22 | 98.33 | 2 | 6 | 5 | 0 | 0.82 | CHEMBL1834387 |
| Cluster 71# 1 of 1 | 455 | 0.36 | -1.63 | 14 | 199.21 | 89.45 | 3 | 6 | 5 | 0 | 0.77 | CHEMBL2130650 |
| Cluster 72# 1 of 1 | 456 | 0.36 | -1.98 | 14 | 201.18 | 121.46 | 4 | 7 | 5 | 0 | 0.74 | CHEMBL2171392 |
| Cluster 73# 1 of 1 | 457 | 0.36 | -1.41 | 14 | 200.22 | 99.49 | 5 | 6 | 5 | 0 | 0.75 | CHEMBL484480 |
| Cluster 74# 1 of 1 | 458 | 0.33 | 0.78 | 15 | 229.3 | 71.17 | 2 | 5 | 6 | 0 | 0.71 | CHEMBL3208754 |
| Cluster 75# 1 of 1 | 459 | 0.33 | -0.5 | 15 | 210.19 | 109.48 | 2 | 6 | 4 | 0 | 0.69 | CHEMBL1584205 |
| Cluster 76# 1 of 1 | 460 | 0.33 | -1.75 | 12 | 171.15 | 86.63 | 3 | 5 | 3 | 0 | 0.74 | CHEMBL381268 |

**Table S3**. Compounds yielded by the ROCS^#^ search.

| **SMILES** | **ROCS  Tani Combo** | **ROCS  Shape Tani** | **ROCS Color Tani** | **ROCS Ref T^#^** | **ROCS Ref Color T^#^** | **ROCS Ref T^#^ Combo** | **ROCS Fit T^#^** | **ROCS Fit Color  T^#^** | **ROCS Fit T^#^ Combo** | **ROCS Color Score** | **ROCS Overlap** | **ROCS Rank** |
| --- | --- | --- | --- | --- | --- | --- | --- | --- | --- | --- | --- | --- |
| c1nc(c(n1[C@H]2[C@@H]([C@@H]([C@H](O2)CO)O)O)Cl)C(=O)N | 1.047 | 0.92 | 0.127 | 0.924 | 0.128 | 1.051 | 1 | 0.93 | 1.93 | -18.568 | 805.268 | 485 |
| c1ccc(cc1)Cn2ccc3c2ccnc3O | 1.033 | 0.862 | 0.171 | 0.885 | 0.16 | 1.045 | 0.97 | 1.735 | 2.706 | -23.152 | 770.98 | 595 |
| c1nc(c(c(n1)NC[C@@H]2CCCS2(=O)=O)Cl)N | 1.027 | 0.858 | 0.169 | 0.851 | 0.161 | 1.012 | 1.01 | 1.443 | 2.453 | -23.349 | 738.256 | 640 |
| c1cc(c2c(c1N)non2)N3CCCCCC3 | 1.021 | 0.87 | 0.15 | 0.879 | 0.144 | 1.023 | 0.989 | 1.458 | 2.446 | -20.839 | 764.838 | 707 |
| c1nc(c(c(n1)NC[C@@H]2CCCO2)[N+](=O)[O-])N | 1.012 | 0.842 | 0.17 | 0.863 | 0.163 | 1.026 | 0.971 | 1.377 | 2.348 | -23.611 | 751.133 | 803 |
| C[C@@H](CNc1c(c(ns1)N)C#N)CO | 1.011 | 0.803 | 0.207 | 0.752 | 0.191 | 0.943 | 1.093 | 1.713 | 2.806 | -27.702 | 647.052 | 819 |
| c1c(nc(s1)[C@H]2[C@@H]([C@@H]([C@H](O2)CO)O)O)C(=O)N | 1.01 | 0.88 | 0.13 | 0.871 | 0.13 | 1.001 | 1.012 | 1 | 2.012 | -18.869 | 756.913 | 828 |
| CC1(CN(CCS1)C(=O)Cc2ccnc(c2Br)O)C | 1.009 | 0.841 | 0.169 | 0.88 | 0.16 | 1.039 | 0.949 | 1.52 | 2.47 | -23.143 | 767.227 | 838 |
| COC(=O)C1CCN(CC1)c2cc(ncn2)N | 1.009 | 0.838 | 0.171 | 0.857 | 0.16 | 1.017 | 0.974 | 1.63 | 2.604 | -23.271 | 744.927 | 847 |
| CC(=O)N1CCN(C[C@@H](C1)O)c2ccnc(n2)N | 1.008 | 0.792 | 0.216 | 0.851 | 0.197 | 1.048 | 0.92 | 1.769 | 2.688 | -28.628 | 741.753 | 860 |
| C[C@H]1C[C@@H](CCO1)CNc2c(c(ncn2)N)Cl | 1.007 | 0.838 | 0.17 | 0.846 | 0.16 | 1.007 | 0.988 | 1.527 | 2.515 | -23.249 | 735.179 | 865 |

^#^ - ROCS is a shape-based superposition method, more at: https://docs.eyesopen.com/applications/rocs/rocs/rocs.html

T^#^ = **Tversky**

Tani = **Tanimoto**

- TanimotoCombo - the sum of Shape Tanimoto and Color Tanimoto
- RefTverskyCombo - the sum of Ref Shape Tversky and Ref Color Tversky
- FitTverskyCombo - the sum of Fit Shape Tversky and Fit Color Tversky

ROCS manual and explanation at https://docs.eyesopen.com/applications/rocs/rocs/rocs_report_file.html

**Table S4**. Comparison of the OEDOCKING Hybrid Chemgauss4 score (blue shading) with the CNN Affinity score calculated by Gnina (green shading). Both re-scoring approaches, of the best 98 compounds, identified using the OpenEye software, revealed convergent results.

| **SMILES** | **TITLE** | **HYBRID  Chemgauss4 score** | **Pose  Id.** | **Dock  Type** | **Ligand id.** | **Minimized Affinity** | **CNN Score** | **CNN Affinity** |
| --- | --- | --- | --- | --- | --- | --- | --- | --- |
| Cc1nc(c2n1c(ncc2)N)C[C@@H](CO)O | Query Analogs# 5 of 18_2 | -9.92231 | 1 | Hybrid | lig_000 | -7.76384687 | 0.958764732 | 4.85350132 |
| CCc1nc(c2n1c(ncc2)N)CCO | Cluster 6# 1 of 8_11 | -9.912901 | 1 | Hybrid | lig_001 | -6.94783306 | 0.972479165 | 4.99387503 |
| Cc1c2c(nc(nn2c(n1)CCCCO)C)O | Cluster 20# 1 of 1_23 | -9.511113 | 1 | Hybrid | lig_002 | -7.53380203 | 0.753668129 | 4.67874622 |
| c1c2c(ncnc2n(n1)CCCCO)N | Cluster 12# 2 of 14_41 | -9.495018 | 1 | Hybrid | lig_003 | -6.89941597 | 0.942028582 | 4.62793779 |
| C[C@@H](CCO)Cn1c2c(cn1)c(ncn2)N | Cluster 12# 14 of 14_27 | -9.409123 | 1 | Hybrid | lig_004 | -7.198843 | 0.915446162 | 4.69576406 |
| c1cnc(n2c1c(nc2)C[C@@H](CO)O)N | Query Analogs# 4 of 18_5 | -9.35242 | 1 | Hybrid | lig_005 | -7.32551908 | 0.949485779 | 4.59364557 |
| c1nc2c(ncn2c(n1)N)C[C@@H](CO)O | Query Analogs# 11 of 18_21 | -9.279909 | 1 | Hybrid | lig_006 | -7.33806944 | 0.929020107 | 4.44078112 |
| c1cnc(n2c1c(nc2)CCO)N | Cluster 9# 6 of 17_5 | -9.066534 | 1 | Hybrid | lig_007 | -6.70697927 | 0.923818231 | 4.44708061 |
| C[C@@H](CCn1c2c(cn1)c(ncn2)N)CO | Cluster 8# 16 of 20_27 | -9.002323 | 1 | Hybrid | lig_008 | -7.08196402 | 0.949670732 | 4.75042343 |
| c1c2c(ncn2c(nn1)O)C[C@@H](CO)O | Query Analogs# 13 of 18_20 | -8.964443 | 1 | Hybrid | lig_009 | -7.40213346 | 0.669792712 | 4.11392832 |
| c1cc(c(cc1CC(=O)CO)N)NC=O | Cluster 47# 1 of 4_14 | -8.899752 | 1 | Hybrid | lig_010 | -7.04145765 | 0.834595025 | 4.20553255 |
| c1nc2c(ncn2c(n1)O)C[C@@H](CO)O | Query Analogs# 12 of 18_21 | -8.802429 | 1 | Hybrid | lig_011 | -7.43341255 | 0.684551716 | 4.01575327 |
| c1cnc(c2c1n(nn2)C[C@@H](CO)O)N | Query Analogs# 10 of 18_4 | -8.545895 | 1 | Hybrid | lig_012 | -7.38885403 | 0.956479192 | 4.58192158 |
| C1C[C@@H](Cc2c1nc(nc2O)N)CCO | Cluster 17# 1 of 2_9 | -8.508798 | 1 | Hybrid | lig_013 | -7.67232513 | 0.60695231 | 4.20177174 |
| c1c(c2c([nH]1)c(nc(n2)N)O)CCC(=O)CO | Cluster 12# 13 of 14_113 | -8.29709 | 1 | Hybrid | lig_014 | -7.96436644 | 0.876801074 | 4.83254051 |
| CCn1c2c(c(n1)CCO)ncnc2O | Cluster 6# 5 of 8_7 | -8.10346 | 1 | Hybrid | lig_015 | -6.55951643 | 0.837007284 | 4.42412472 |
| c1c2c(nc(n1)N)CN(C2)C(=O)CO | Cluster 2# 3 of 6_1 | -8.095482 | 1 | Hybrid | lig_016 | -7.28451109 | 0.886925876 | 4.12288857 |
| c1cn(c2c1c(ncn2)N)CC[C@H](C[NH3+])CO | Cluster 8# 20 of 20_145 | -8.028592 | 1 | Hybrid | lig_017 | -7.38116121 | 0.883657634 | 4.70329332 |
| **Cc1c(sc(n1)N)c2cnc(o2)CO ^##^** | **Cluster 5# 4 of 5_6** | **-8.02551** | **1** | **Hybrid** | **lig_018** | **-7.18787909** | **0.954337955** | **4.75065708** |
| c1nc(n(n1)/C=N\CC[C@@H](CO)O)N | Cluster 31# 1 of 1_164 | -7.96422 | 1 | Hybrid | lig_019 | -6.85499811 | 0.864241123 | 4.15122747 |
| c1cn(c2c1c(ncn2)N)CCC(CO)CO | Cluster 8# 3 of 20_179 | -7.964111 | 1 | Hybrid | lig_020 | -7.26538944 | 0.934114933 | 4.65104198 |
| C1C[C@@H](Cc2c1nc(nc2N)N)CCO | Cluster 17# 2 of 2_9 | -7.961363 | 1 | Hybrid | lig_021 | -7.49109411 | 0.856088519 | 4.62164354 |
| c1nc(c2c(n1)n(nn2)CCC(CO)CO)N | Cluster 8# 8 of 20_57 | -7.949142 | 1 | Hybrid | lig_022 | -7.29890823 | 0.944298685 | 4.50110531 |
| COCc1nc(c2n1c(nnc2)O)CCO | Cluster 3# 1 of 1_32 | -7.93968 | 1 | Hybrid | lig_023 | -6.59729385 | 0.758113503 | 4.02696991 |
| c1c2c(cc(nn2)O)sc1C(=O)CO | Cluster 13# 6 of 9_8 | -7.799595 | 1 | Hybrid | lig_024 | -7.23124743 | 0.881399095 | 4.28792858 |
| c1nc(c2c(n1)n(nn2)CCCCO)N | Cluster 12# 1 of 14_25 | -7.660542 | 1 | Hybrid | lig_025 | -6.68876743 | 0.949979305 | 4.48630142 |
| Cc1c2c(nc(n1)N)C[C@@H](C2)C(=C)CO | Cluster 2# 6 of 6_11 | -7.656706 | 1 | Hybrid | lig_026 | -8.22394848 | 0.565658927 | 4.52761698 |
| C1CN(Cc2n1c(nn2)CO)C(=O)CO | Cluster 7# 3 of 4_19 | -7.637568 | 1 | Hybrid | lig_027 | -6.91306639 | 0.771988511 | 3.88071251 |
| CCN(c1ccnc(n1)N)C(=O)CO | Cluster 39# 2 of 5_21 | -7.632321 | 1 | Hybrid | lig_028 | -6.72750664 | 0.968055427 | 4.36565447 |
| CSc1c2c(ncnc2n(n1)CCO)N | Cluster 6# 8 of 8_24 | -7.623367 | 1 | Hybrid | lig_029 | -6.63814974 | 0.96774292 | 4.62768078 |
| Cn1c(cc2c1ncc(c2)N)C(=O)CO | Cluster 13# 7 of 9_7 | -7.607549 | 1 | Hybrid | lig_030 | -7.30996609 | 0.875192583 | 4.34822989 |
| c1nc(c2c(n1)n(cn2)CCC(CO)CO)N | Cluster 8# 2 of 20_72 | -7.603827 | 1 | Hybrid | lig_031 | -7.02450275 | 0.977069616 | 4.67779112 |
| c1nc2c(n1C[C@@H](CO)O)c(=O)[nH]c(n2)N | Cluster 22# 1 of 1_19 | -7.581264 | 1 | Hybrid | lig_032 | -7.24865675 | 0.830854952 | 4.2763257 |
| c1c(oc(n1)CO)c2cnc(s2)N | Cluster 5# 1 of 5_2 | -7.529602 | 1 | Hybrid | lig_033 | -6.58108711 | 0.948189139 | 4.38566065 |
| c1nc2c(c(=O)n1CC(=O)CO)nn[nH]2 | Cluster 6# 5 of 14_7 | -7.525311 | 1 | Hybrid | lig_034 | -6.72235823 | 0.888356626 | 4.08080864 |
| c1c2c(cnc(n2)N)[nH]c1C(=O)CO | Cluster 13# 2 of 9_1 | -7.474918 | 1 | Hybrid | lig_035 | -7.09482098 | 0.930983663 | 4.61799335 |
| c1cnc(c2c1n(cn2)C[C@@H](CO)O)O | Query Analogs# 1 of 18_25 | -7.452562 | 1 | Hybrid | lig_036 | -7.34787178 | 0.795304835 | 4.21307278 |
| c1cn(c2c1c(ncn2)N)C[C@H](CCO)CO | Cluster 24# 2 of 2_166 | -7.445775 | 1 | Hybrid | lig_037 | -7.43182468 | 0.956655204 | 4.87118244 |
| c1nc2c(c(n1)O)ncn2CC[C@@H](CO)O | Cluster 8# 11 of 20_45 | -7.438823 | 1 | Hybrid | lig_038 | -7.22324133 | 0.82006526 | 4.26305485 |
| c1c(ncnc1N(CCO)CCO)N | Cluster 22# 4 of 7_21 | -7.416665 | 1 | Hybrid | lig_039 | -6.37249851 | 0.77356869 | 4.01353073 |
| Cn1c(cc2c1cnc(c2)O)C(=O)CO | Cluster 13# 1 of 9_6 | -7.40532 | 1 | Hybrid | lig_040 | -7.33744526 | 0.745212138 | 4.12571907 |
| c1cn(c2c1c(ncn2)O)COCCO | Cluster 12# 9 of 14_28 | -7.401099 | 1 | Hybrid | lig_041 | -7.11004782 | 0.857649863 | 4.084764 |
| c1c2cc(ncc2cc(c1CO)O)CO | Cluster 3# 1 of 1_4 | -7.395615 | 1 | Hybrid | lig_042 | -7.61268663 | 0.808205366 | 4.11292028 |
| C[C@@H](CCn1c2c(c(ncn2)N)nn1)CO | Cluster 8# 12 of 20_64 | -7.382172 | 1 | Hybrid | lig_043 | -6.94830704 | 0.94875747 | 4.72890043 |
| c1cnc(c2c1n(cn2)C[C@@H](CO)O)N | Query Analogs# 2 of 18_7 | -7.350226 | 1 | Hybrid | lig_044 | -7.22373915 | 0.877045631 | 4.39806652 |
| CCCN(CCO)c1cc(ncn1)N | Cluster 22# 2 of 7_133 | -7.347079 | 1 | Hybrid | lig_045 | -6.35865974 | 0.678753316 | 4.1425643 |
| CC[C@@H](CCn1ccc2c1ncnc2O)CO | Cluster 8# 19 of 20_183 | -7.340506 | 1 | Hybrid | lig_046 | -7.00982571 | 0.700115621 | 4.42585516 |
| C1CO[C@@H](C[N@]1c2nnc(s2)N)CO | Cluster 15# 1 of 1_12 | -6.125558 | 3 | Hybrid | lig_047 | -6.38005447 | 0.410411388 | 3.64513731 |
| C(CNc1c(c(ns1)O)C#N)CO | Cluster 36# 15 of 21_16 | -6.7203 | 5 | Hybrid | lig_048 | -6.08899403 | 0.673032343 | 3.85907888 |
| c1nc2c(c(n1)O)ncn2CCC(CO)CO | Cluster 8# 1 of 20_72 | -7.294199 | 1 | Hybrid | lig_049 | -7.18608189 | 0.77327323 | 4.30766869 |
| c1cn(c2c1c(ncn2)O)CC[C@@H](CO)O | Cluster 8# 14 of 20_46 | -7.206278 | 1 | Hybrid | lig_050 | -7.50710154 | 0.731395662 | 4.13894892 |
| COc1ccc2c(c1CCO)ccnc2N | Cluster 33# 1 of 1_7 | -7.180144 | 1 | Hybrid | lig_051 | -7.72124863 | 0.966804504 | 4.78856468 |
| CCN(CCO)c1c2cnc(n2ccn1)N | Cluster 21# 1 of 1_49 | -5.841491 | 2 | Hybrid | lig_052 | -6.2104888 | 0.66668582 | 4.4061389 |
| c1nc(c2c(n1)n(cn2)CC[C@@H](CO)[NH3+])N | Cluster 8# 4 of 20_15 | -7.117665 | 1 | Hybrid | lig_053 | -7.0060854 | 0.882810116 | 4.43926191 |
| c1nc(c(c(n1)OCCO)C(F)F)N | Cluster 1# 13 of 22_29 | -7.116349 | 1 | Hybrid | lig_054 | -6.59331417 | 0.904565692 | 4.20970869 |
| c1c(c2c(c(n1)N)ncn2C[C@@H](CO)O)F | Query Analogs# 7 of 18_7 | -7.113031 | 1 | Hybrid | lig_055 | -7.25275087 | 0.909727275 | 4.77556419 |
| CCN(c1ccnc(c1)N)C(=O)CO | Cluster 39# 1 of 5_27 | -7.102018 | 1 | Hybrid | lig_056 | -6.94442606 | 0.956382811 | 4.56801081 |
| c1cn2c(cnc2c(n1)O)C(=O)CO | Cluster 9# 1 of 17_5 | -7.098761 | 1 | Hybrid | lig_057 | -6.67275143 | 0.767000735 | 4.07853794 |
| C[C@@H](CCO)Cn1cnc2c1ncnc2O | Cluster 12# 4 of 14_41 | -6.030396 | 3 | Hybrid | lig_058 | -6.75698566 | 0.616753399 | 4.18204498 |
| C[C@@H](CCn1cnc2c1ccnc2N)CO | Cluster 8# 10 of 20_38 | -7.094102 | 1 | Hybrid | lig_059 | -6.98793077 | 0.969418705 | 5.00379562 |
| c1nc(c2c(n1)n(cn2)CC[C@@H](CO)O)N | Cluster 8# 13 of 20_45 | -7.092673 | 1 | Hybrid | lig_060 | -7.26820421 | 0.912516594 | 4.37913418 |
| C[C@@H](CCO)Cn1ccc2c1ncnc2N | Cluster 12# 6 of 14_8 | -7.092441 | 1 | Hybrid | lig_061 | -7.15511179 | 0.90026325 | 4.71714783 |
| c1cnc(c2c1[C@H](OC2=O)CCO)N | Cluster 18# 1 of 2_4 | -7.088654 | 1 | Hybrid | lig_062 | -7.29943705 | 0.903215408 | 4.06765842 |
| c1cn(c2c1c(ncn2)N)COCCO | Cluster 12# 8 of 14_17 | -7.030166 | 1 | Hybrid | lig_063 | -6.89522505 | 0.947766483 | 4.39872456 |
| c1c(csc1CO)c2cc(n[nH]2)N | Cluster 5# 5 of 5_9 | -4.156595 | 7 | Hybrid | lig_064 | -6.91649532 | 0.925889671 | 4.44160032 |
| c1nc(c(c(n1)OCCO)C(=O)N)N | Cluster 21# 1 of 2_10 | -6.609879 | 2 | Hybrid | lig_065 | -6.85516262 | 0.945875168 | 4.10985565 |
| CCc1c2c(nccn2c(n1)CCO)N | Cluster 6# 3 of 8_3 | -6.934601 | 1 | Hybrid | lig_066 | -6.78553247 | 0.94961971 | 4.99322701 |
| c1cnc(c(c1OCCO)C#N)N | Cluster 9# 2 of 5_28 | -6.912351 | 1 | Hybrid | lig_067 | -6.21151066 | 0.948726535 | 4.23903084 |
| c1nc(c(c(n1)N)O)CCCO | Cluster 1# 19 of 22_8 | -6.694983 | 2 | Hybrid | lig_068 | -6.25819969 | 0.861670256 | 4.07701159 |
| c1cc(ncc1[C@H]2C[C@@H]2CO)O | Cluster 27# 1 of 1_1 | -5.772218 | 3 | Hybrid | lig_069 | -6.67426395 | 0.816301048 | 4.0011425 |
| CCCCN(CCO)c1ccnc(n1)N | Cluster 22# 6 of 7_232 | -6.349218 | 3 | Hybrid | lig_070 | -6.40186739 | 0.399182528 | 3.99203873 |
| C=CC[n+]1cn(c2c1c(ncn2)N)C[C@@H](CO)O | Cluster 5# 1 of 1_102 | -6.379847 | 7 | Hybrid | lig_071 | -7.19770336 | 0.959713817 | 4.95770788 |
| c1c(c2c(c(n1)N)ncn2C[C@@H](CO)O)Cl | Query Analogs# 6 of 18_17 | -6.800924 | 1 | Hybrid | lig_072 | -7.30956173 | 0.933846056 | 4.88824081 |
| c1c2c(c(nn1)N)ncn2C[C@@H](CO)O | Query Analogs# 9 of 18_24 | -6.764225 | 1 | Hybrid | lig_073 | -7.3689189 | 0.918596983 | 4.37186146 |
| c1c(ncnc1N(CCO)CC#N)N | Cluster 22# 3 of 7_24 | -6.761584 | 1 | Hybrid | lig_074 | -6.46756697 | 0.929391146 | 4.3853879 |
| C[C@@H](CCn1cnc2c1ncnc2NC)CO | Cluster 12# 11 of 14_126 | -6.756663 | 1 | Hybrid | lig_075 | -6.72708845 | 0.944482923 | 4.82813835 |
| c1cc2c(c(c1CC(=O)CO)F)oc(n2)N | Cluster 6# 8 of 14_31 | -6.755612 | 1 | Hybrid | lig_076 | -7.45935774 | 0.946934283 | 4.82906818 |
| c1c(c2ncnc(n2n1)O)C(=O)CO | Cluster 9# 16 of 17_1 | -6.736487 | 1 | Hybrid | lig_077 | -7.0299325 | 0.672105074 | 3.80348659 |
| c1cn(c2c1c(ncn2)N)CC[C@@H](CO)O | Cluster 8# 15 of 20_40 | -6.721063 | 1 | Hybrid | lig_078 | -7.57461834 | 0.902448833 | 4.46778107 |
| c1cc2c(ccnc2c(c1)O)C[C@@H](CO)O | Cluster 4# 1 of 3_3 | -6.698925 | 1 | Hybrid | lig_079 | -8.2762661 | 0.905652821 | 4.63704634 |
| C=C(CO)[C@@H]1Cc2cnc(nc2C1)N | Cluster 2# 4 of 6_14 | -4.786986 | 6 | Hybrid | lig_080 | -7.33615923 | 0.515318394 | 4.07569504 |
| c1c(c2c(s1)c(ncn2)N)C(=O)CO | Cluster 9# 9 of 17_5 | -6.679532 | 1 | Hybrid | lig_081 | -7.08253813 | 0.978084326 | 4.81494999 |
| c1nc2c(c(n1)O)ncn2C(=O)CO | Cluster 9# 2 of 17_1 | -6.677595 | 1 | Hybrid | lig_082 | -6.81569004 | 0.71010536 | 3.78164816 |
| C=CCn1c[n+](c2c1c(ncn2)N)CCO | Cluster 6# 4 of 8_23 | -6.658514 | 1 | Hybrid | lig_083 | -6.64618349 | 0.958121061 | 4.82407665 |
| CCNc1c2ccn(c2ncn1)C[C@@H](CO)Cl | Cluster 26# 1 of 1_97 | -6.026973 | 7 | Hybrid | lig_084 | -7.42230463 | 0.944191217 | 4.97441721 |
| c1nc(c2c(n1)n(nn2)C(CO)CO)N | Cluster 9# 8 of 17_16 | -6.605869 | 1 | Hybrid | lig_085 | -6.94412088 | 0.958834589 | 4.27251911 |
| c1cc2c(cc1CCO)n[nH]n2 | Cluster 4# 5 of 5_7 | -6.599305 | 1 | Hybrid | lig_086 | -6.88716984 | 0.956826985 | 4.23444653 |
| c1cnc(cc1OCCO)CO | Cluster 43# 11 of 26_26 | -5.08236 | 3 | Hybrid | lig_087 | -5.96940994 | 0.842823505 | 3.74048877 |
| C[C@@H](CCn1cnc2c1ncnc2O)CO | Cluster 8# 6 of 20_37 | -6.566773 | 1 | Hybrid | lig_088 | -6.88893747 | 0.715034544 | 4.24160624 |
| c1cn2c(nnc2c(n1)N)CCO | Cluster 9# 13 of 17_5 | -6.563006 | 1 | Hybrid | lig_089 | -6.64091825 | 0.945683479 | 4.51666164 |
| Cc1c(ncnc1N)[C@@H](CCO)O | Cluster 1# 15 of 22_8 | -6.558969 | 1 | Hybrid | lig_090 | -7.08358383 | 0.957024336 | 4.35245657 |
| c1cn2c(c[nH+]c2c(n1)O)CCO | Cluster 9# 4 of 17_2 | -6.551706 | 1 | Hybrid | lig_091 | -6.72647953 | 0.683100939 | 4.04888296 |
| CC[C@@H](CCn1cnc2c1ncnc2O)CO | Cluster 8# 18 of 20_171 | -6.490114 | 1 | Hybrid | lig_092 | -6.91006994 | 0.641531646 | 4.33177805 |
| c1cnc(cc1c2nnc(s2)N)CO | Cluster 59# 1 of 1_5 | -6.489281 | 1 | Hybrid | lig_093 | -7.24499273 | 0.829557121 | 4.25320196 |
| c1nc(c(c(n1)N)Cl)CCCO | Cluster 1# 5 of 22_16 | -6.48807 | 1 | Hybrid | lig_094 | -6.38527632 | 0.974000871 | 4.68487835 |
| Cc1c(c(cc2c1O[C@H](CC2)CO)O)CO | Cluster 16# 6 of 9_7 | -6.47026 | 1 | Hybrid | lig_095 | -7.8045435 | 0.650067925 | 4.10133219 |
| c1nc2c(nnnc2n1C[C@@H](CO)O)N | Query Analogs# 15 of 18_34 | -5.472806 | 3 | Hybrid | lig_096 | -7.5360465 | 0.885220647 | 4.26970387 |
| Cc1nc2c(n1C[C@@H](CO)O)ncnc2O | Query Analogs# 14 of 18_20 | -5.450176 | 7 | Hybrid | lig_097 | -7.38902617 | 0.683130205 | 4.06 |

^*^Gnina - the Gnina docking software is a deep learning framework for molecular docking that utilizes an ensemble of convolutional neural networks (CNNs) as a scoring function.

Minimized Affinity – is a binding affinity predicted based on empirical scoring functions.

CNN Score - is an assessed probability that the pose is a "good" one (pose quality)
CNN Affinity – is a predicted “binding” affinity in "pK" units, example: 1μM corresponds to
 CNN Affinity of 6, 1nM of 9.

^##^ - the cluster of compounds used for the design of compound 7 (**Cluster 5# 4 of 5_6**). When sorted against decreasing CNN affinity, this cluster appears on the 16^th^ position (19^th^ in case of the HYBRID Chemgauss4 score). Hence, both re-scoring methods yielded similar results, thus could be used interchangeably.

**Table S5**. List of compounds that have been identified using the OpenEye suite as potential CdaA binders. Selected compounds for soaking (S) and docking experiments (D) have been marked.

| **Nr** | **CAS** | **PubChem CID** | **Canonical SMILES** | **SELECTION** |
| --- | --- | --- | --- | --- |
| **1** | **2289-75-0** | **73238** | **C1(=C(C)N=C(S1)N)C** | **S** |
| 2 | N/A | N/A | C1=NC(=C2C(=N1)NCN2CC=C)N |  |
| 3 | N/A | 156529109 | C1=C2C(=C(N=C1)N)C(OC2)=O |  |
| **4** | **22433-68-7** | **21719209** | **C1=NC(=C(C=N1)C)N** | **S** |
| 5 | 9025272 | 76848841 | C1=C[N]C2=NC=NC(=C12)NCC |  |
|  |  |  |  |  |
| 6 | 1580537-21-8 | 76329229 | O=C1OC2(C=3C=CN=C(N)C13)CCCCC2 |  |
| **7** | 1415569-70-8  **32558-17-1** | 71461242  **40480560** | O=C(NC1=NC(=C(S1)C=2OC(=NC2)C(O)(C)C)C)C  **CC1=C(SC(=N1)NC(=O)C)C2=CSC(=N2)N** | D  **S** |
| 8 | 1374985-83-7 | N/A | OCC(O)C(O)CCC=1C=NC=NC1N |  |
| 9 | 2673369-77-0 | N/A | O=C1OC(C)(C)C(C2=NC(N)=CC=C12)C |  |
| 10 | 144867-23-2 | 10353638 | FCC(O)CN1C=NC=2C(=NC(=NC21)N)N |  |
| 11 | 2381864-23-7 | 104276794 | OC(C=1C(=NC=CC1C)N)CCN |  |
| 12 | 2109656-67-7 | N/A | N1=C(SC=C1C=2C=C(OC2C)N)N |  |
| 13 | 2223576-48-3 | N/A | O=C1OC(C=2C=CN=C(NC3C=CC(CO)C3)C12)(C)C |  |
| **14** | **850801-35-3** | [3426161](https://pubchem.ncbi.nlm.nih.gov/compound/3426161) | **CC1CC2=NC(=NC=C2C(=O)C1)N** | **S** |
| 15 | 1409611-04-6 | 64128795 | OC(CC1=NC=CC=C1)CC=2C=CN=C(N)C2 |  |
| **16** | 1516249-92-5  **376349-57-4** | N/A  **21898208** | OCC(O)C1=NN=C(O1)C=2SC(=NC2)N  **CC1=CC(=C(O1)C)C2=CSC(=N2)N** | D  **S** |
|  |  |  |  |  |
| 17 | 1096156-20-5 | 25113172 | CC1=CC(=CC(=C1)NC2=NC=CC(=N2)C3=NC(=C(S3)C(=O)NC(C)CO)C)C |  |
| 18 | 945393-82-8 | 25011731 | C1COCCN1C2=CC=CC(=C2)NC3=NC=CC(=N3)NC4=C(C=CC5=C4OCO5)Cl |  |
| 19 | 1218-98-0 | 135398602 | C1C(=NC2=C(N1)N=C(NC2=O)N)C(C(CO)O)O |  |
| 20 | 62989-33-7 | 135398654 | CC(C(C1CNC2=C(N1)C(=O)NC(=N2)N)O)O |  |
| 21 | 1805833-75-3 | 91844733 | CC(C)C1=C2N=C(C=C(N2N=C1)NCC3=CC=CC=C3)NCC4CCNCC4O |  |
|  |  |  |  |  |
| 22 | 2650765-48-1 | 17754095 | CC1=CC(=CNC1=O)C2CC(C(O2)COP(=O)(O)O)O |  |
| 23 | 40925-28-8 | 100252 | C1=NC(=NN1C2C(C(C(O2)COP(=O)(O)O)O)O)C(=O)N |  |
| 24 | 3031-94-5 | 65110 | C1=NC(=C(N1C2C(C(C(O2)COP(=O)(O)O)O)O)N)C(=O)N |  |
| 25 | 62025-48-3 | 131704184 | C1=NC(=C(N1C2C(C(C(O2)COP(=O)(O)[O-])O)O)[O-])C(=O)N |  |
| 26 | 964-26-1 | 65063 | C1C(C(OC1N2C=CC(=O)NC2=O)COP(=O)(O)O)O |  |
| 27 | 104086-76-2 | 446696 | C1CC(OC1COP(=O)(O)O)N2C=CC(=NC2=O)N |  |
| 28 | 3715-64-8 | 165151 | CC1=CN(C(=O)NC1=O)C2CCC(O2)COP(=O)(O)O |  |
| 29 | 25520-83-6 | 447405 | CC1=CN(C(=O)NC1=O)C2CC(C(O2)COP(=O)(O)O)F |  |
| 30 | 1032-65-1 | 13945 | C1C(C(OC1N2C=CC(=NC2=O)N)COP(=O)(O)O)O |  |
| 31 | 50000 | 449489 | C1C(C(OC1N2C=C(C(=O)NC2=O)I)COP(=O)(O)O)O |  |
| 32 | 137017-46-0 | 15081829 | C1C(C(OC1N2C=C(C(=NC2=O)N)C=O)COP(=O)(O)O)O |  |
| 33 | 58-97-9 | 6030 | C1=CN(C(=O)NC1=O)C2C(C(C(O2)COP(=O)(O)O)O)O |  |
| 34 | 80860-82-8 | 6505368 | C1C(C(OC1N2C=C(C(=O)NC2=O)C=CBr)COP(=O)(O)O)O |  |
| 35 | 1021540-40-8 | 449076 | C1C(C(OC1N2C=CC(NC2=O)O)COP(=O)(O)O)O |  |
| 36 | 49754-41-8 | 135451578 | C1=C(N=C2C(=O)NC(=NC2=N1)N)COP(=O)(O)O |  |
| 37 | 183204-74-2 | 6323266 | C1CC(=N)N(C1)CC2=C(C(=O)NC(=O)N2)Cl |  |
| 38 | 218144-71-9 | 2797541 | C1=CC(=CC(=C1)[N+](=O)[O-])C2=NOC(=N2)CCCC(=O)O |  |
| 39 | 912444-00-9 | 11960529 | CC1(CCCN1)C2=NC3=C(C=CC=C3N2)C(=O)N |  |
| 40 | 183204-72-0 | [9903778](https://pubchem.ncbi.nlm.nih.gov/compound/9903778) | C1CC(=N)N(C1)CC2=C(C(=O)NC(=O)N2)Cl.Cl |  |

**Table S6**. Summary of Gnina-based rescoring of purchasable compounds (the DrugBank derived compounds have been omitted). Shading corresponds to Table S5.

| **Overall Gnina-based ranking** | **Family of compounds** | **Nr CAS** | **Minimized Affinity** | **CNN score** | **CNN Affinity** |
| --- | --- | --- | --- | --- | --- |
| 1 | 10 | 1415569-71-9 | -6.94816732 | 0.821987391 | 5.39097738 |
| 3 | 10 | 1415569-88-8 | -6.49924612 | 0.938067555 | 5.27684212 |
| 7 | 10 | 1415569-70-8 | -7.71405983 | 0.909892976 | 5.16572046 |
| 11 | 10 | 1415569-72-0 | -6.95304823 | 0.944673598 | 5.05678034 |
| 12 | 10 | 1415569-65-1 | -6.70553541 | 0.934931874 | 5.04609394 |
| 17 | 10 | 1415569-85-5 | -6.60779333 | 0.906725824 | 5.1206007 |
| 21 | 10 | 1415569-68-4 | -7.23536873 | 0.935667157 | 5.00133801 |
| 38 | 30 | 2223576-48-3 | -7.40574121 | 0.938064158 | 4.93256712 |
| 87 | 10 | 1489772-58-8 | -6.69981194 | 0.83483386 | 4.79471827 |
| 96 | 51 | 918510-58-4 | -7.60868502 | 0.921498358 | 4.78 |
| 101 | 20 | 163668-58-4 | -6.28089046 | 0.789070487 | 4.77744865 |
| 162 | 51 | 2026499-58-9 | -6.53264141 | 0.922287166 | 4.70432043 |
| 265 | 51 | 2024964-17-6 | -7.31114483 | 0.956714928 | 4.60662079 |
| 293 | 20 | 2708611-58-7 | -6.47022915 | 0.862541437 | 4.58799458 |
| 315 | 40 | 1409611-04-6 | -7.35524511 | 0.850345492 | 4.57079649 |
| 325 | 51 | 1932058-95-1 | -6.90078259 | 0.971190333 | 4.56460667 |
| 336 | 10 | 1486358-55-7 | -7.48365641 | 0.783985019 | 4.55695868 |
| 379 | 30 | 2673369-77-0 | -7.69705009 | 0.961505651 | 4.53616571 |
| 416 | 10 | 1694400-58-2 | -5.27176142 | 0.94051981 | 4.51686859 |
| 480 | 20 | 2707558-08-3 | -5.93937206 | 0.783980668 | 4.46658516 |
| 490 | 50 | 144867-23-2 | -7.62423182 | 0.917071879 | 4.45926046 |
| 498 | 51 | 90558-56-8 | -6.11190033 | 0.953071415 | 4.45392609 |
| 531 | 51 | 1934598-58-9 | -6.00321674 | 0.977715373 | 4.42626429 |
| 535 | 31 | 1580537-21-8 | -8.31700039 | 0.877735734 | 4.42544222 |
| 559 | 30 | 1580537-39-8 | -6.92573404 | 0.925325394 | 4.40359735 |
| 574 | 10 | 1516249-92-5 | -7.20470381 | 0.934889793 | 4.39470959 |
| 580 | 10 | 2109656-67-7 | -6.53092766 | 0.906095564 | 4.39164162 |
| 628 | 50 | 144867-32-3 | -6.68404913 | 0.950293541 | 4.36111689 |
| 638 | 50 | 69895-58-5 | -7.24106884 | 0.902175725 | 4.35651875 |
| 667 | 40 | 1492558-23-2 | -5.9516449 | 0.872039974 | 4.33552265 |
| 686 | 51 | 92458-14-5 | -5.98485374 | 0.911900878 | 4.32215738 |
| 692 | 31 | 2673369-32-7 | -7.26204157 | 0.890148163 | 4.31843376 |
| 709 | 40 | 2028158-50-9 | -5.79822683 | 0.822802782 | 4.30602789 |
| 772 | 40 | 1374985-83-7 | -7.70738363 | 0.85174334 | 4.26780796 |
| 804 | 30 | 2673369-62-3 | -7.31937599 | 0.934261918 | 4.24636936 |
| 817 | 11 | 2540435-58-1 | -5.88041401 | 0.874972045 | 4.23489714 |
| 833 | 31 | 2535873-58-4 | -7.4451704 | 0.53357619 | 4.21813869 |
| 842 | 10 | 1341958-78-8 | -5.83609676 | 0.783499956 | 4.21069765 |
| 848 | 11 | 15964-04-2 | -5.14600754 | 0.931320429 | 4.20779133 |
| 855 | 31 | 1580537-09-2 | -7.28895855 | 0.88322413 | 4.20262146 |
| 857 | 30 | 2673369-78-1 | -6.13066149 | 0.828316927 | 4.20209312 |
| 860 | 10 | 2167964-43-2 | -7.06868029 | 0.511586487 | 4.19909716 |
| 877 | 31 | 2673369-33-8 | -7.50596237 | 0.722054482 | 4.19297647 |
| 880 | 30 | 1580537-09-2 | -6.99645662 | 0.890385509 | 4.19150829 |
| 883 | 40 | 2352-58-1 | -6.21075392 | 0.780097604 | 4.18971491 |
| 906 | 21 | 2738258-34-7 | -5.89148664 | 0.840480685 | 4.17484045 |
| 916 | 40 | 2380858-87-5 | -7.17364168 | 0.882965565 | 4.16228342 |
| 960 | 11 | 3034-58-0 | -5.03655052 | 0.968779385 | 4.13098001 |
| 974 | 40 | 2381864-23-7 | -7.47053766 | 0.872304499 | 4.11449099 |
| 1004 | 30 | 2673369-03-2 | -7.13979197 | 0.844671726 | 4.09096193 |
| 1078 | 31 | 2673369-40-7 | -6.45240164 | 0.886740923 | 4.04162979 |
| 1086 | 30 | 2673369-40-7 | -6.43533421 | 0.887390316 | 4.03623962 |
| 1111 | 30 | 2673369-64-5 | -6.90651846 | 0.901014149 | 4.1691637 |
| 1179 | 31 | 2673369-49-6 | -6.73394251 | 0.919799805 | 3.95840764 |
| 1195 | 40 | 1339758-37-0 | -5.84097719 | 0.564780354 | 3.93977427 |
| 1196 | 30 | 2673369-68-9 | -7.11214304 | 0.586783886 | 3.93833208 |
| 1205 | 31 | 2673369-36-1 | -6.72280693 | 0.947606087 | 3.93118596 |
| 1247 | 11 | 1378858-51-5 | -5.2447629 | 0.88752681 | 3.88371968 |
| 1288 | 11 | 2289-75-0 | -4.94850063 | 0.946962416 | 3.84668589 |
| 1304 | 30 | 2673369-49-6 | -6.90733719 | 0.673476279 | 3.83291125 |
| 1363 | 41 | 4458-18-8 | -6.08675432 | 0.853373349 | 3.77128983 |
| 1370 | 11 | 2552578-58-0 | -5.42997742 | 0.81085211 | 3.76783752 |
| 1384 | 30 | 217196-58-2 | -6.84344149 | 0.713619292 | 3.75187564 |
| 1398 | 30 | 443921-58-2 | -6.88188314 | 0.738604844 | 3.73727608 |
| 1426 | 31 | 1782558-39-7 | -5.94760799 | 0.937129676 | 3.70176768 |
| 1447 | 41 | 452-58-4 | -5.36254358 | 0.951659381 | 3.66522145 |
| 1451 | 41 | 22433-68-7 | -5.07292366 | 0.929133832 | 3.66057515 |
| 1462 | 30 | 111341-58-3 | -6.71651602 | 0.815446198 | 3.64 |

**Table S7.** Crystallographic data collection and refinement statistics.

|  | **CdaA-APO** | **CdaA-AMP** | **CdaA-ATP** | **CdaA-Adenine** | **CdaA-Adenosine** | **CdaA-comp-4** | **CdaA-comp-7** |
| --- | --- | --- | --- | --- | --- | --- | --- |
| Wavelength [Å] | 0.69 | 0.98 | 0.98 | 0.977 | 0.98 | 0.83 | 0.98 |
| Resolution  range [Å] | 35.61    - 1.45  (1.502    - 1.45) | 45.85    - 1.75 (1.813    - 1.75) | 40.38    - 1.97 (2.04    - 1.97) | 35.68    - 1.55 (1.605    - 1.55) | 40.07    - 1.51 (1.564    - 1.51) | 38.94    - 1.83 (1.895    - 1.83) | 35.68    - 1.2 (1.243    - 1.2) |
| Space group | P 21 21 21 | P 21 21 21 | P 21 21 21 | P 21 21 21 | P 21 21 21 | P 21 21 21 | P 21 21 21 |
| Unit cell | 42.7  64.52  129.48  90 90 90 | 41.61  64.72  129.92  90 90 90 | 42.5  64.65  129.39  90 90 90 | 42.74  64.58  129.65  90 90 90 | 42.14  64.52  129.55  90 90 90 | 40.82  64.55  129.68  90 90 90 | 41.76  64.2  128.78  90 90 90 |
| Total  reflections | 866319 (86971) | 474577 (47442) | 401457 (41641) | 780997 (79214) | 740097 (75495) | 402414 (39808) | 1239840 (44116) |
| Unique  reflections | 63636 (6255) | 36219 (3542) | 25997 (2585) | 52993 (5204) | 56324 (5538) | 31060 (3040) | 106178 (8486) |
| Multiplicity | 13.6 (13.9) | 13.1 (13.4) | 15.4 (16.1) | 14.7 (15.2) | 13.1 (13.6) | 13.0 (13.1) | 11.7 (5.2) |
| Completeness (%) | 98.80 (98.77) | 99.83 (99.75) | 99.68 (99.77) | 99.77 (99.10) | 99.83 (99.57) | 99.68 (99.41) | 97.36 (78.96) |
| Mean I/sigma(I) | 20.10 (1.01) | 16.59 (1.05) | 11.18 (0.73) | 17.23 (0.73) | 16.97 (0.74) | 14.34 (0.84) | 17.26 (0.86) |
| Wilson B[Å^2^] | 26.94 | 38.47 | 46.08 | 31.41 | 27.63 | 37.55 | 15.95 |
| R-merge | 0.05068 (3.081) | 0.0674 (2.875) | 0.1231 (4.58) | 0.06417 (3.774) | 0.06376 (4.131) | 0.09141 (3.498) | 0.05801 (1.417) |
| R-meas | 0.05275 (3.198) | 0.07038 (2.989) | 0.1274 (4.731) | 0.06667 (3.905) | 0.06653 (4.291) | 0.09539 (3.639) | 0.06057 (1.579) |
| R-pim | 0.01441 (0.8457) | 0.01989 (0.8096) | 0.0325 (1.177) | 0.01775 (0.9973) | 0.0187 (1.151) | 0.02682 (0.9924) | 0.01713 (0.6773) |
| CC1/2 | 1 (0.579) | 0.999 (0.707) | 0.998 (0.394) | 0.999 (0.591) | 0.999 (0.538) | 0.999 (0.69) | 0.999 (0.484) |
| CC* | 1 (0.857) | 1 (0.91) | 1 (0.752) | 1 (0.862) | 1 (0.836) | 1 (0.904) | 1 (0.808) |
| Reflections in  refinement | 63559 (6248) | 36216 (3536) | 25974 (2580) | 52977 (5167) | 56308 (5520) | 31049 (3033) | 106139 (8480) |
| Reflections  used for R-free | 3177 (312) | 1810 (178) | 1299 (130) | 2644 (257) | 2815 (277) | 1553 (153) | 5304 (425) |
| R-work | 0.1899 (0.4424) | 0.2215 (0.4981) | 0.2347 (0.5207) | 0.1970 (0.4751) | 0.2061 (0.5161) | 0.2097 (0.4535) | 0.1391 (0.3311) |
| R-free | 0.2173 (0.4707) | 0.2546 (0.5213) | 0.2539 (0.5517) | 0.2356 (0.4878) | 0.2505 (0.5650) | 0.2379 (0.4991) | 0.1626 (0.3269) |
| CC(work) | 0.959 (0.804) | 0.961 (0.804) | 0.968 (0.636) | 0.932 (0.775) | 0.961 (0.760) | 0.969 (0.842) | 0.972 (0.778) |
| CC(free) | 0.971 (0.678) | 0.948 (0.816) | 0.962 (0.626) | 0.935 (0.744) | 0.961 (0.737) | 0.980 (0.750) | 0.974 (0.787) |
| Number of non-hydrogen atoms | 2718 | 2581 | 2456 | 2631 | 2756 | 2482 | 3320 |
| macromolecules | 2466 | 2470 | 2343 | 2406 | 2488 | 2378 | 2790 |
| ligands | 5 | 49 | 91 | 4 | 22 | 19 | 46 |
| solvent | 247 | 62 | 46 | 221 | 246 | 85 | 484 |
| Protein residues | 317 | 318 | 303 | 308 | 319 | 308 | 339 |
| RMS(bonds) [Å] | 0.01 | 0.01 | 0.01 | 0.013 | 0 | 0.01 | 0.01 |
| RMS(angles) [°] | 0.78 | 0.9 | 1.1 | 1.14 | 0.64 | 1.13 | 0.9 |
| Ramachandran  favored (%) | 98.4 | 97.45 | 97.32 | 99.34 | 98.1 | 98.36 | 98.21 |
| Ramachandran allowed (%) | 1.28 | 2.55 | 2.68 | 0.66 | 1.9 | 1.64 | 1.79 |
| Ramachandran  outliers (%) | 0.32 | 0 | 0 | 0.00 | 0 | 0 | 0 |
| Rotamer  outliers (%) | 1.81 | 0 | 6.08 | 2.97 | 0.36 | 0.75 | 0.64 |
| Clashscore | 4.19 | 4.92 | 5.38 | 7.79 | 2.96 | 2.69 | 3.66 |
| Average B-factor [Å^2^] | 40.92 | 58.78 | 68.18 | 47.68 | 46.07 | 54.98 | 24.75 |
| Macromolecules [Å^2^] | 39.57 | 58.48 | 67.74 | 46.36 | 44.09 | 55.08 | 21.7 |
| Ligands [Å^2^] | 66.74 | 68.45 | 83.59 | 66.77 | 71.84 | 54.72 | 25.83 |
| Solvent [Å^2^] | 53.83 | 63.17 | 68.23 | 61.01 | 63.82 | 52.09 | 42.22 |
| TLS groups | 0 | 0 | 10 | 0 | 0 | 8 | 0 |

Statistics for the highest-resolution shell are shown in parentheses.

**
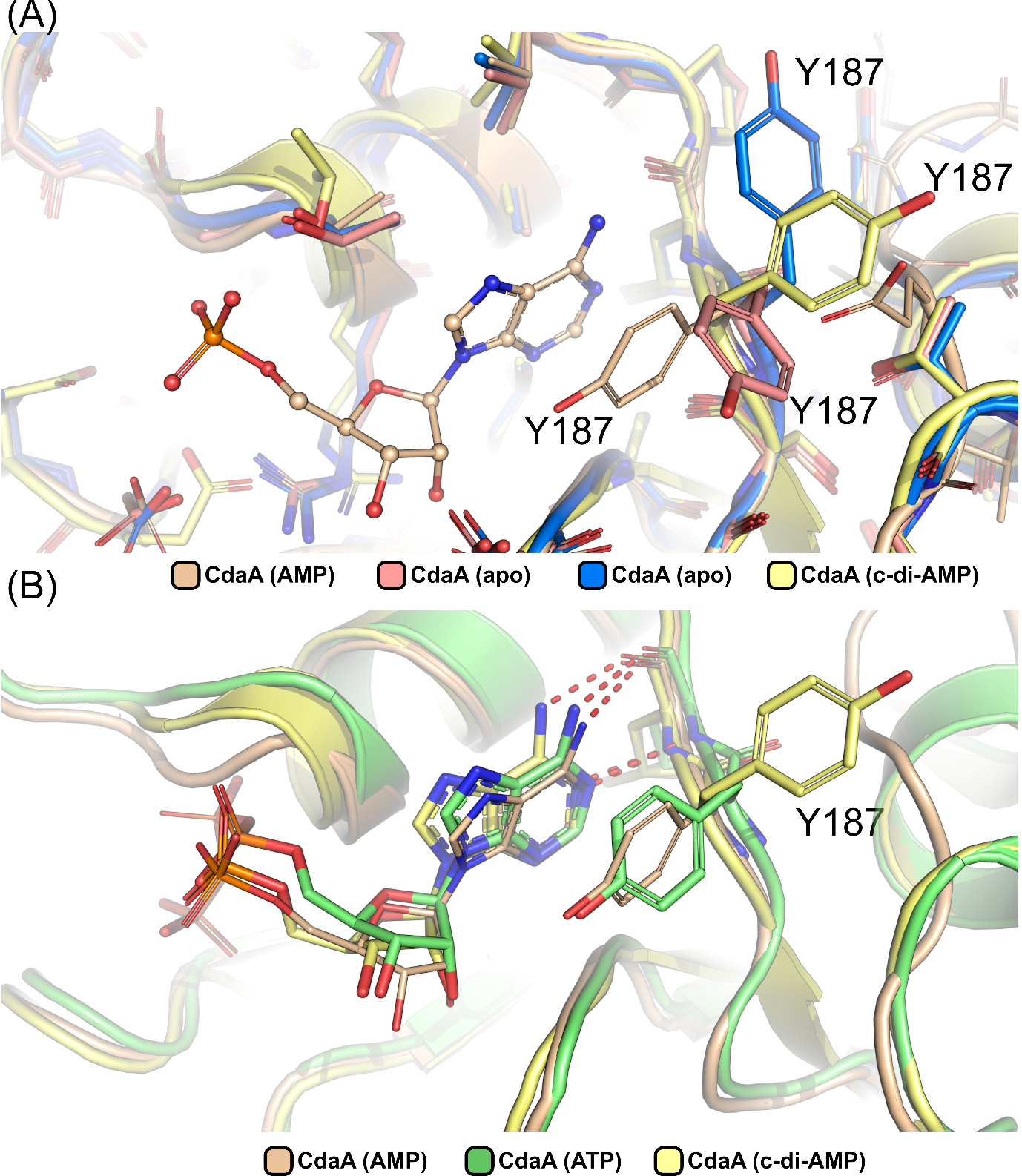
**

**Figure S1**. The active site of CdaA. (**A**) Different side chain orientations of Tyr187 revealing its conformational flexibility in different CdaA crystal structures (complexes) (**B**) Comparison of available *Lm* CdaA complexes with AMP, ATP and the product c-di-AMP. The adenine ring moiety forms two conserved hydrogen bonds depicted as red dashed lines (adenine_N1-Leu188_N, adenine_N7-Leu188_O) with Leu188 of CdaA. The Tyr187 side chain forms stacking interactions with the adenine ring of AMP and ATP but not with the product molecule c-di-AMP.

**
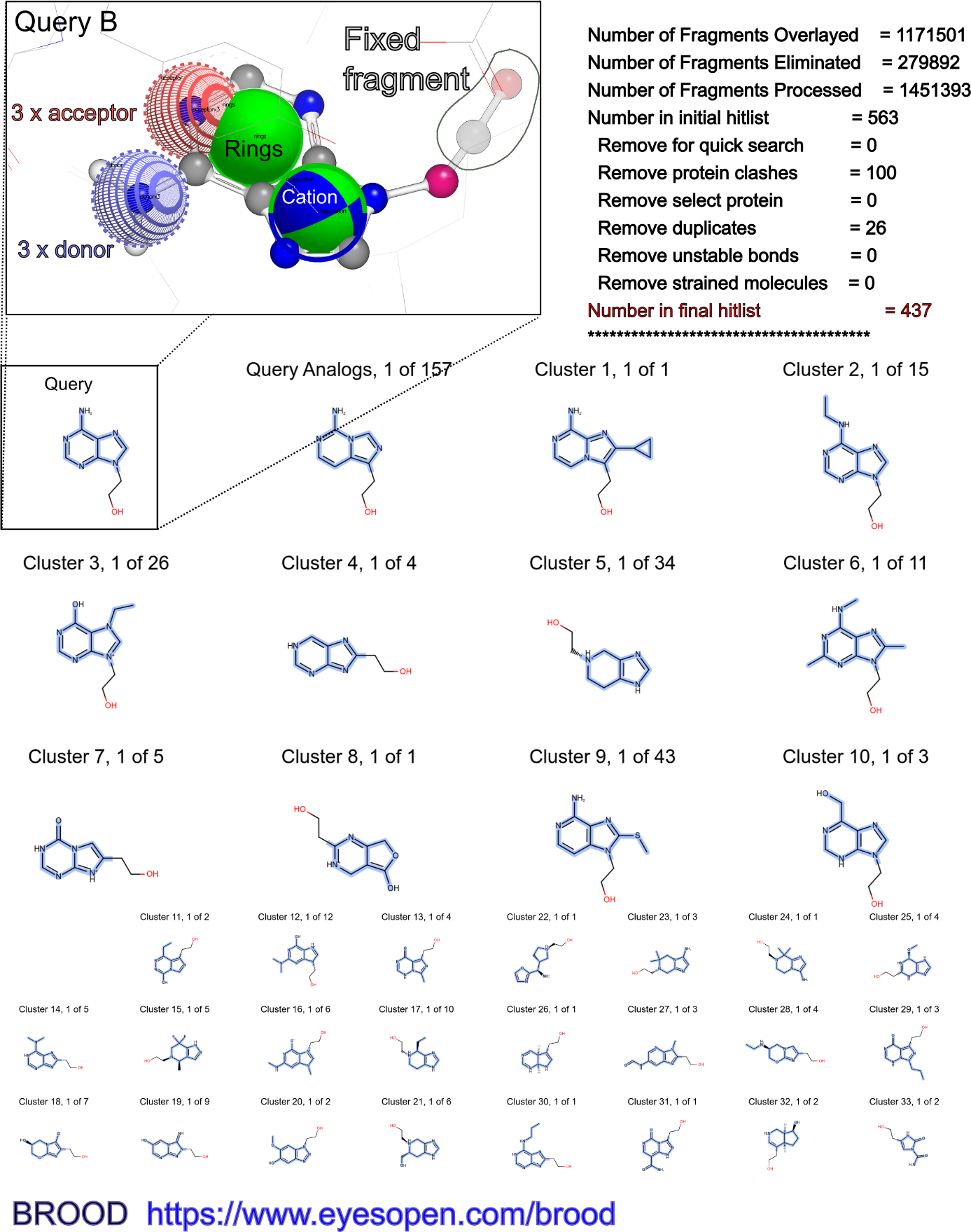
**

**Figure S2**. Graphical summary of an exemplary search performed with BROOD (OpenEye). The Query has been derived from the truncated AMP molecule with a fixed anchoring part marked in grey colour (encircled with a grey line). Chemical properties (constraints) have been marked (donor, acceptor, ring, cation). The importance of chemical properties (weighting) has been achieved by setting it multiple times (3 in that case). The BROOD search results are presented below the Query. Depicted chemical structures of identified ligands reveal quite high similarity to adenine. Further details are presented in Table S1.

**
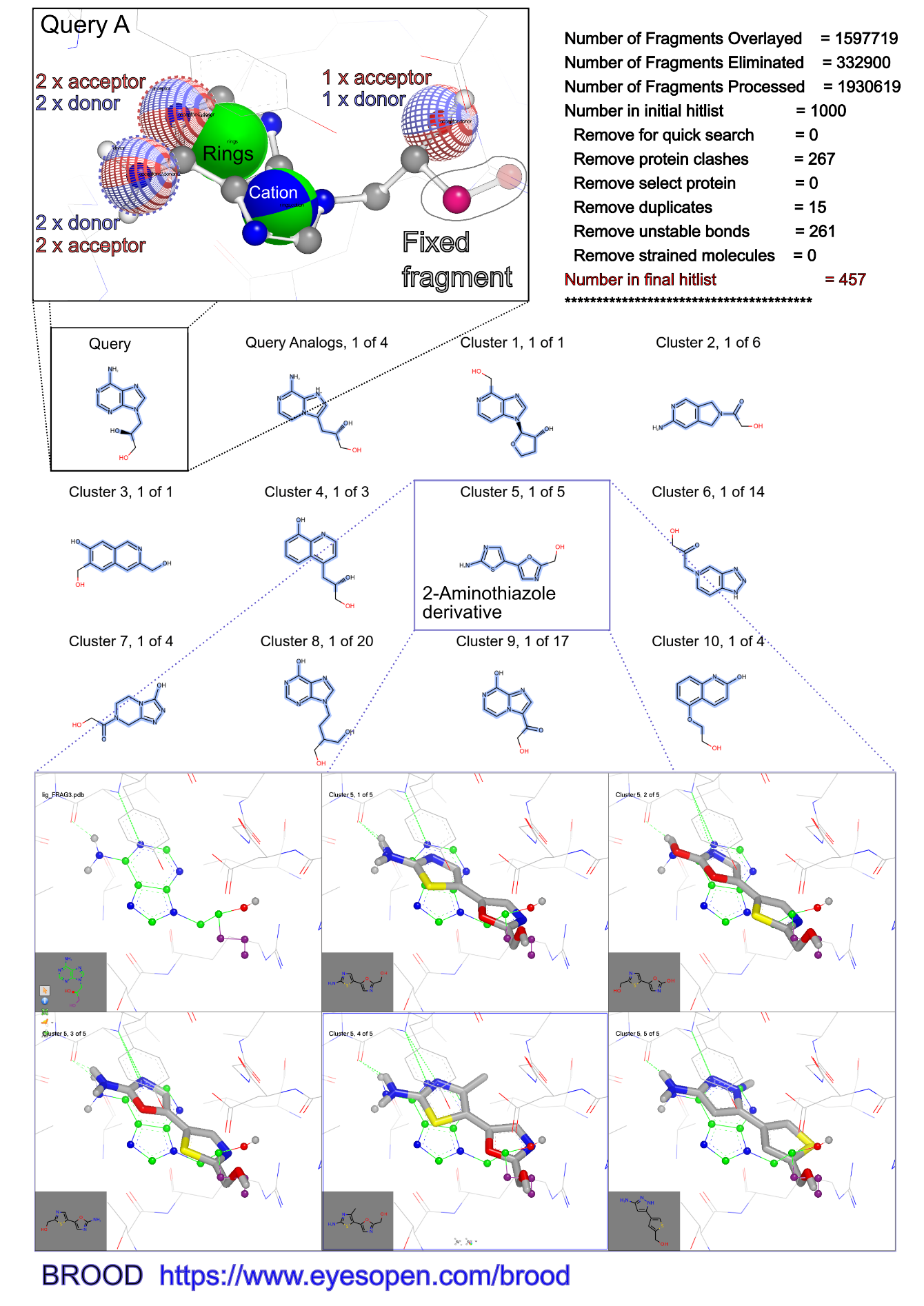
**

**Figure S3**. Graphical summary of a search performed with ROOD (OpenEye) leading to identification of the precursor of compound 7 (Cluster 5, 1 of 5). The Query is derived from the truncated AMP molecule (the fixed anchoring part is encircled with a grey line). Chemical properties (constraints) have been marked (donor, acceptor, ring, cation). The importance of chemical properties has been achieved by setting it multiple times. The best results are presented below the Query. At the bottom, detailed views of compounds comprising 2-aminothiazole derivatives (Cluster 5, 1 of 5) placed in CdaA active site in comparison to the Query molecule (ball-and-sticks model). Further details are presented in Table S2.

**
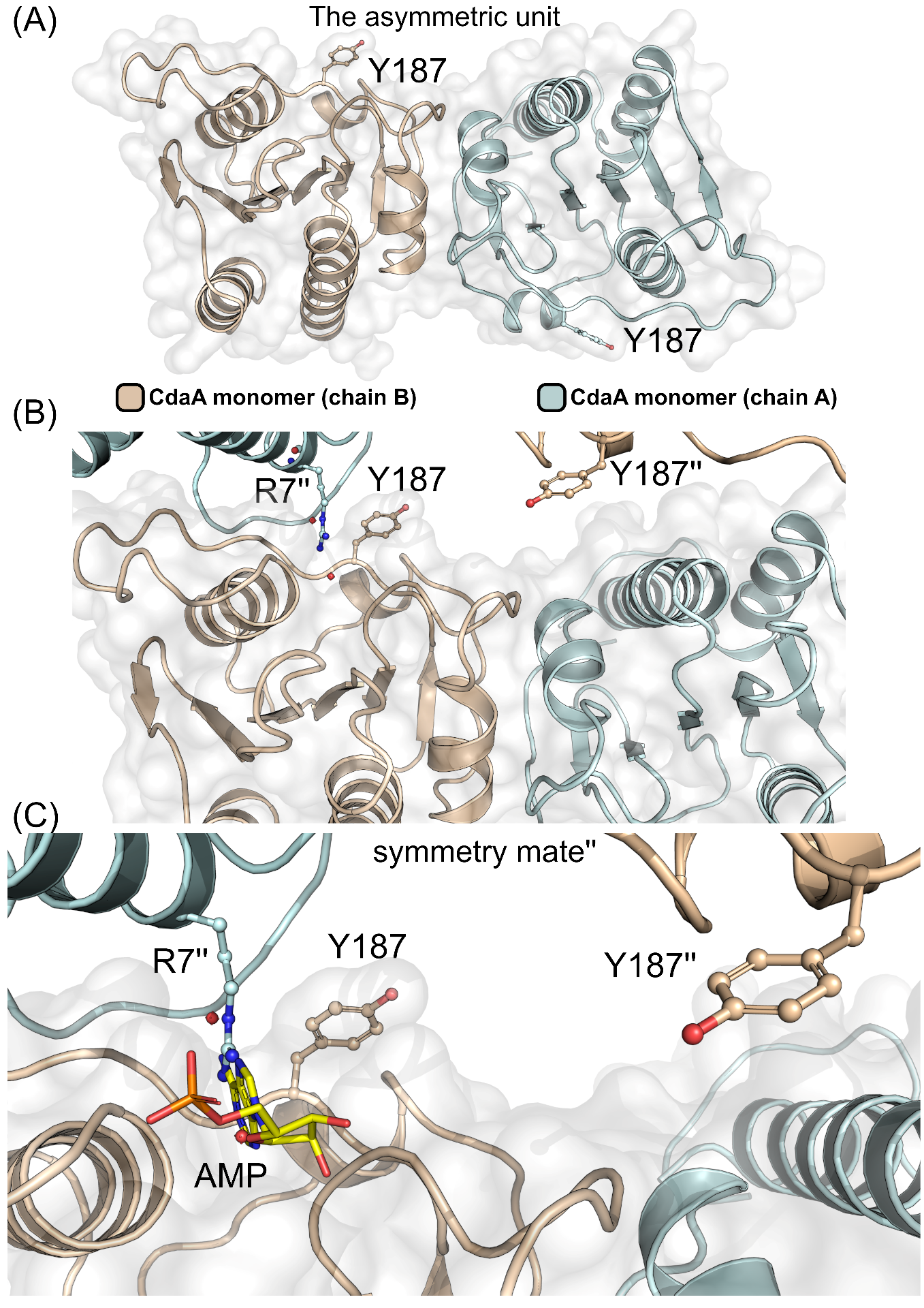
**

**Figure S4**. Non-catalytic “back-to-back” CdaA dimer present in the orthorhombic asymmetric unit. (**A**) The biologically inactive CdaA dimer. Tyr187 is depicted in balls and sticks in order to mark the active site. (**B**) A close-up view of (**A**) with a symmetry related CdaA molecule (top, marked with a ’’ sign). An Arg7 side chain (depicted as balls and sticks) and two water molecules (red spheres) occupy the active site of monomer B in apo CdaA (C) A close-up view of (**B**) with AMP molecule (yellow sticks) marking the active site. Binding of ligand molecule in chain B causes displacement of Arg7’’ side chain (symmetry related molecule) and two water molecules.

**
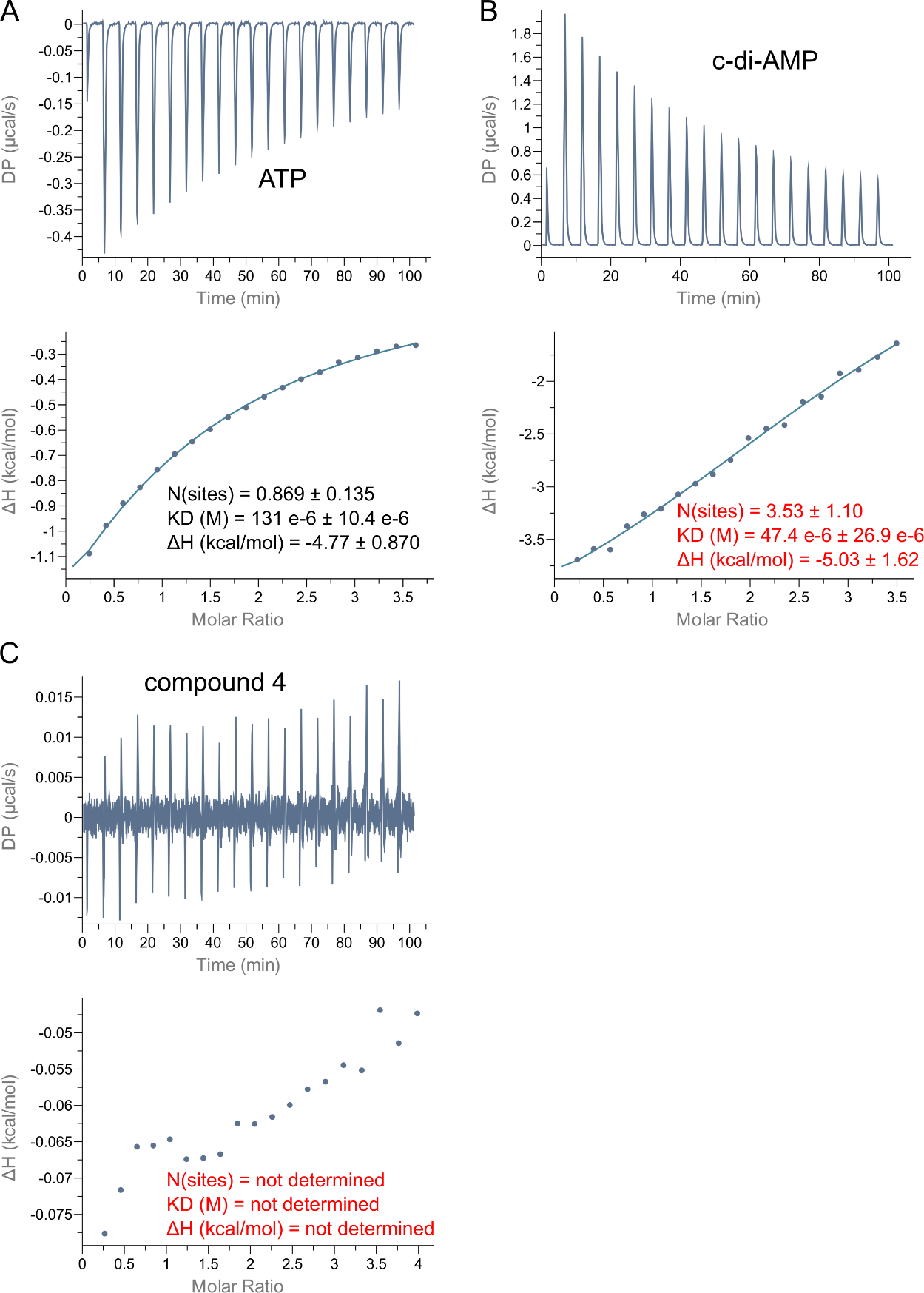
**

**Figure S5**. Isothermal titration calorimeters (ITC) measurements of the natural substrate ATP (**A**), product c-di-AMP (**B**) and compound 4 (**C**) binding to *Lm* CdaA. Both ATP and c-di-AMP experiments have been performed in the presence of Mg^2+^ ions (*Lm* CdaA is not active). For Each titration curve, except the one for compound 4, has been fitted with a 1:1 binding model and yielded the following thermodynamic parameters: the dissociation constant (K_D_), a stoichiometry (N) and the enthalpy of interaction (ΔH) with corresponding errors. For c-di-AMP, the calculated stoichiometry is substantially deviating from theoretical value of 1 (3.53 ± 1.1), indicating that there is not enough information in the curve fitting for all parameters. Hence the assessed K_D_ and ΔH values using the 1:1 binding model are not reliable. In case of compound 4, no fitting was possible.
